# Supplementary material for: UBE2O-mediated ubiquitylation directs cytoplasmic CTNNA1 to promote cell-to-ECM adhesions
Source: EMBO Rep. 2025 Sep 22;26(22):5431–58. doi: 10.1038/s44319-025-00585-4 (PMC12635394; doi:10.1038/s44319-025-00585-4)
Supplement: Supplementary file 1 — Appendix [file 44319_2025_585_MOESM1_ESM.pdf]

Appendix for

UBE2O-mediated ubiquitylation directs cytoplasmic CTNNA1 to promote cell-to-ECM adhesions

Dan Xiang, Wenfeng Wu, Ruona Shi, Xiaoxiao Tang and Xiaofei Zhang*

*Correspondence: zhang_xiaofei@gibh.ac.cn

This PDF file includes:

Appendix Figure S1. UBE2O interacts with CTNNA1. 2

Appendix Figure S2. UBE2O monoubiquitylates CTNNA1. 3

Appendix Figure S3. UBE2O selectively engages cytoplasmic CTNNA1 in FAs rather than AJs. 4

Appendix Figure S4. UBE2O-CTNNA1 axis shows no significant migration-inhibiting effects in MEFs..5

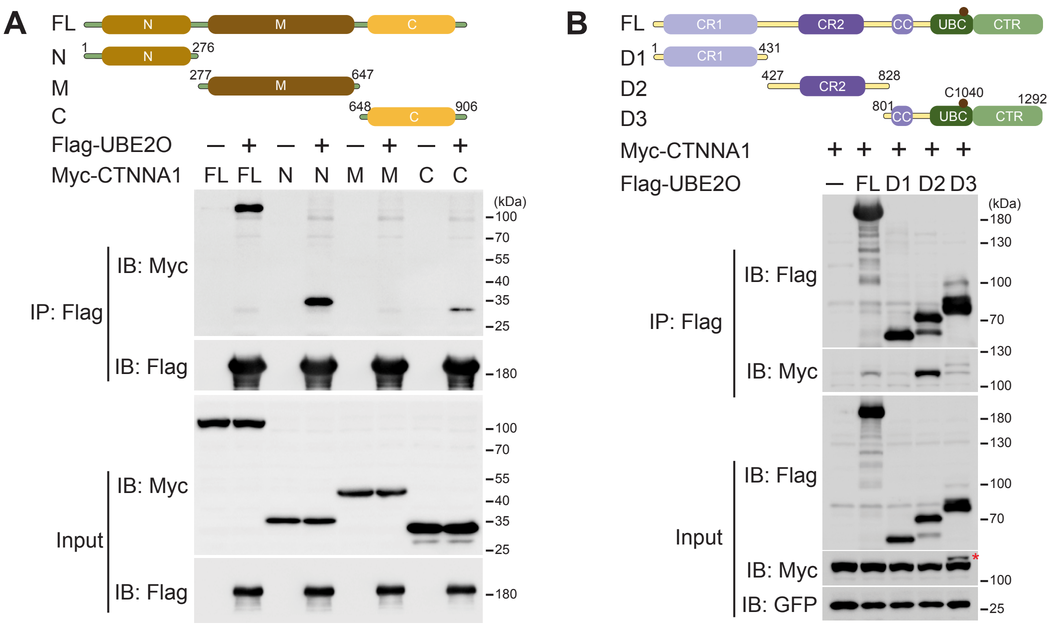

Appendix Figure S1. UBE2O interacts with CTNNA1.

(A) The N-terminal (N) domain of CTNNA1 interacts with UBE2O. The top panel depicts a schematic diagram showing the full-length (FL), N-terminal (N) domain, middle (M) domain and C-terminal (C) domain truncations of CTNNA1. HEK293T cells were transfected with the indicated plasmids for 36 h. Cells were harvested for immunoprecipitation with anti-Flag beads, followed by immunoblotting with an anti-Myc antibody (bottom panel). **(B)** The CR2 domain of UBE2O interacts with CTNNA1. The top panel shows the schematic diagram of the full-length and deletions of UBE2O, the E2 catalytic active site is indicated by a brown dot. HEK293T cells were transfected with the indicated plasmids for 36 h. Cells were harvested for immunoprecipitation with anti-Flag beads and immunoblotting analysis with an anti-Myc antibody (bottom panel). The pLV-GFP plasmid was included as a transfection control. All experiments were repeated at least twice, one representative result is shown.

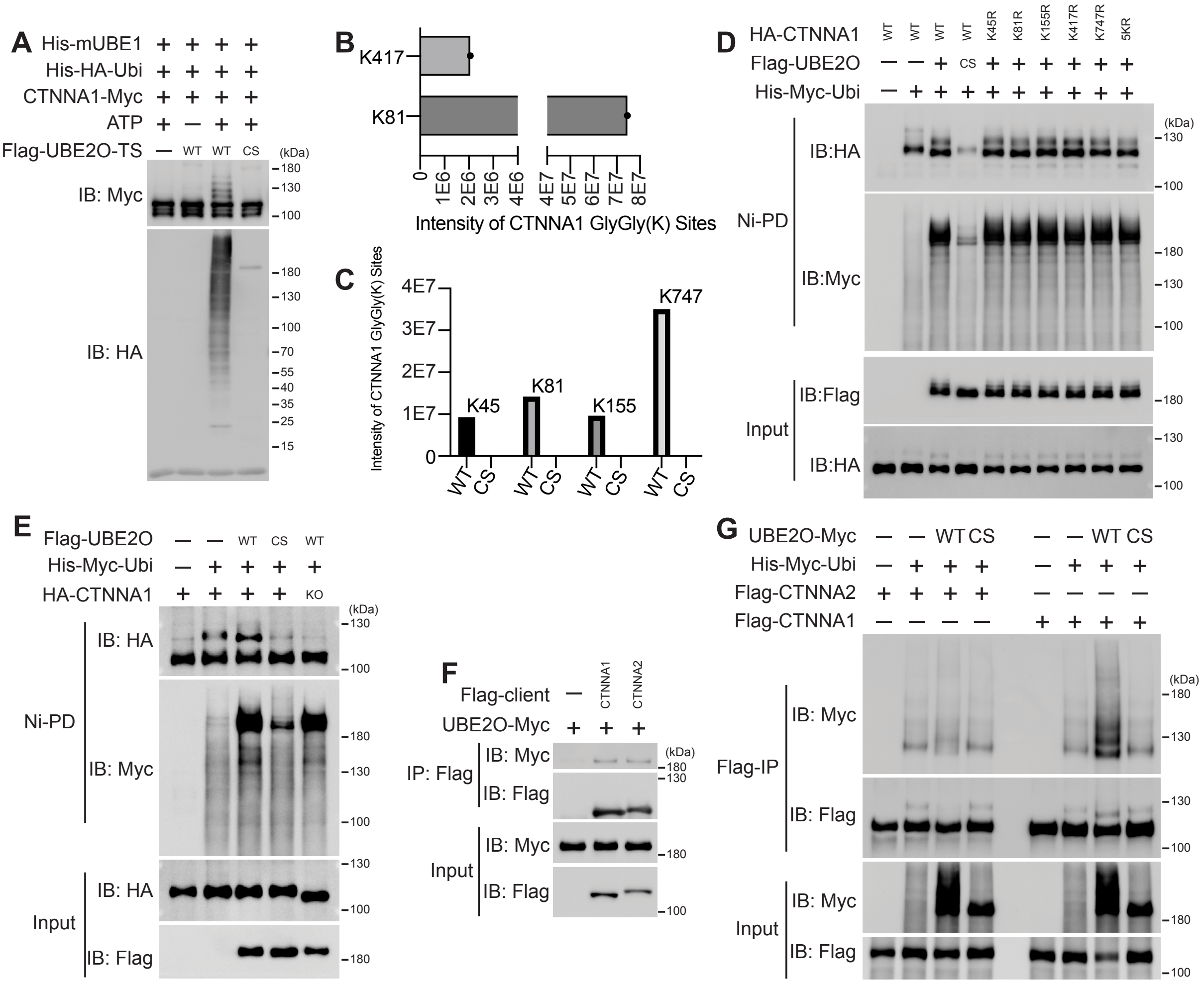

Appendix Figure S2. UBE2O monoubiquitylates CTNNA1.

(A) UBE2O ubiquitylates CTNNA1 *in vitro* and its E2 activity is required for UBE2O-mediated ubiquitylation of CTNNA. Purified proteins were mixed as indicated to perform *in vitro* ubiquitylation assay according to the method. An anti-Myc antibody was used to detect the ubiquitylation of CTNNA1, an anti-HA antibody was used to detect the ubiquitylation of CTNNA1, UBE2O and ubiquitin. **(B)** Barplot showing the intensity of UBE2O-D3 ubiquitylated CTNNA1 peptides and the corresponding sites identified by mass spectrometry. **(C)** Barplot showing the intensity of 4 ubiquitylated CTNNA1 peptides that showed the greatest increase in intensity, along with their corresponding sites according to mass spectrometry data. UBE2O-CS exhibited no detectable ubiquitylation activity towards all 4 peptides (intensity equals 0). **(D and E)** UBE2O ubiquitylates CTNNA1 at multiple lysine residues. HEK293T cells were co-transfected with the indicated plasmids for 36 h. Cells were harvested for Ni-PD ubiquitylation assay and immunoblotting analyses. An anti-HA antibody was used to detect the ubiquitylated CTNNA1. CTNNA1 K417R was included as a negative control. **(F)** Both CTNNA1 and CTNNA2 interact with UBE2O. UBE2O-Myc together with Flag-CTNNA1, Flag-CTNNA2 or Flag-vector were transfected into HEK293T cells as indicated for 36 h. Cells were harvested for co-immunoprecipitation analysis with anti-Flag agarose according to the method. An anti-Myc antibody was used to detect the interaction. **(G)** UBE2O ubiquitylates CTNNA1 but not CTNNA2. HEK293T cells were transfected with the indicated plasmids for 36 h. Cells were harvested for Flag immunoprecipitation ubiquitylation assay. Anti-Myc antibody was used to detect ubiquitylated proteins after Flag-IP procedure. All experiments were repeated at least twice, one representative result is shown.

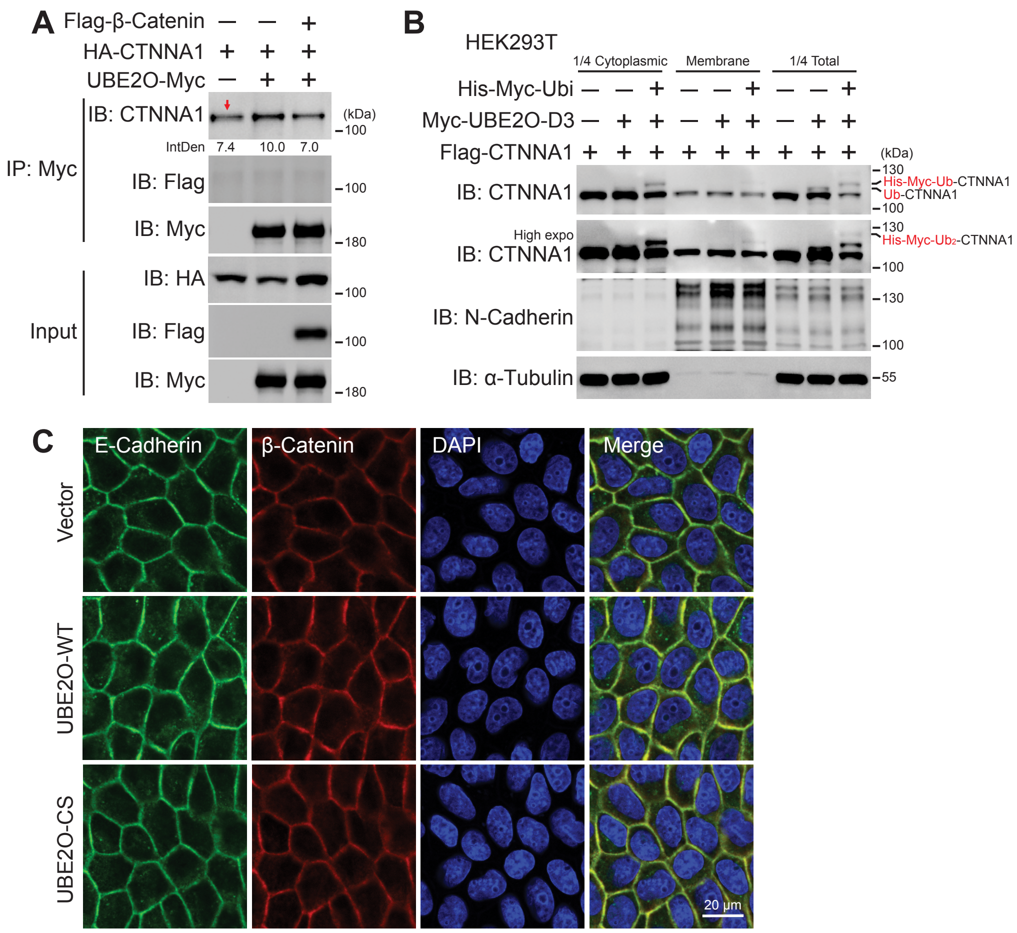

Appendix Figure S3. UBE2O selectively engages cytoplasmic CTNNA1 in FAs rather than AJs.

(A) Co-expression with β-catenin reduces the interaction between UBE2O and CTNNA1 in HEK293T cells. The indicated plasmids were transfected into HEK293T cells for 36 h. Cells were harvested for immunoprecipitation with anti-c-Myc beads and immunoblotting analysis with anti-CTNNA1 antibody. The red arrow indicates unspecific binding to the beads. **(B)** UBE2O-D3 predominantly ubiquitylates the cytosolic fraction of CTNNA1 in HEK293T cells. HEK293T cells were transfected with the indicated plasmids for 48 h. Cells were harvested for subcellular fractionation and western blot analysis according to the methods. An anti-CTNNA1 antibody was used to detect the subcellular distribution of ubiquitylated CTNNA1. Due to the low levels of CTNNA1 in the membrane fraction in HEK293T cells, four times the amount of membrane fraction proteins were loaded for analysis. **(C)** UBE2O overexpression leads to no considerable dissociation of AJs in MDCK cells. Representative micrographs of MDCK cells stably expressing UBE2O-WT, UBE2O-CS mutant or a control vector. Cells were cultured on 8-cell chamber slides for 48 h and immunostained for E-cadherin, β-catenin and DAPI. All experiments were repeated at least twice, one representative result is shown.

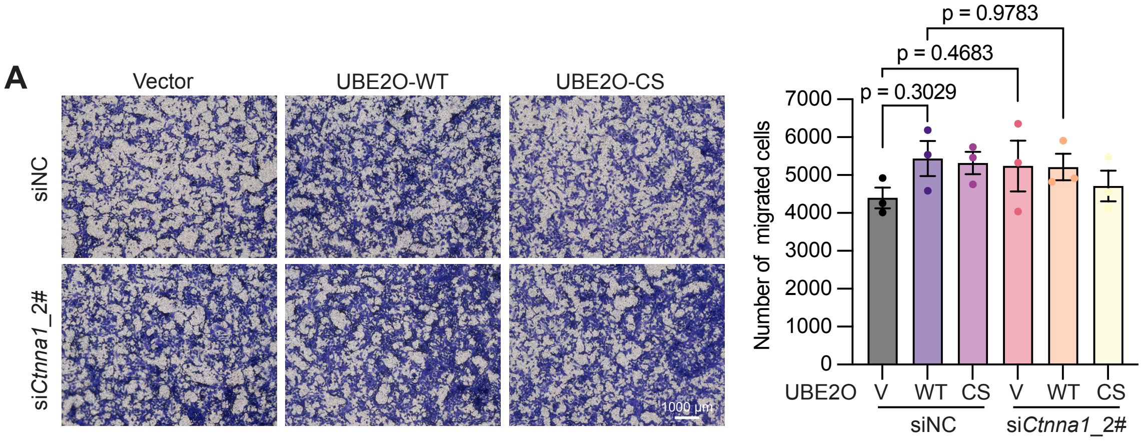

Appendix Figure S4. UBE2O-CTNNA1 axis shows no significant migration-inhibiting effects in MEFs.

(A) Migration ability of UBE2O-overexpressing MEFs following *Ctnna1* knockdown. Transwell assays were conducted according to the method, n=3 technical replicates in independent experiments. Error bars indicate mean ± SEM. Significance among multiple groups was determined using ANOVA followed by Tukey's post hoc test. This experiment was repeated twice, one representative result is shown.
