# Supplementary figures and images for: UBE2O-mediated ubiquitylation directs cytoplasmic CTNNA1 to promote cell-to-ECM adhesions
Source: EMBO Rep. 2025 Sep 22;26(22):5431–58. doi: 10.1038/s44319-025-00585-4 (PMC12635394; doi:10.1038/s44319-025-00585-4)

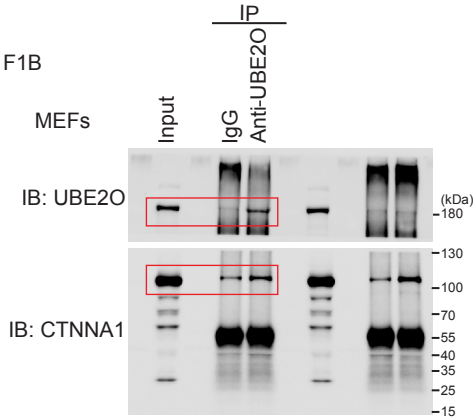

Supplement: Supplementary file 5 — Source data Fig. 1 [file 44319_2025_585_MOESM5_ESM.zip › EMBOR202561827V2_SourceDataForFigure1/1B/Figure1B_Blots.pdf]

F1E

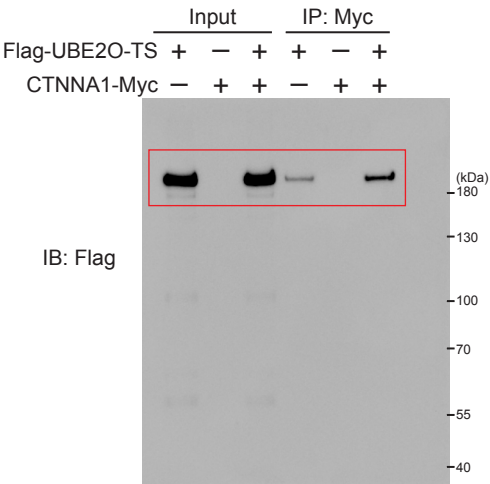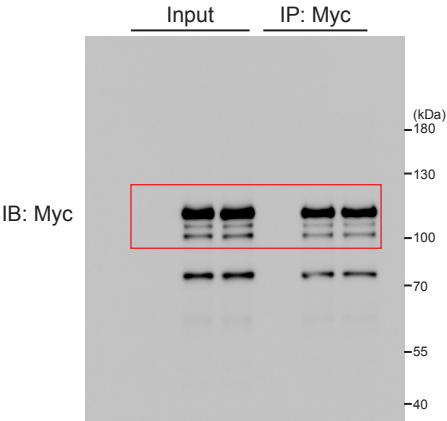

Supplement: Supplementary file 5 — Source data Fig. 1 [file 44319_2025_585_MOESM5_ESM.zip › EMBOR202561827V2_SourceDataForFigure1/1E/Figure1E_Blots.pdf]

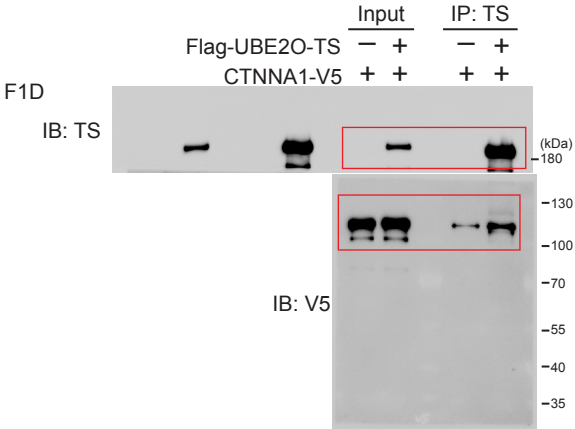

Supplement: Supplementary file 5 — Source data Fig. 1 [file 44319_2025_585_MOESM5_ESM.zip › EMBOR202561827V2_SourceDataForFigure1/1D/Figure1D_Blots.pdf]

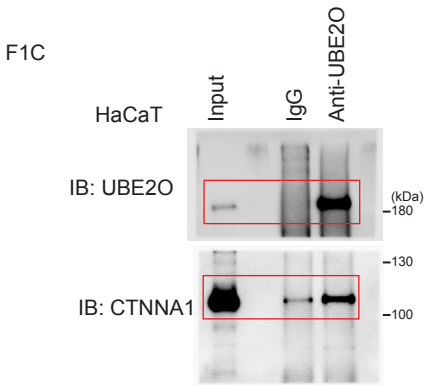

Supplement: Supplementary file 5 — Source data Fig. 1 [file 44319_2025_585_MOESM5_ESM.zip › EMBOR202561827V2_SourceDataForFigure1/1C/Figure1C_Blots.pdf]

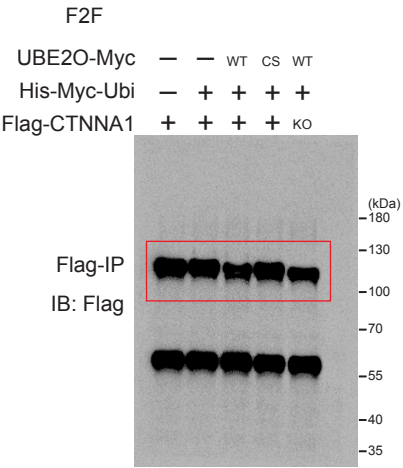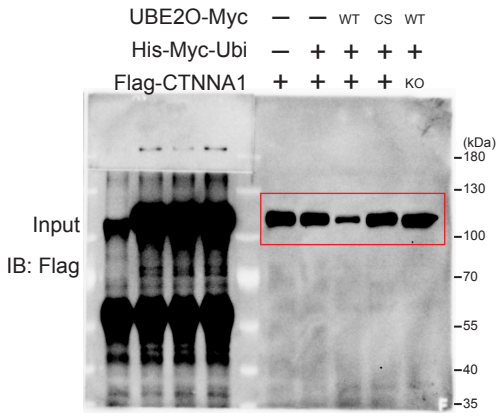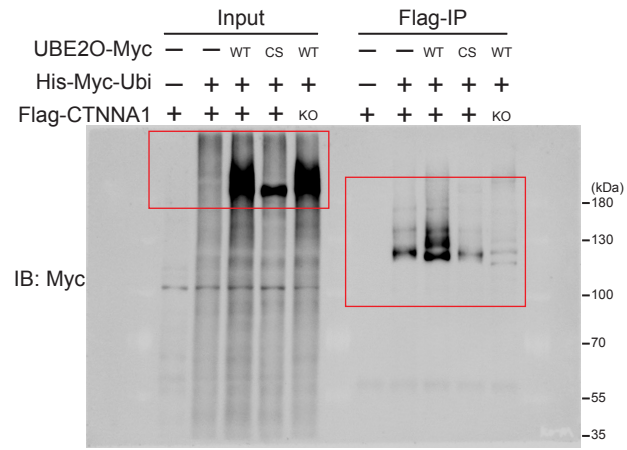

Supplement: Supplementary file 6 — Source data Fig. 2 [file 44319_2025_585_MOESM6_ESM.zip › EMBOR202561827V2_SourceDataForFigure2/2F/Figure2F_Blots.pdf]

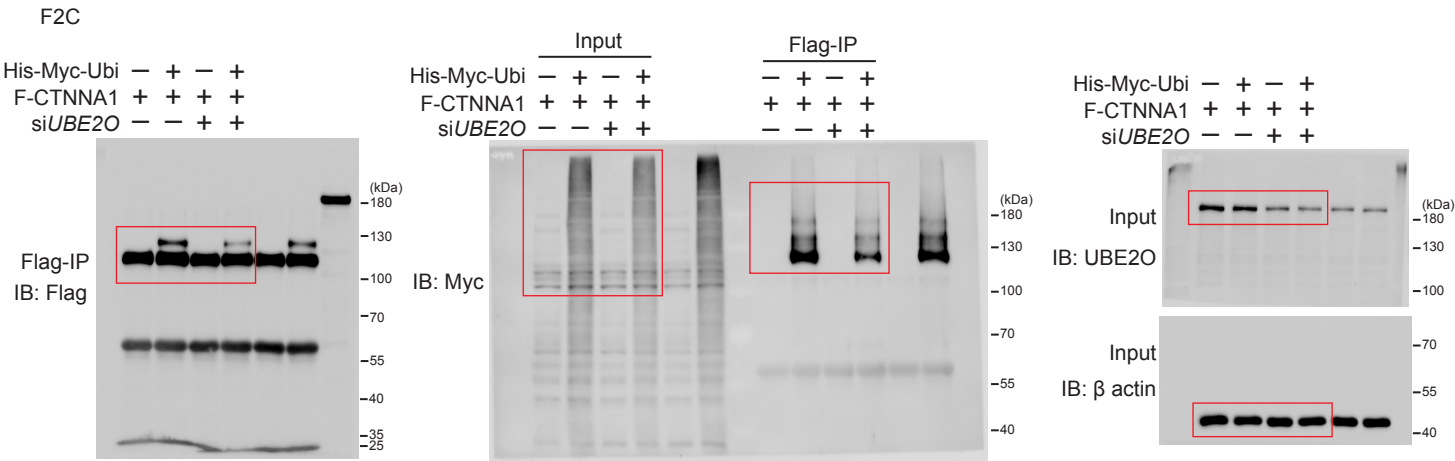

Supplement: Supplementary file 6 — Source data Fig. 2 [file 44319_2025_585_MOESM6_ESM.zip › EMBOR202561827V2_SourceDataForFigure2/2C/Figure2C_Blots.pdf]

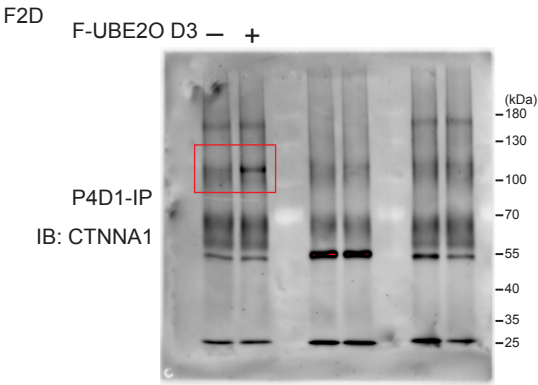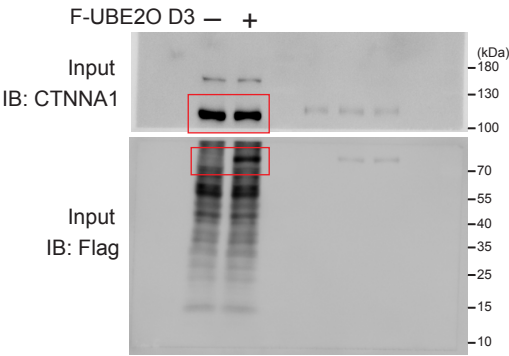

Supplement: Supplementary file 6 — Source data Fig. 2 [file 44319_2025_585_MOESM6_ESM.zip › EMBOR202561827V2_SourceDataForFigure2/2D/Figure2D_Blots.pdf]

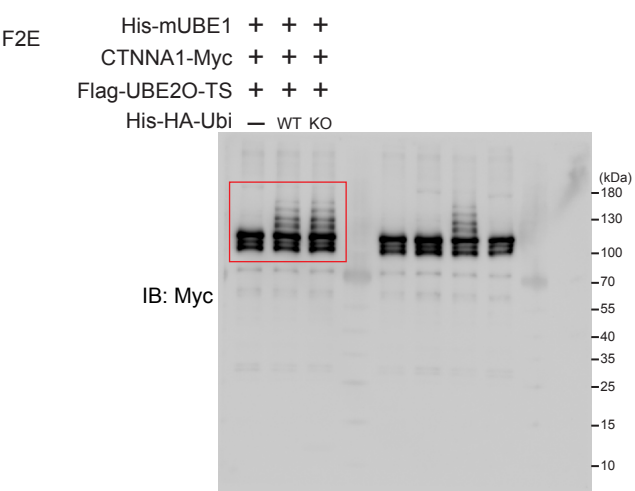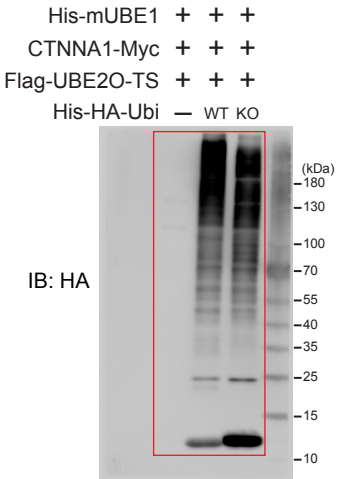

Supplement: Supplementary file 6 — Source data Fig. 2 [file 44319_2025_585_MOESM6_ESM.zip › EMBOR202561827V2_SourceDataForFigure2/2E/Figure2E_Blots.pdf]

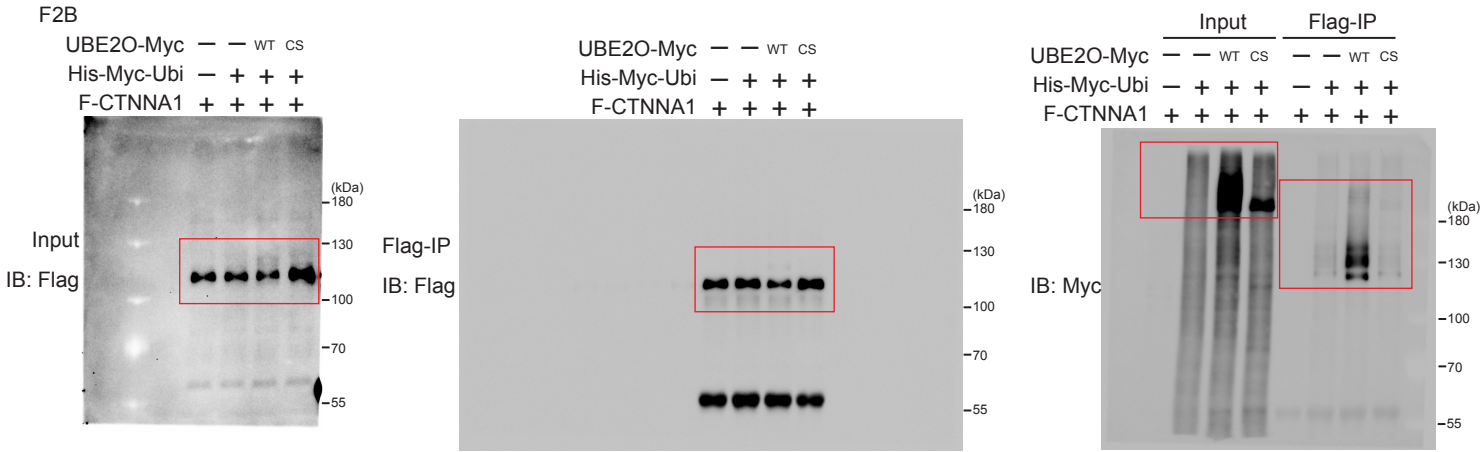

Supplement: Supplementary file 6 — Source data Fig. 2 [file 44319_2025_585_MOESM6_ESM.zip › EMBOR202561827V2_SourceDataForFigure2/2B/Figure2B_Blots.pdf]

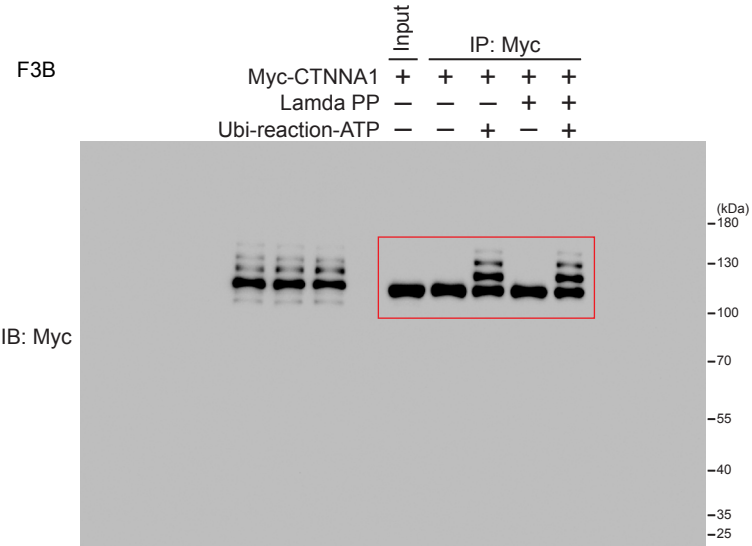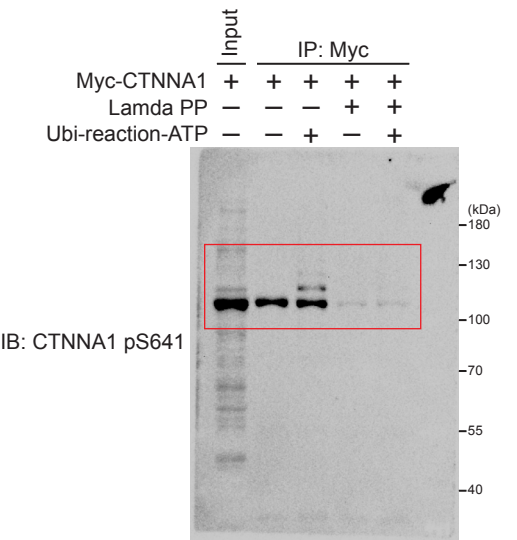

Supplement: Supplementary file 7 — Source data Fig. 3 [file 44319_2025_585_MOESM7_ESM.zip › EMBOR202561827V2_SourceDataForFigure3/3B/Figure3B_Blots.pdf]

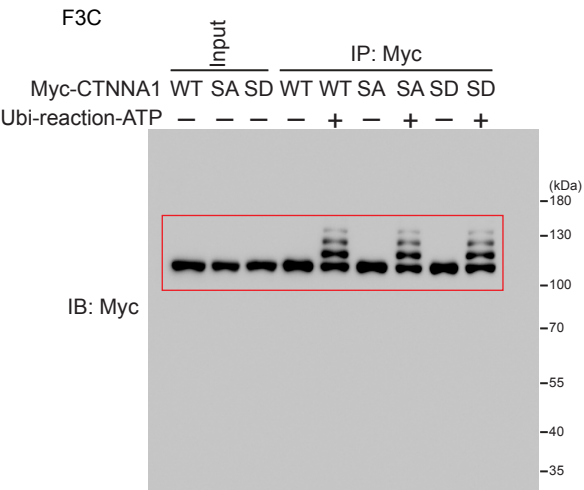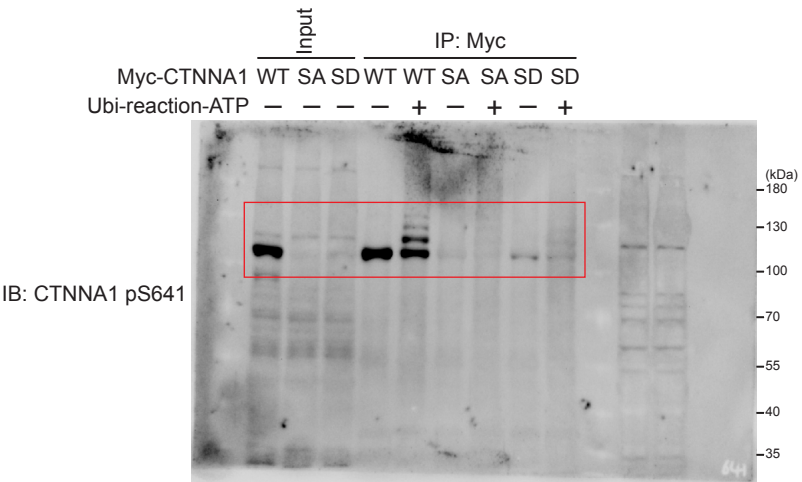

Supplement: Supplementary file 7 — Source data Fig. 3 [file 44319_2025_585_MOESM7_ESM.zip › EMBOR202561827V2_SourceDataForFigure3/3C/Figure3C_Blots.pdf]

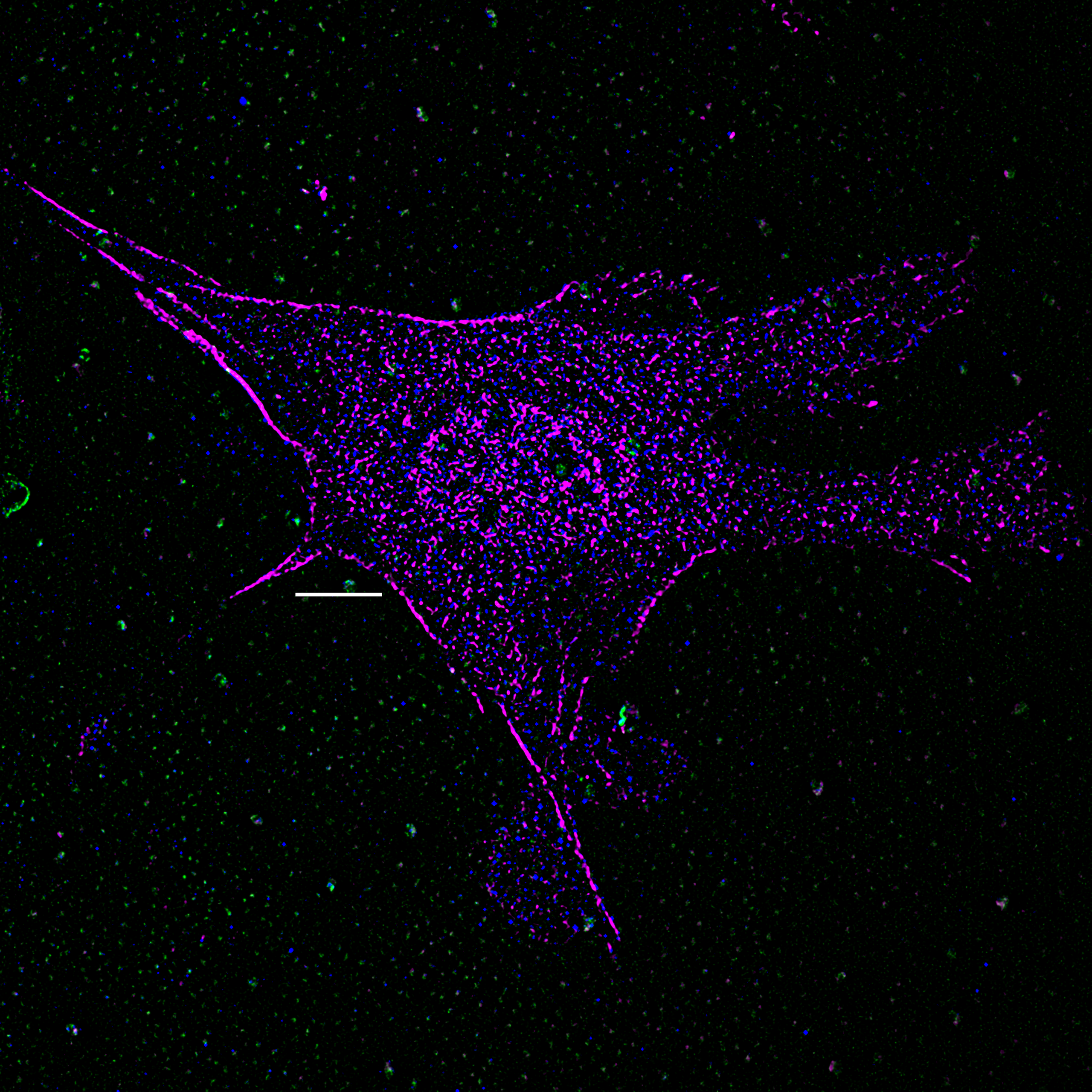

Supplement: Supplementary file 8 — Source data Fig. 4 [file 44319_2025_585_MOESM8_ESM.zip › EMBOR202561827V2_SourceDataForFigure4/4A/Figure4A_SIM┬▓Image_GFP-vector_Merge.tif]

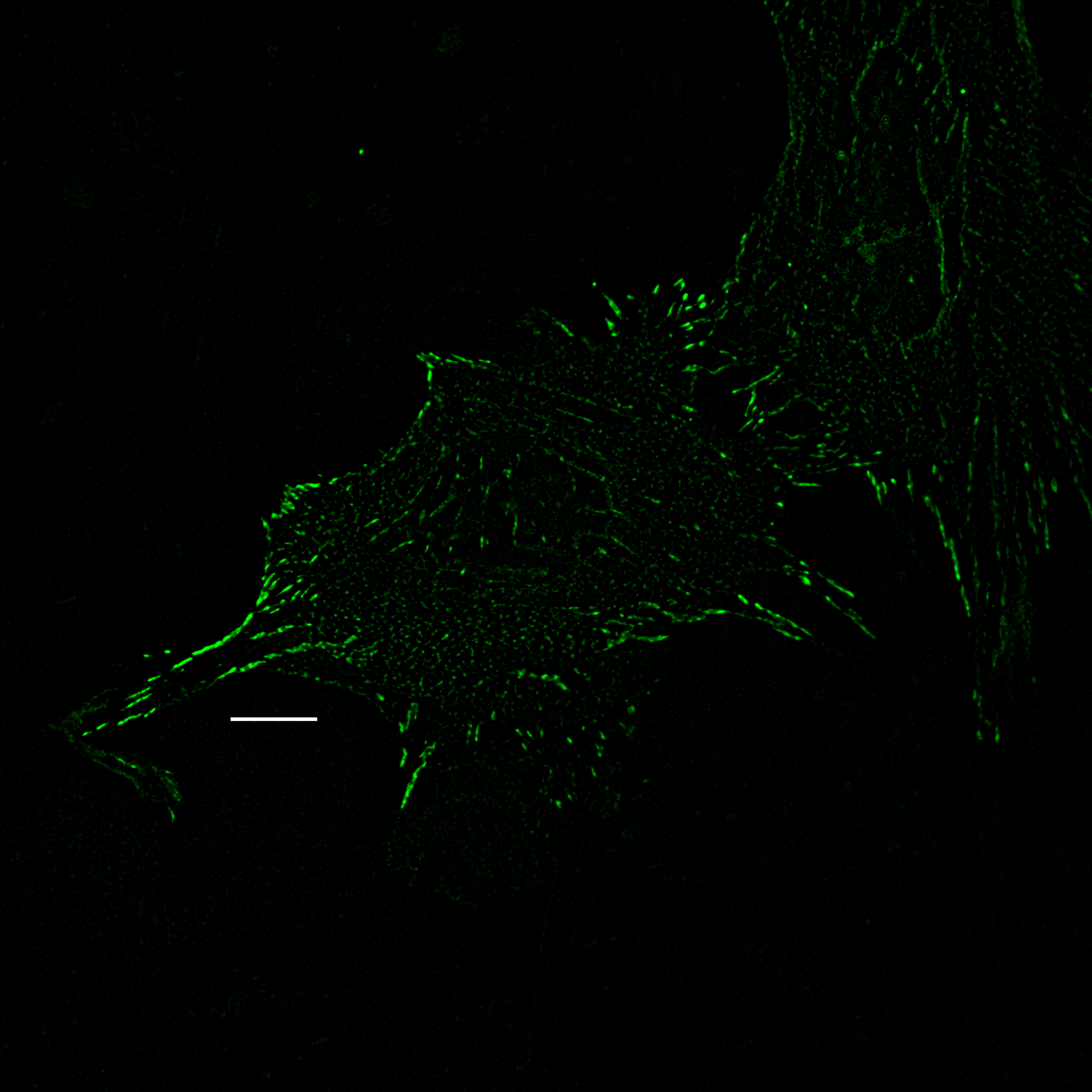

Supplement: Supplementary file 8 — Source data Fig. 4 [file 44319_2025_585_MOESM8_ESM.zip › EMBOR202561827V2_SourceDataForFigure4/4A/Figure4A_SIM┬▓Image_GFP-zyxin_GFP_GFP.tif]

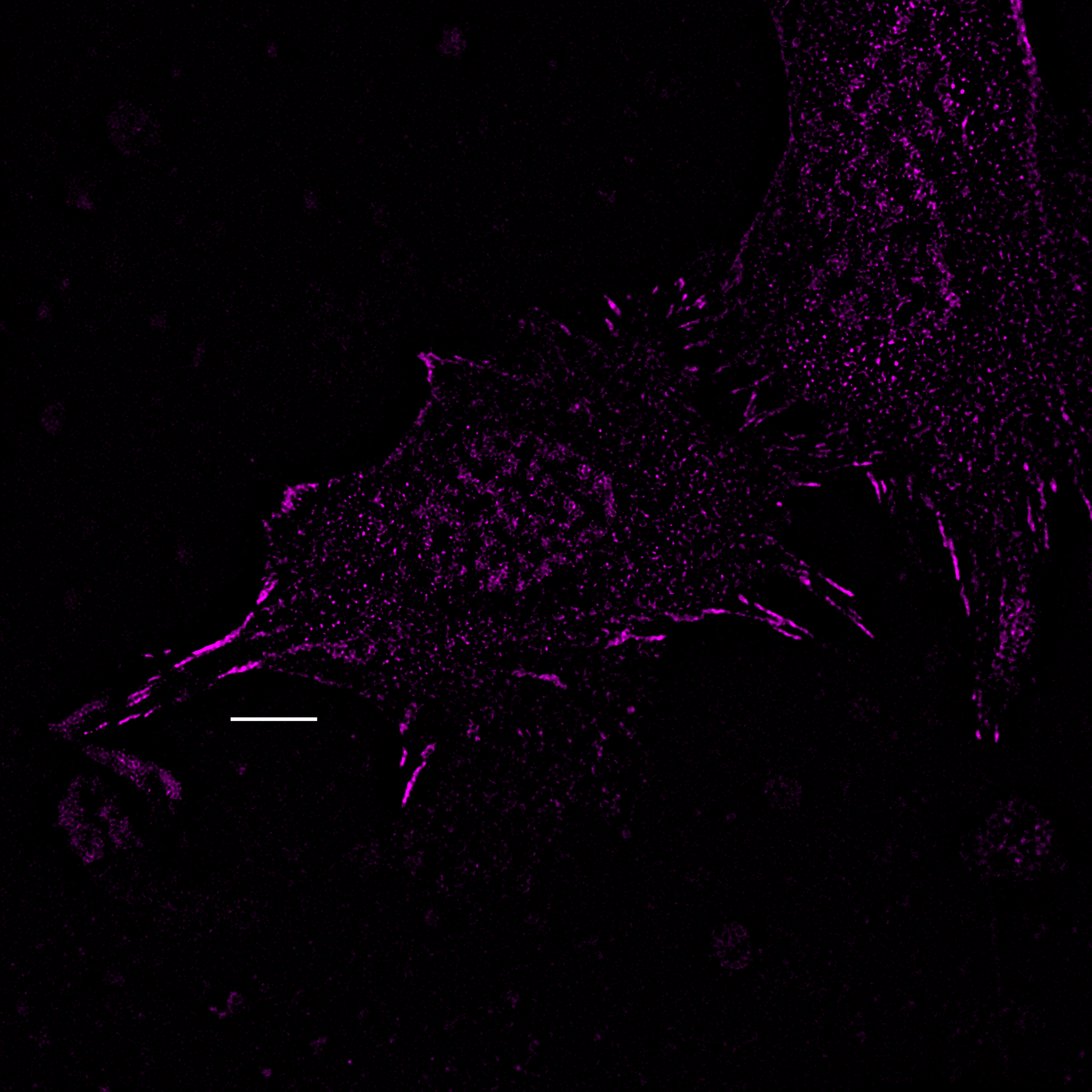

Supplement: Supplementary file 8 — Source data Fig. 4 [file 44319_2025_585_MOESM8_ESM.zip › EMBOR202561827V2_SourceDataForFigure4/4A/Figure4A_SIM┬▓Image_GFP-zyxin_UBE2O_AlexaFluor647.tif]

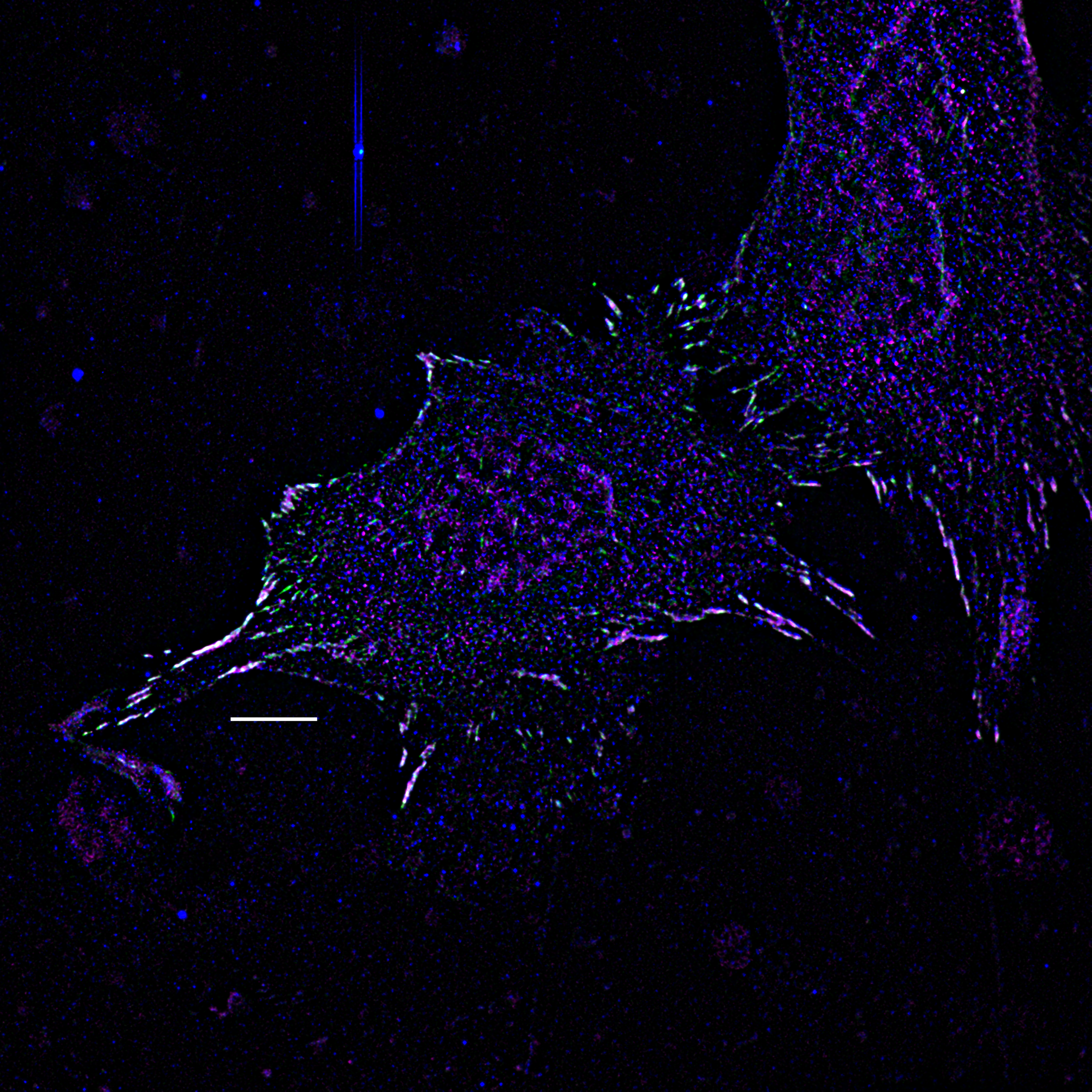

Supplement: Supplementary file 8 — Source data Fig. 4 [file 44319_2025_585_MOESM8_ESM.zip › EMBOR202561827V2_SourceDataForFigure4/4A/Figure4A_SIM┬▓Image_GFP-zyxin_Merge.tif]

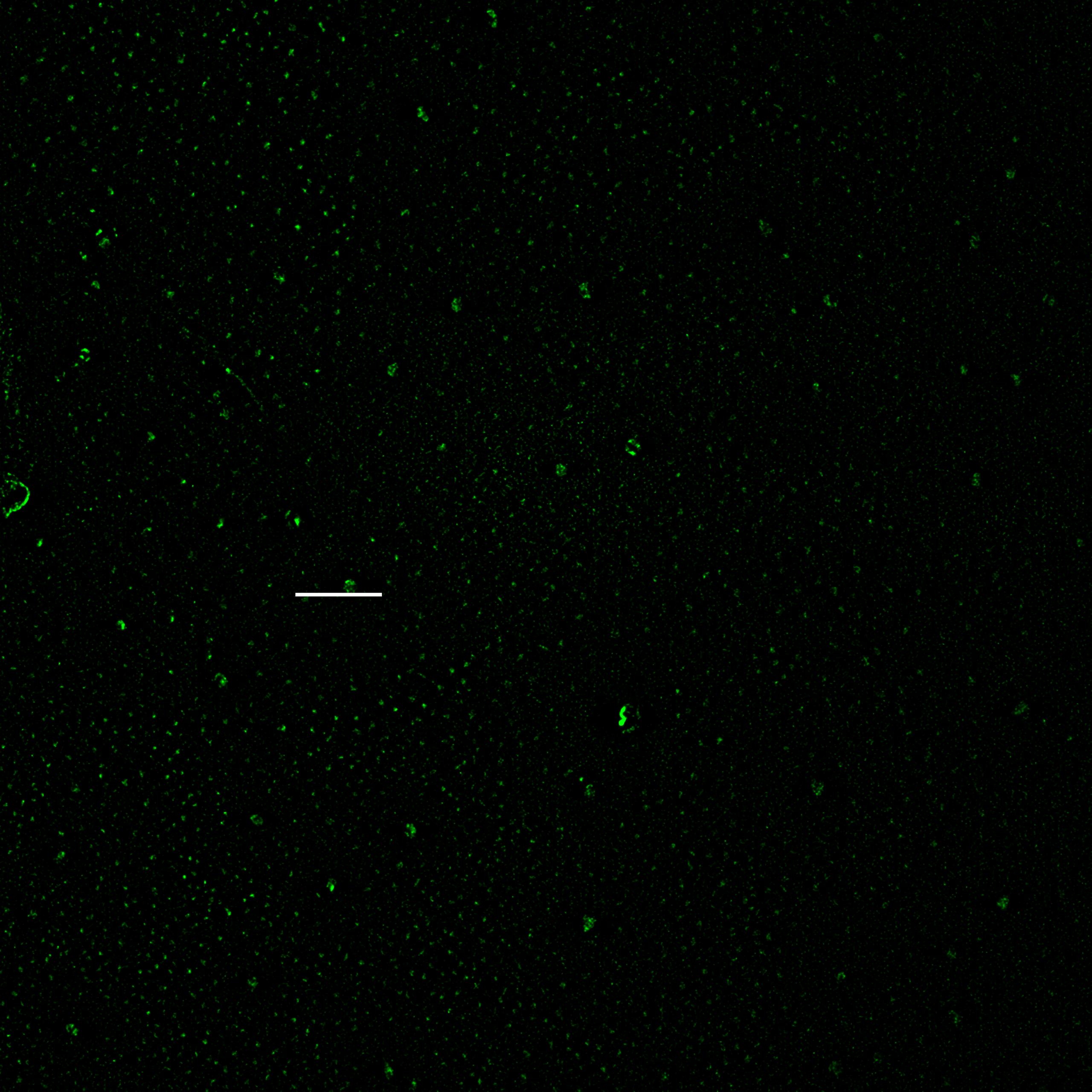

Supplement: Supplementary file 8 — Source data Fig. 4 [file 44319_2025_585_MOESM8_ESM.zip › EMBOR202561827V2_SourceDataForFigure4/4A/Figure4A_SIM┬▓Image_GFP-vector_GFP_GFP.tif]

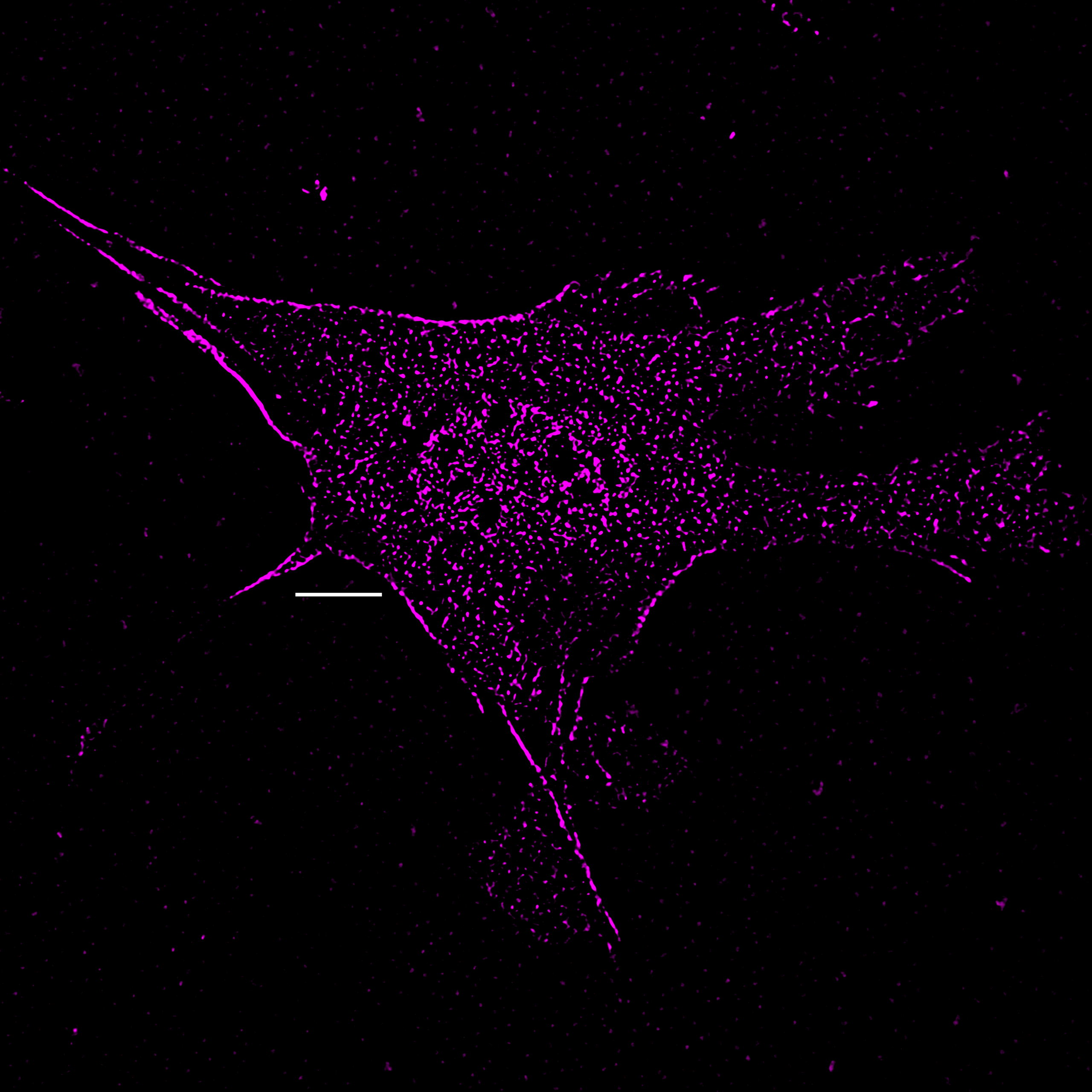

Supplement: Supplementary file 8 — Source data Fig. 4 [file 44319_2025_585_MOESM8_ESM.zip › EMBOR202561827V2_SourceDataForFigure4/4A/Figure4A_SIM┬▓Image_GFP-vector_UBE2O_AlexaFluor647.tif]

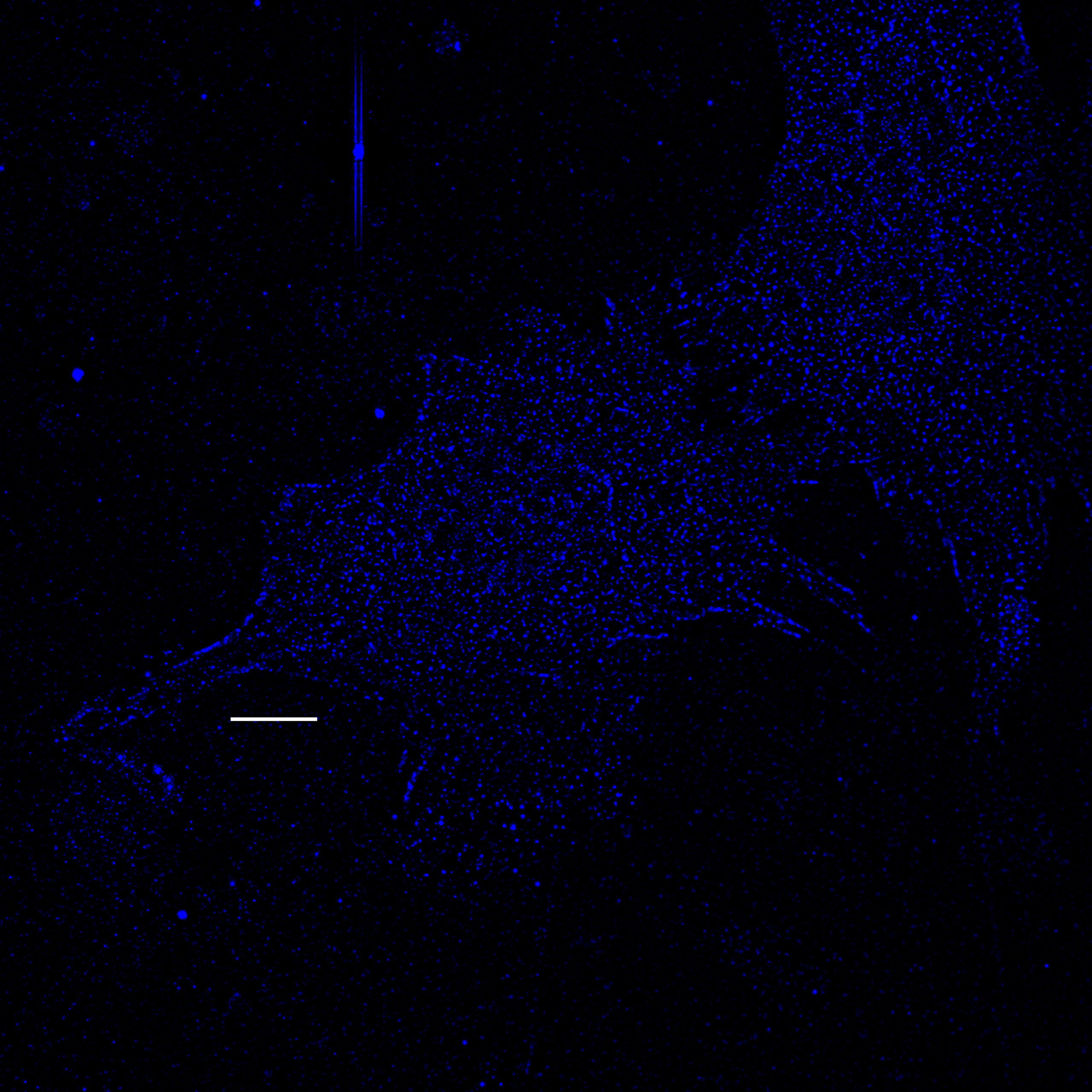

Supplement: Supplementary file 8 — Source data Fig. 4 [file 44319_2025_585_MOESM8_ESM.zip › EMBOR202561827V2_SourceDataForFigure4/4A/Figure4A_SIM┬▓Image_GFP-zyxin_CTNNA1_AlexaFluor568.tif]

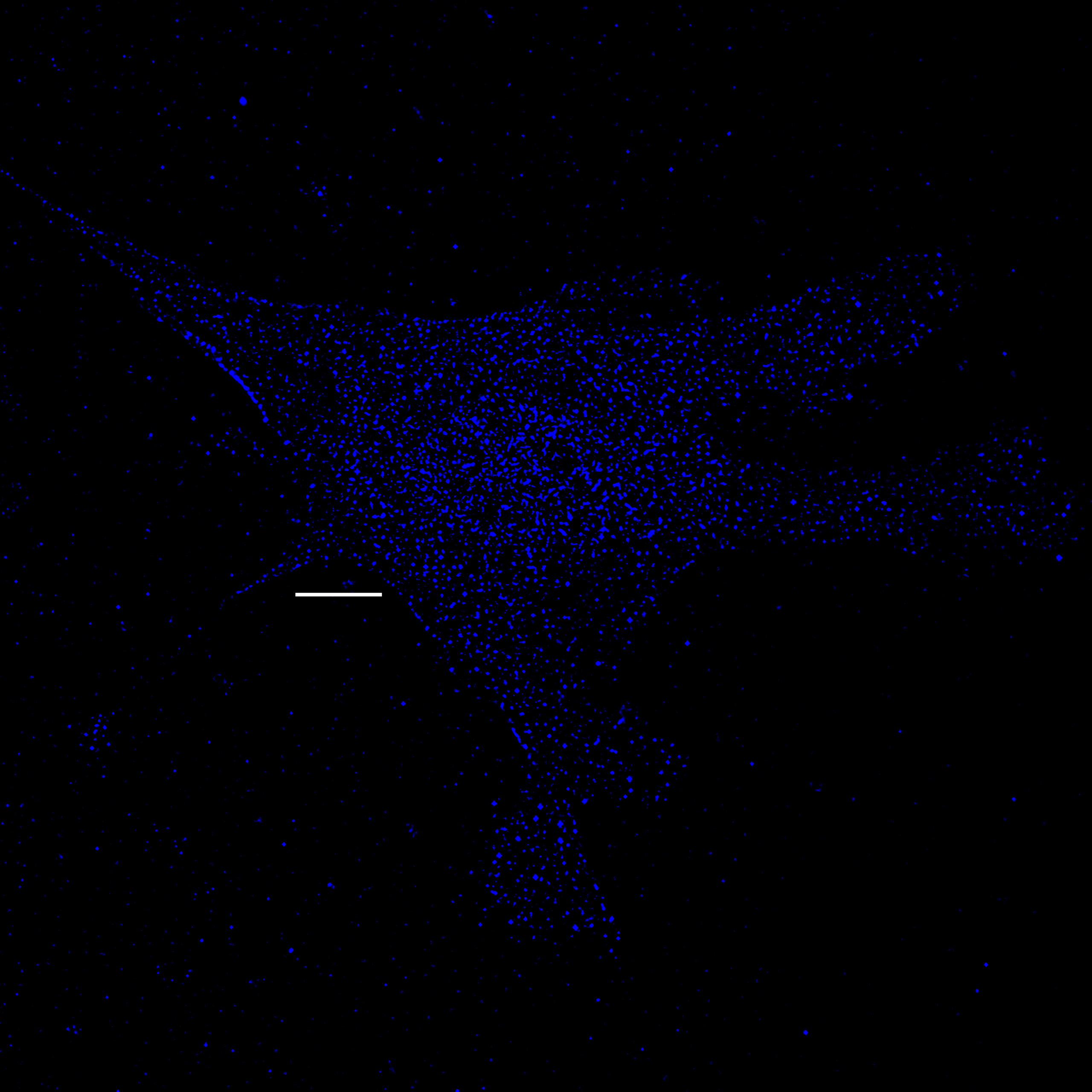

Supplement: Supplementary file 8 — Source data Fig. 4 [file 44319_2025_585_MOESM8_ESM.zip › EMBOR202561827V2_SourceDataForFigure4/4A/Figure4A_SIM┬▓Image_GFP-vector_CTNNA1_AlexaFluor568.tif]

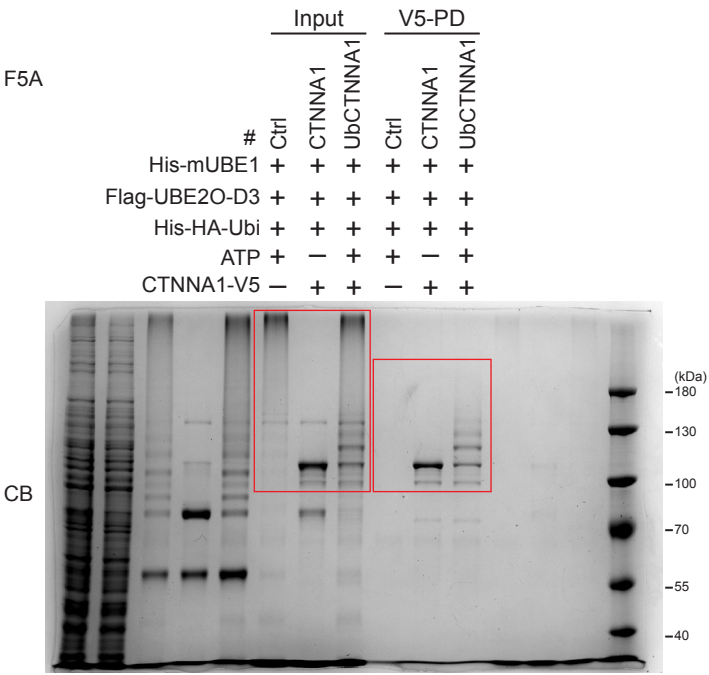

Supplement: Supplementary file 9 — Source data Fig. 5 [file 44319_2025_585_MOESM9_ESM.zip › EMBOR202561827V2_SourceDataForFigure5/5A/Figure5A_Blots.pdf]

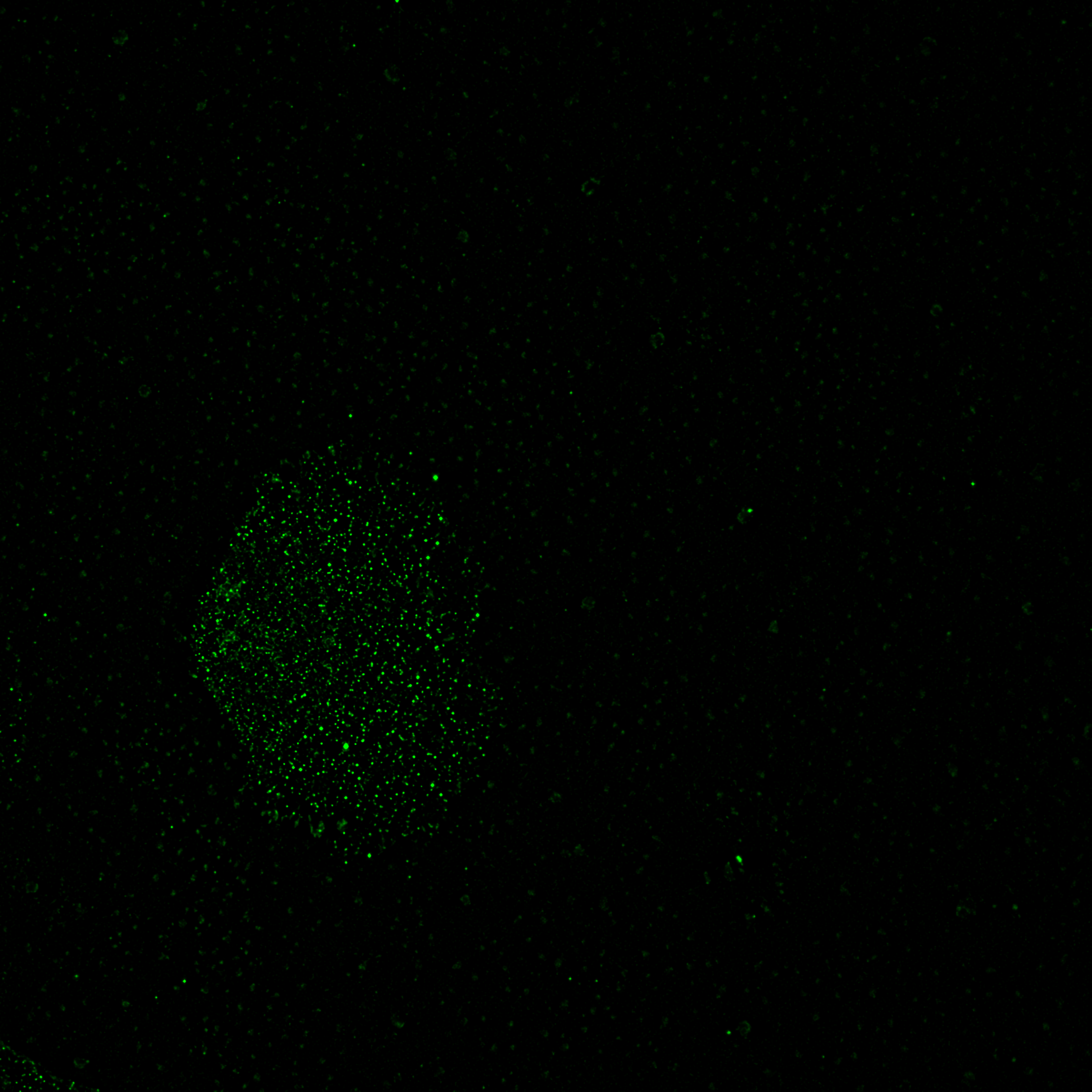

Supplement: Supplementary file 9 — Source data Fig. 5 [file 44319_2025_585_MOESM9_ESM.zip › EMBOR202561827V2_SourceDataForFigure5/5F/Figure5F_SIM┬▓Image_shUbe2o_CTNNA1_AlexaFluor488.tif]

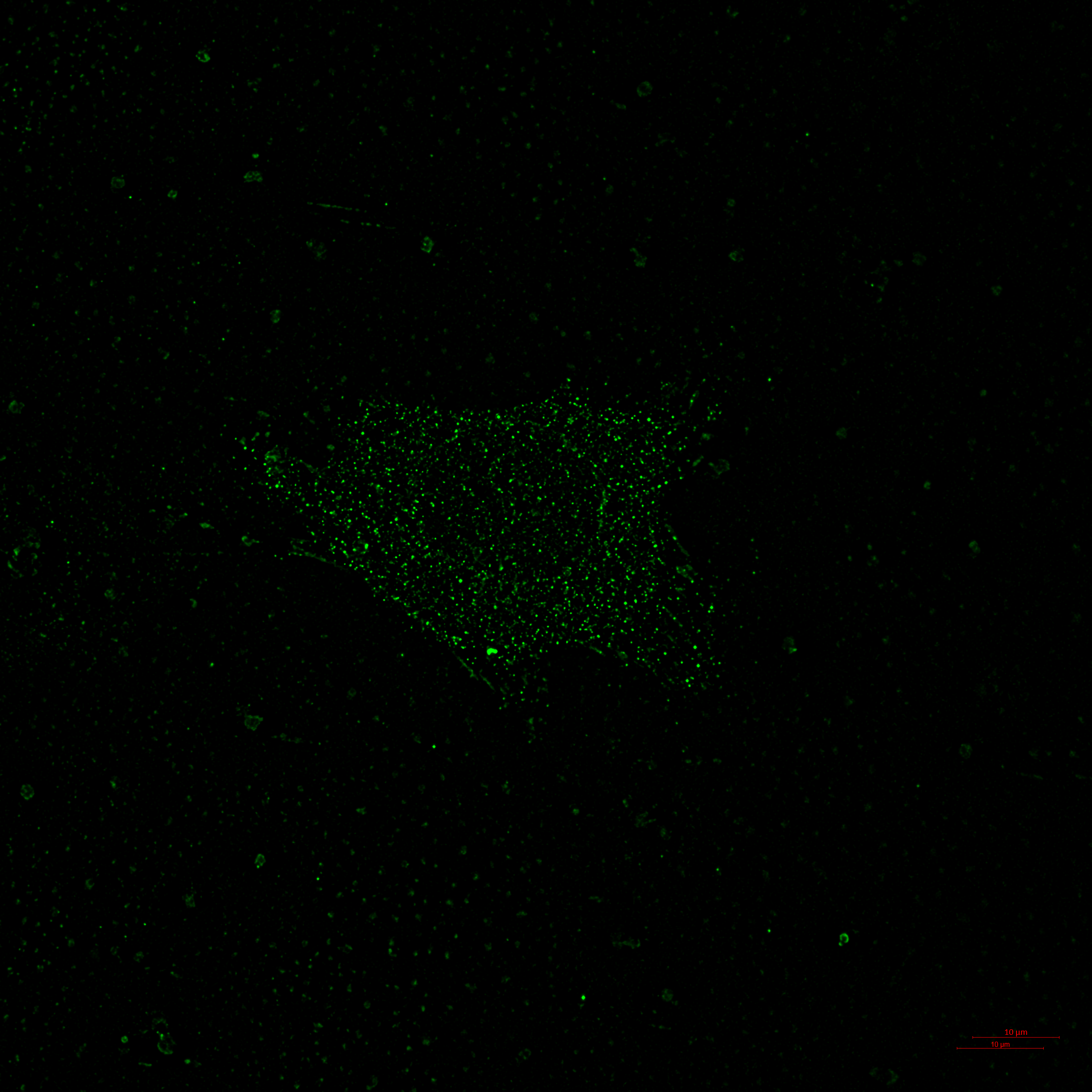

Supplement: Supplementary file 9 — Source data Fig. 5 [file 44319_2025_585_MOESM9_ESM.zip › EMBOR202561827V2_SourceDataForFigure5/5F/Figure5F_SIM┬▓Image_Res_UBE2O-CS_CTNNA1_AlexaFluor488.tif]

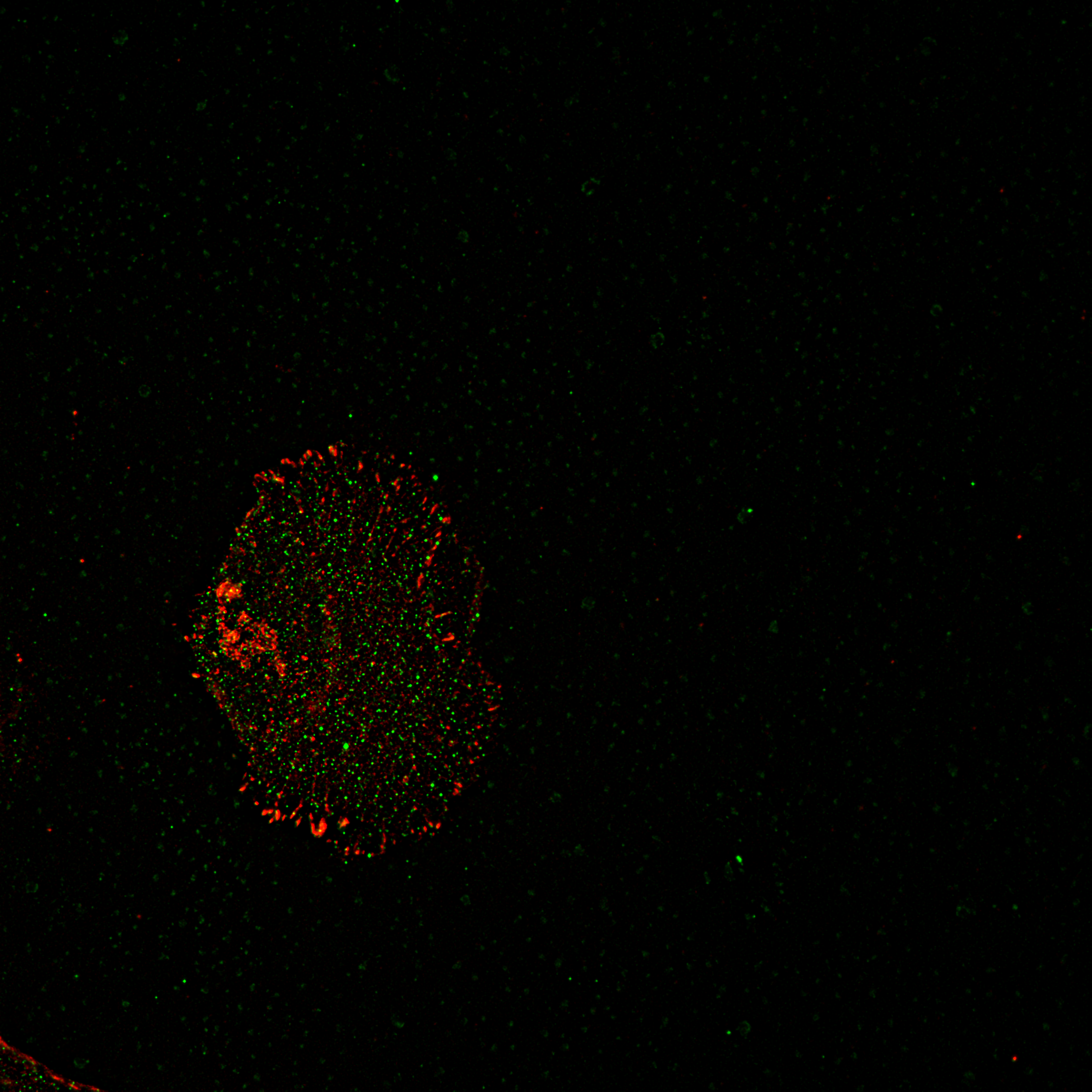

Supplement: Supplementary file 9 — Source data Fig. 5 [file 44319_2025_585_MOESM9_ESM.zip › EMBOR202561827V2_SourceDataForFigure5/5F/Figure5F_SIM┬▓Image_shUbe2o_Merge.tif]

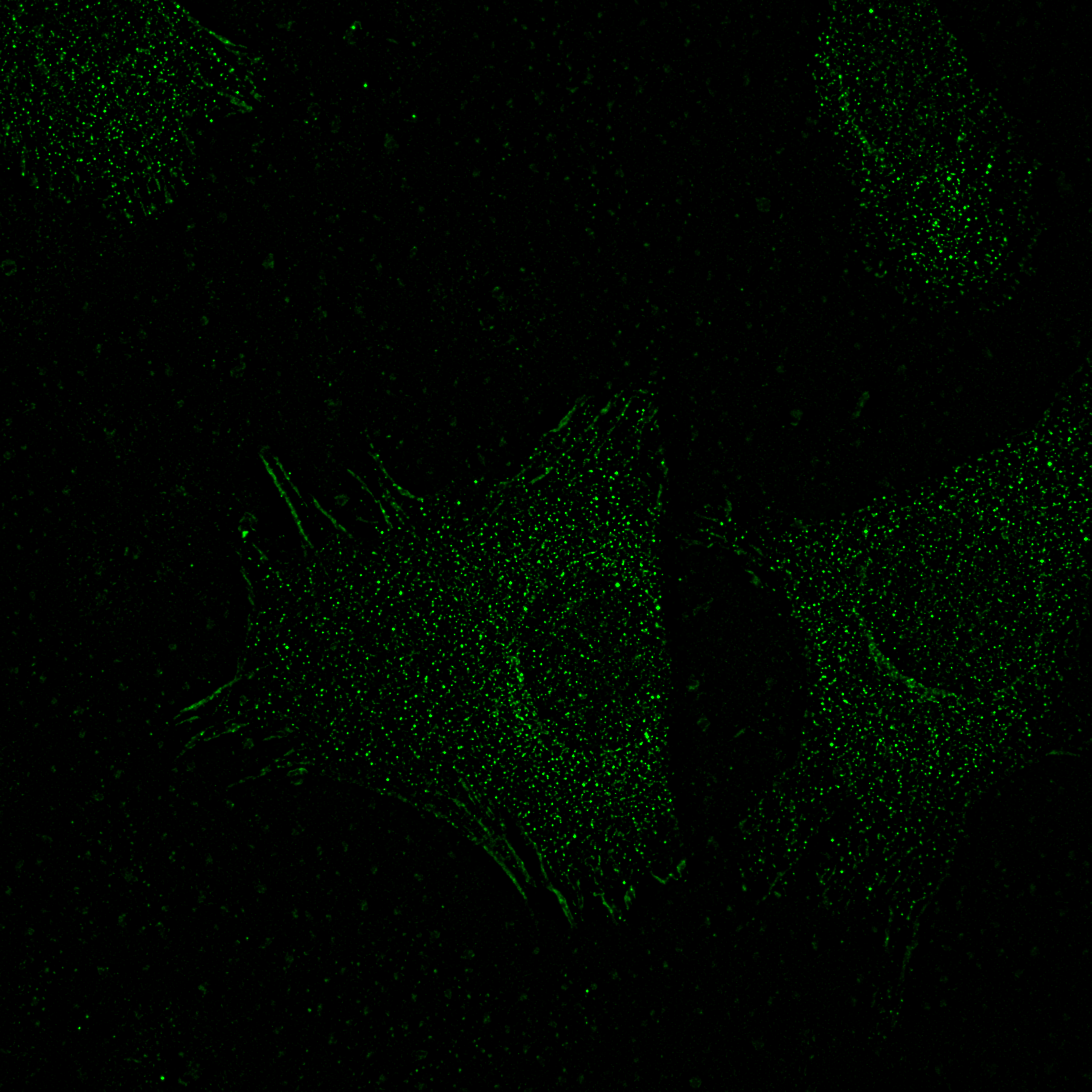

Supplement: Supplementary file 9 — Source data Fig. 5 [file 44319_2025_585_MOESM9_ESM.zip › EMBOR202561827V2_SourceDataForFigure5/5F/Figure5F_SIM┬▓Image_shLuc_CTNNA1_AlexaFluor488.tif]

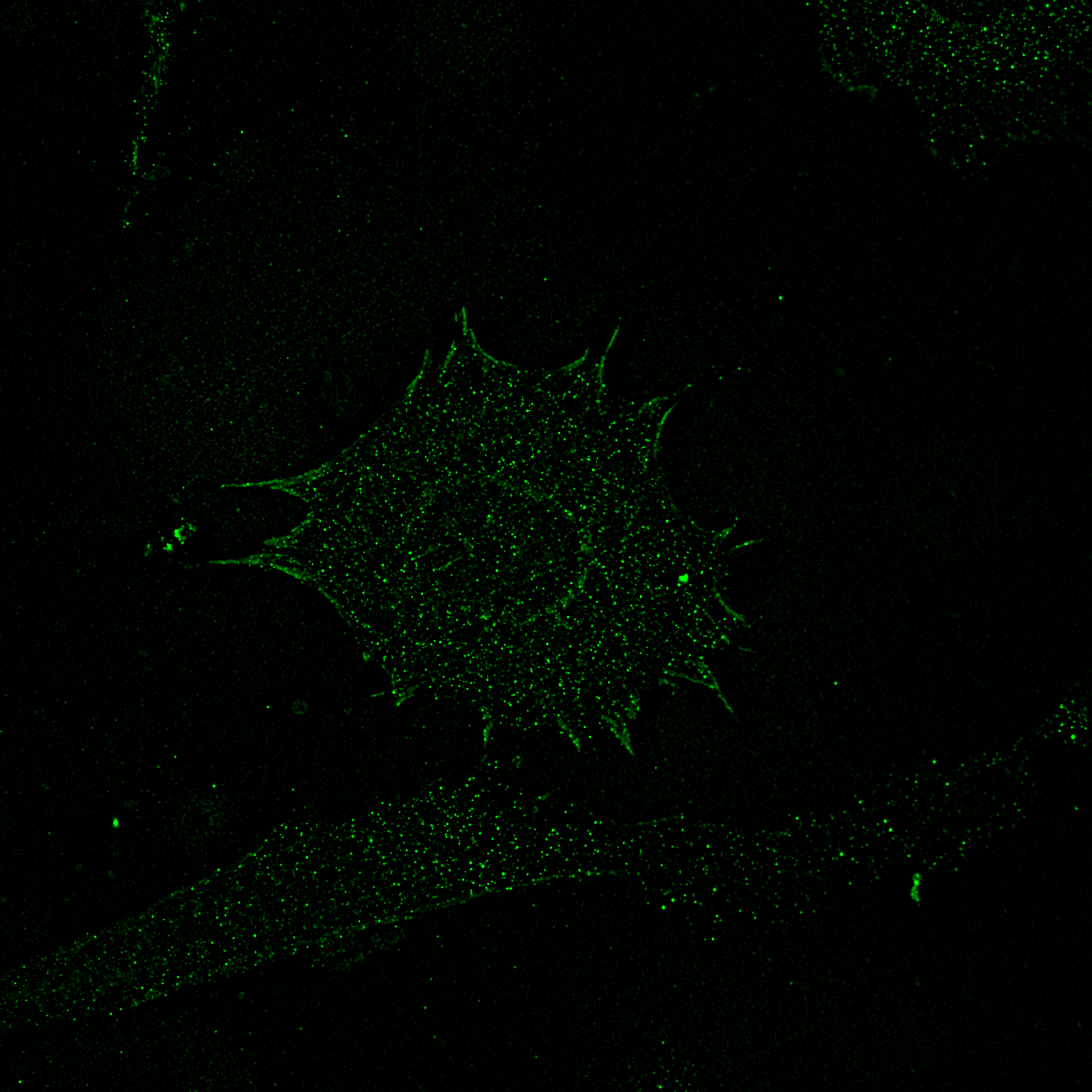

Supplement: Supplementary file 9 — Source data Fig. 5 [file 44319_2025_585_MOESM9_ESM.zip › EMBOR202561827V2_SourceDataForFigure5/5F/Figure5F_SIM┬▓Image_Res_UBE2O-WT_CTNNA1_AlexaFluor488.tif]

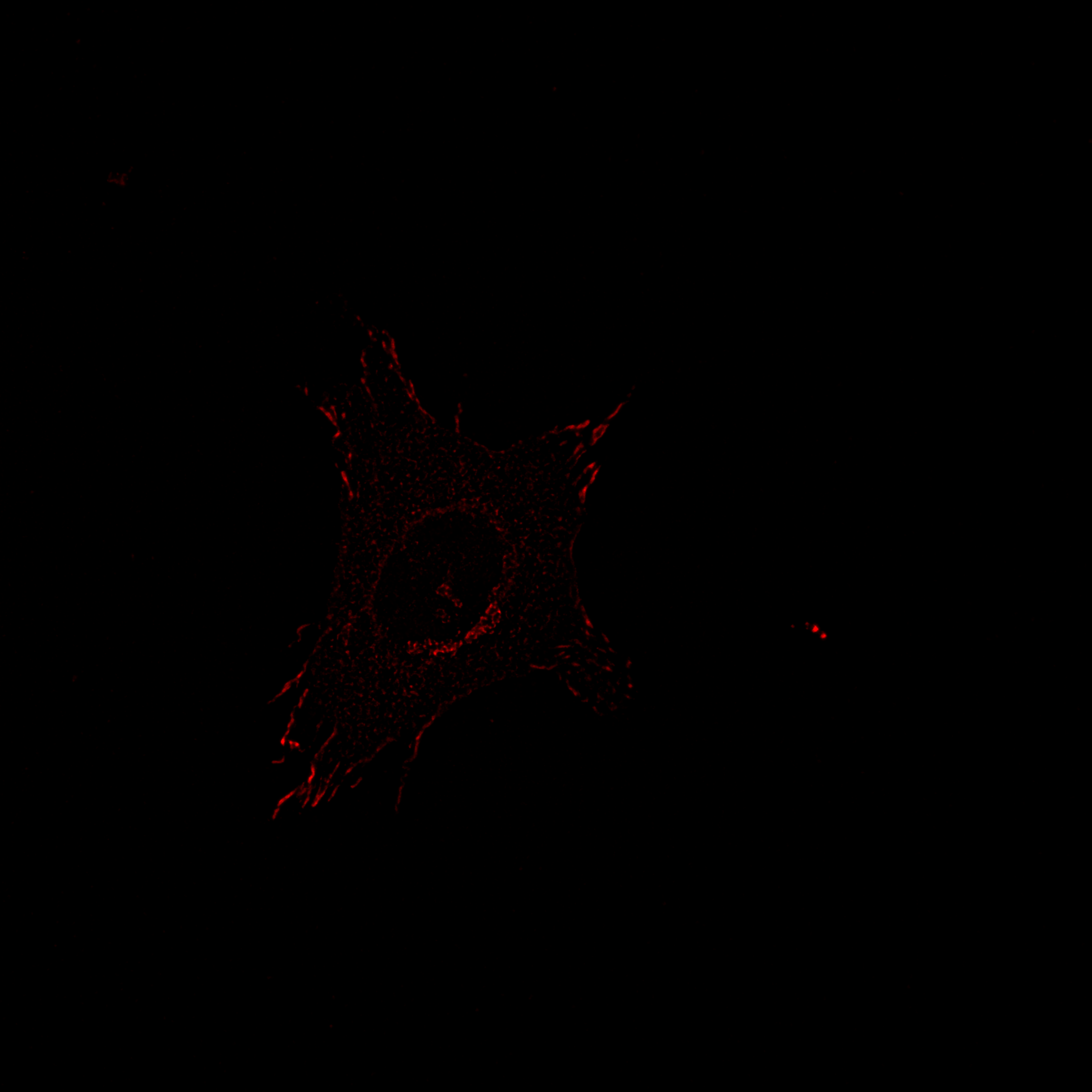

Supplement: Supplementary file 9 — Source data Fig. 5 [file 44319_2025_585_MOESM9_ESM.zip › EMBOR202561827V2_SourceDataForFigure5/5F/Figure5F_SIM┬▓Image_Res_Vector_Zyxin_AlexaFluor568.tif]

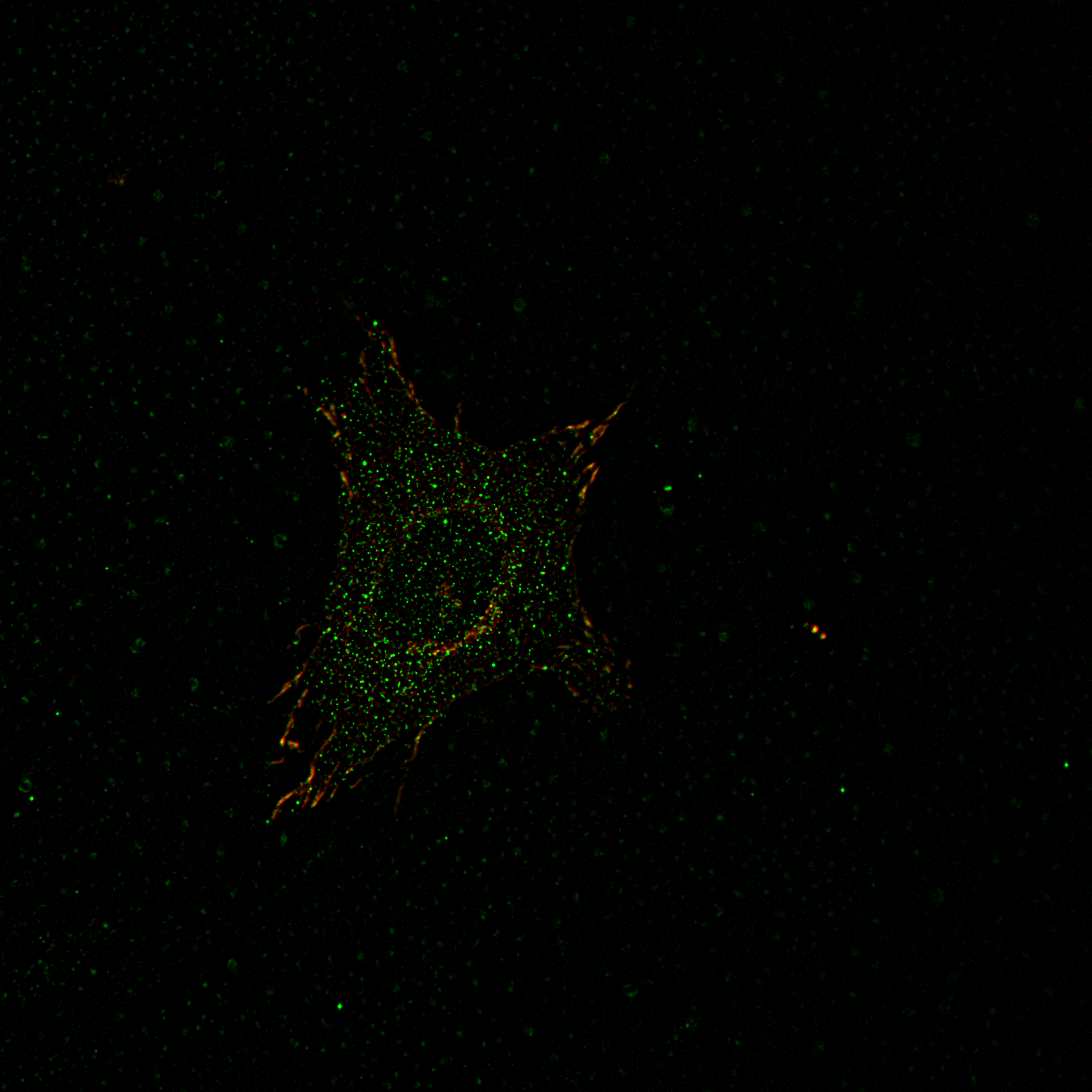

Supplement: Supplementary file 9 — Source data Fig. 5 [file 44319_2025_585_MOESM9_ESM.zip › EMBOR202561827V2_SourceDataForFigure5/5F/Figure5F_SIM┬▓Image_Res_Vector_Merge.tif]

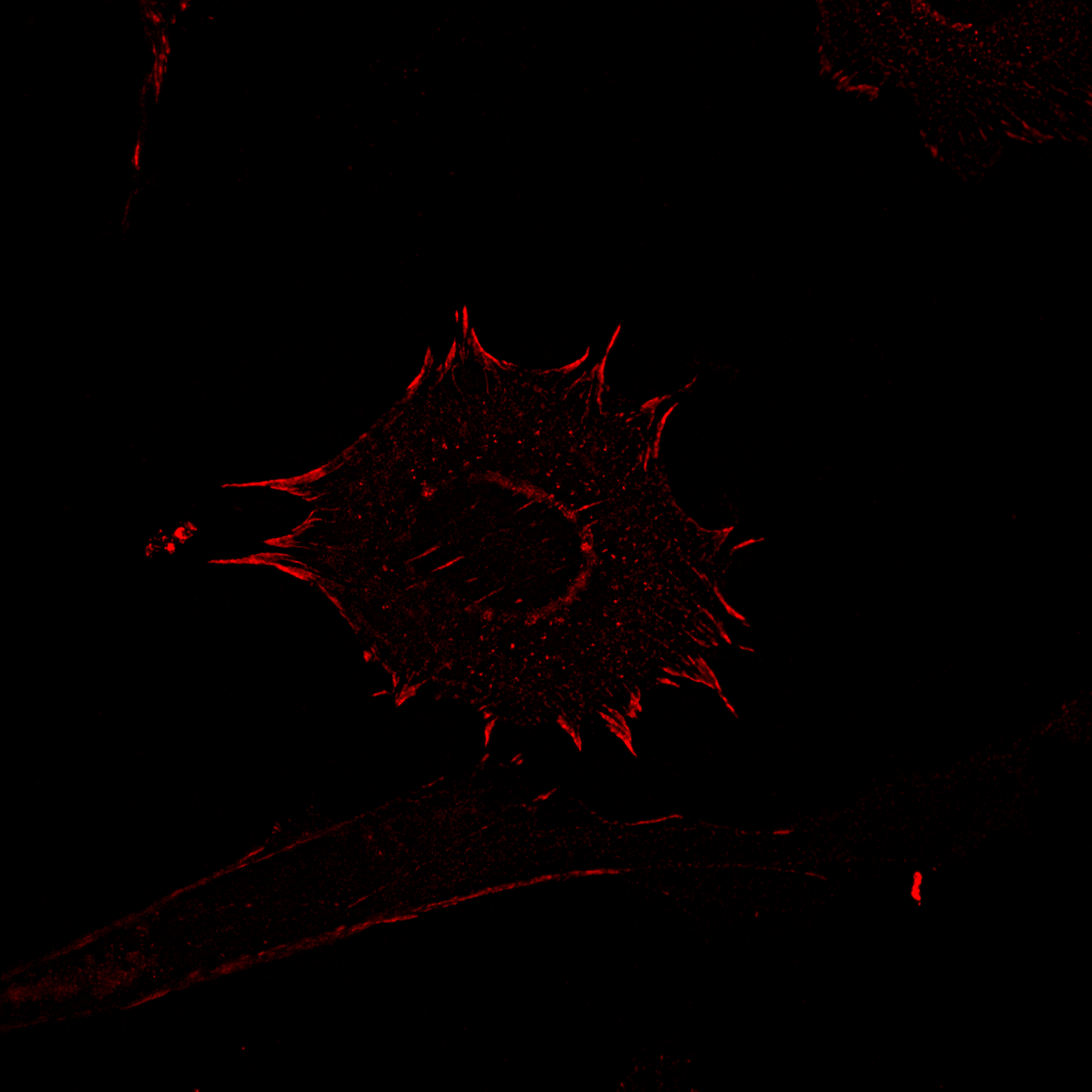

Supplement: Supplementary file 9 — Source data Fig. 5 [file 44319_2025_585_MOESM9_ESM.zip › EMBOR202561827V2_SourceDataForFigure5/5F/Figure5F_SIM┬▓Image_Res_UBE2O-WT_Zyxin_AlexaFluor568.tif]

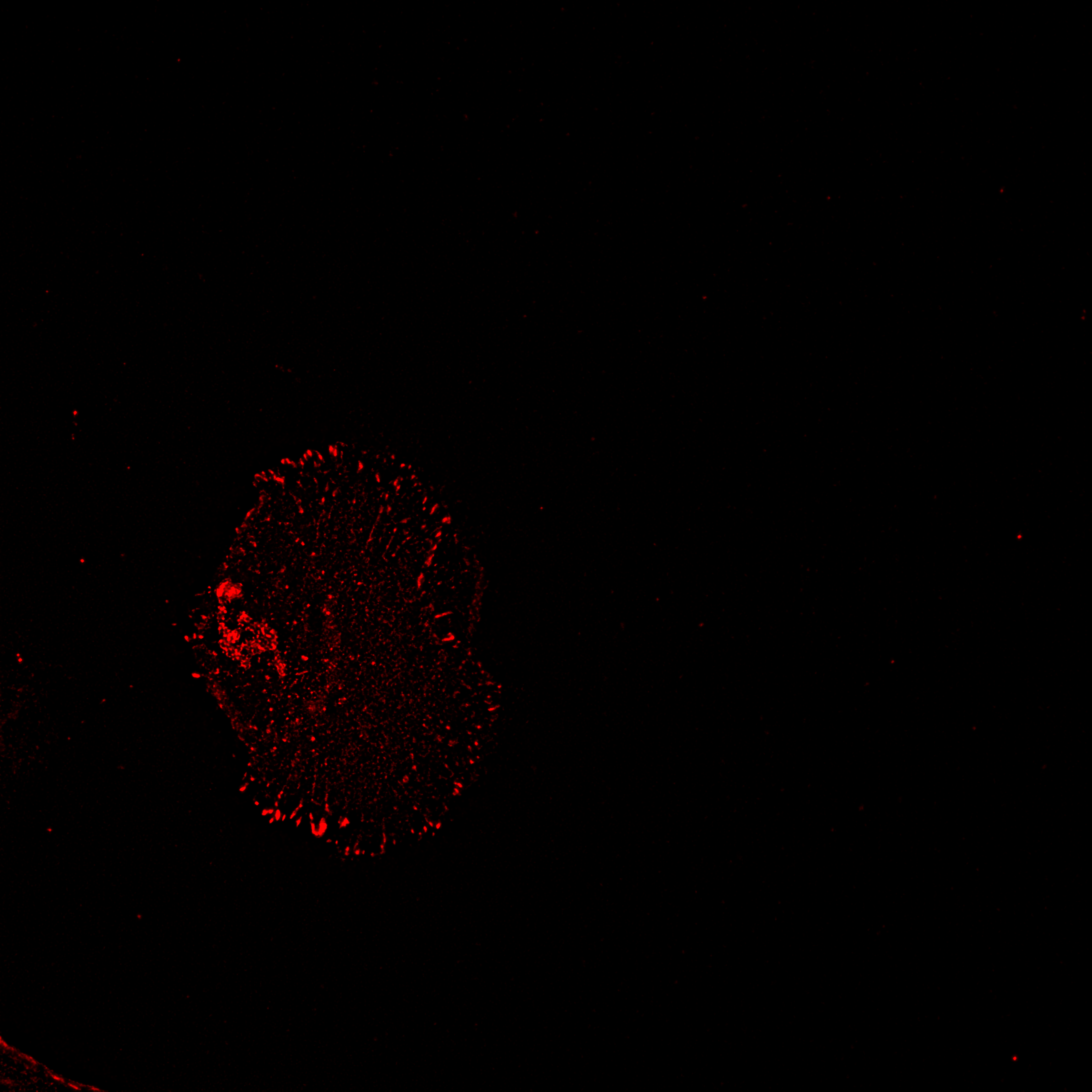

Supplement: Supplementary file 9 — Source data Fig. 5 [file 44319_2025_585_MOESM9_ESM.zip › EMBOR202561827V2_SourceDataForFigure5/5F/Figure5F_SIM┬▓Image_shUbe2o_Zyxin_AlexaFluor568.tif]

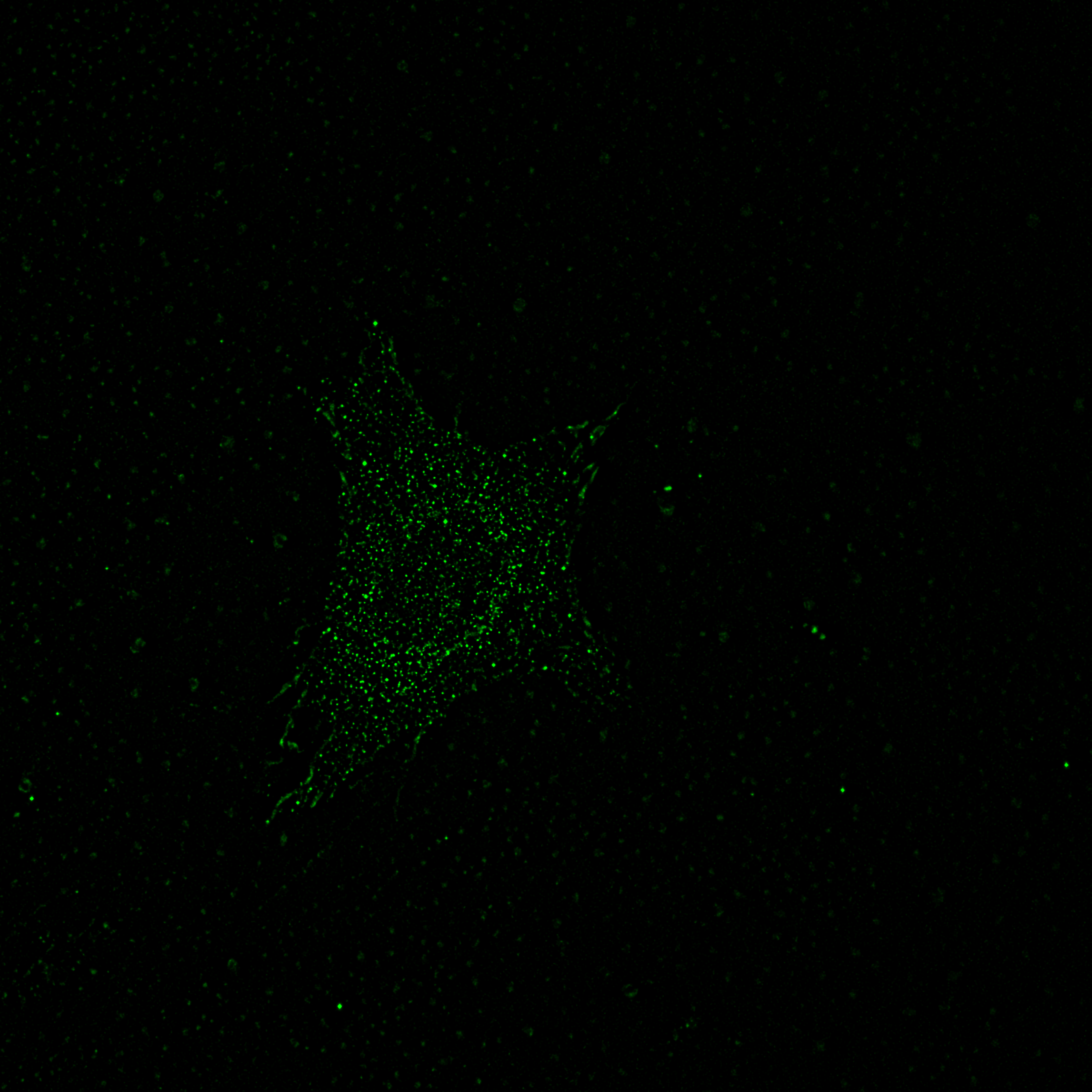

Supplement: Supplementary file 9 — Source data Fig. 5 [file 44319_2025_585_MOESM9_ESM.zip › EMBOR202561827V2_SourceDataForFigure5/5F/Figure5F_SIM┬▓Image_Res_Vector_CTNNA1_AlexaFluor488.tif]

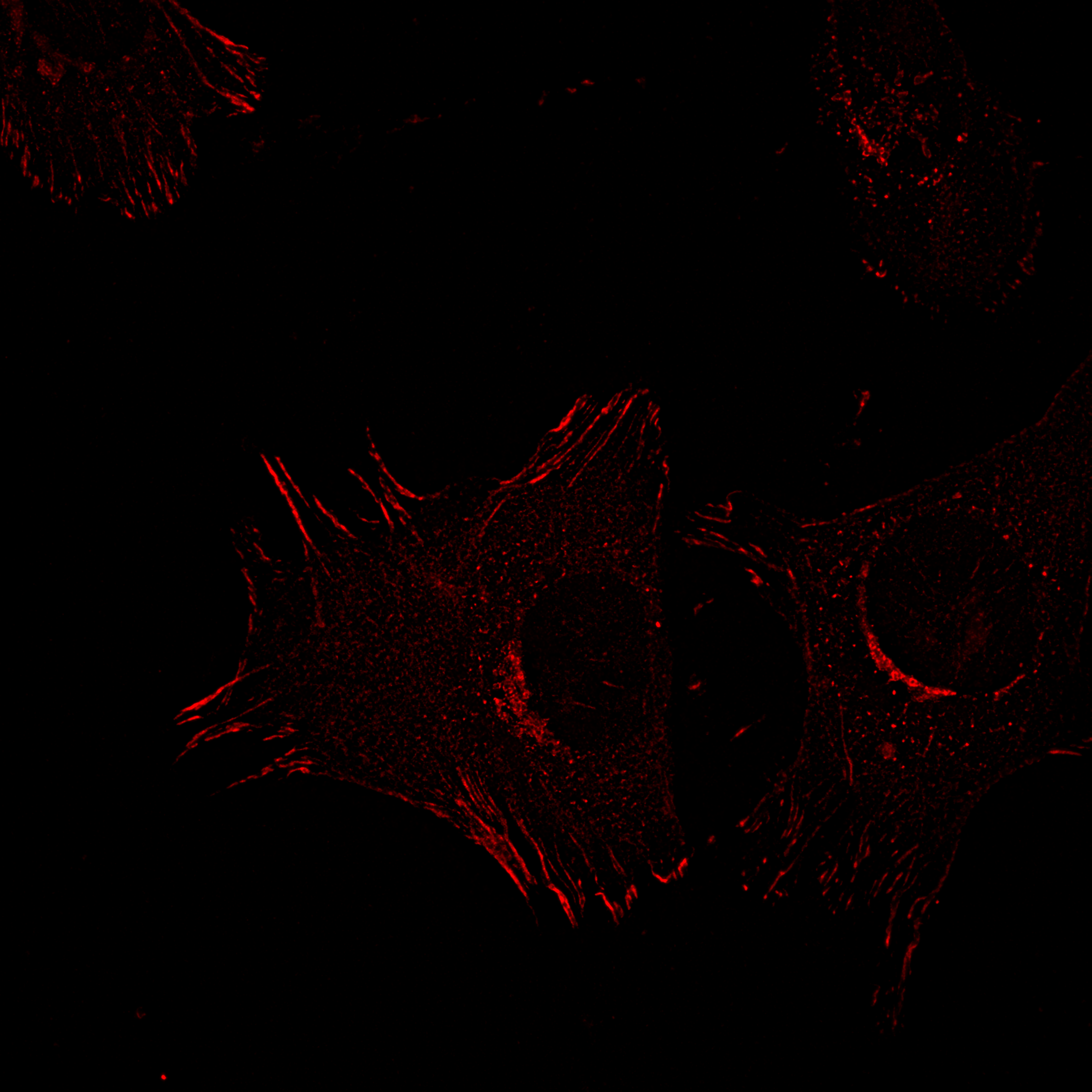

Supplement: Supplementary file 9 — Source data Fig. 5 [file 44319_2025_585_MOESM9_ESM.zip › EMBOR202561827V2_SourceDataForFigure5/5F/Figure5F_SIM┬▓Image_shLuc_Zyxin_AlexaFluor568.tif]

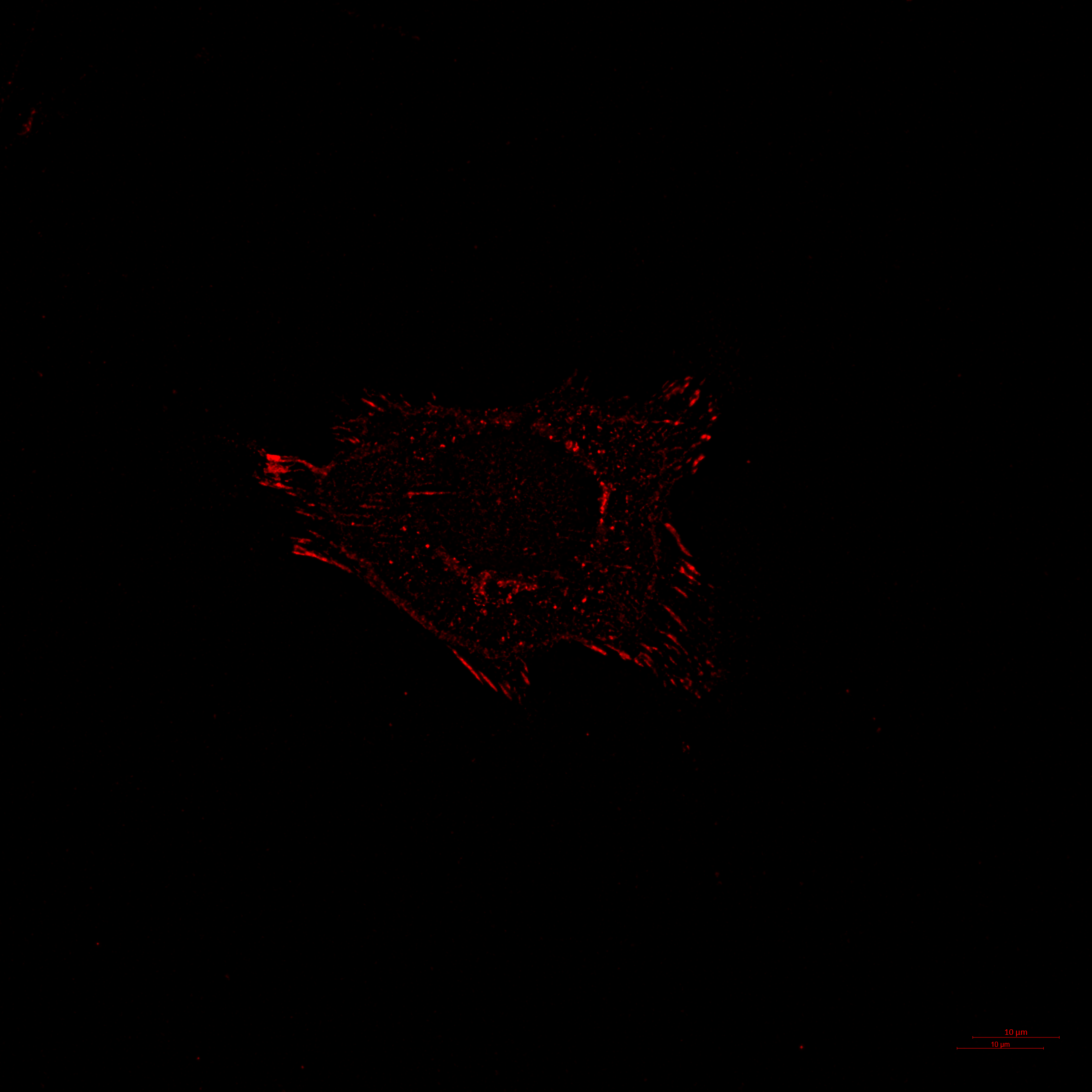

Supplement: Supplementary file 9 — Source data Fig. 5 [file 44319_2025_585_MOESM9_ESM.zip › EMBOR202561827V2_SourceDataForFigure5/5F/Figure5F_SIM┬▓Image_Res_UBE2O-CS_Zyxin_AlexaFluor568.tif]

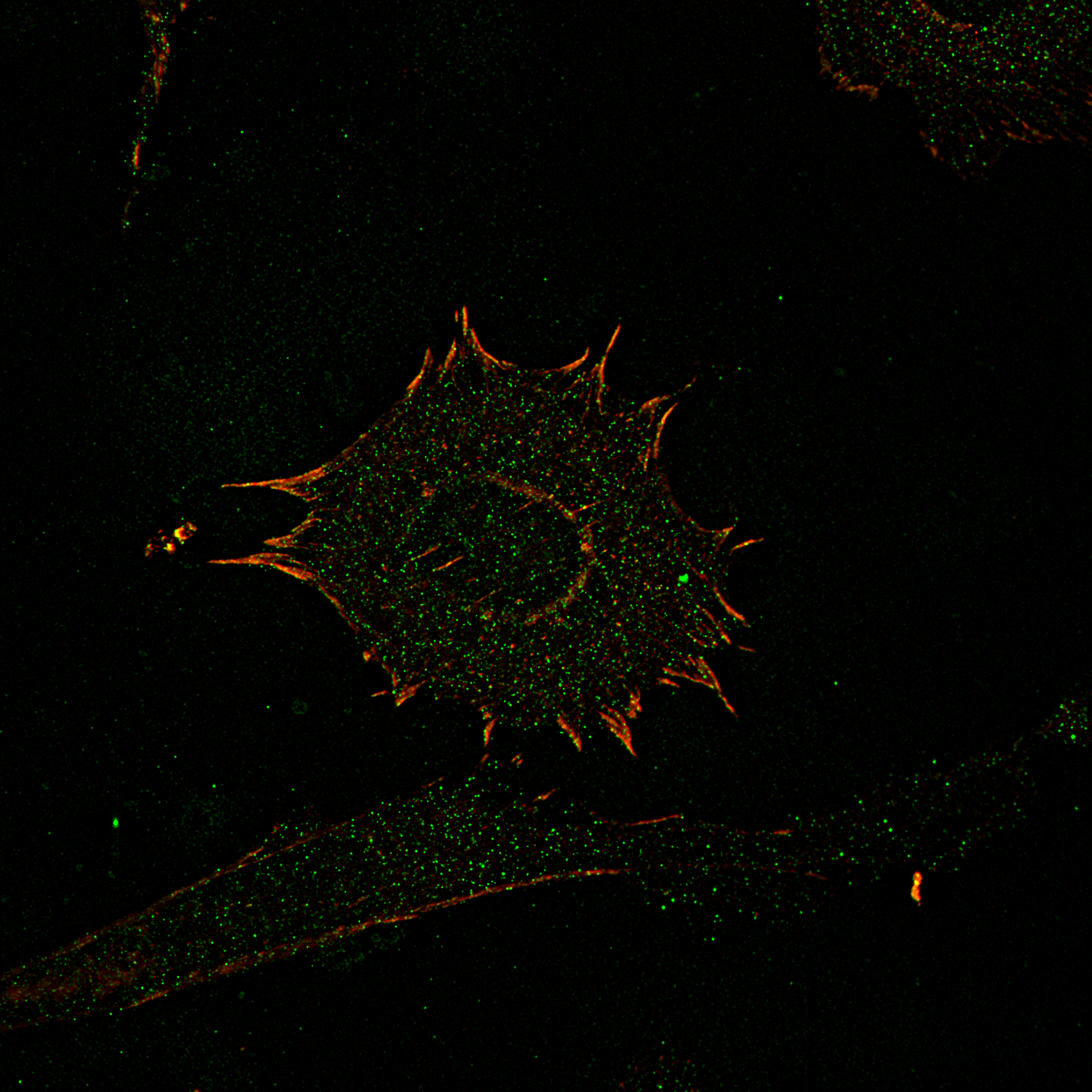

Supplement: Supplementary file 9 — Source data Fig. 5 [file 44319_2025_585_MOESM9_ESM.zip › EMBOR202561827V2_SourceDataForFigure5/5F/Figure5F_SIM┬▓Image_Res_UBE2O-WT_Merge.tif]

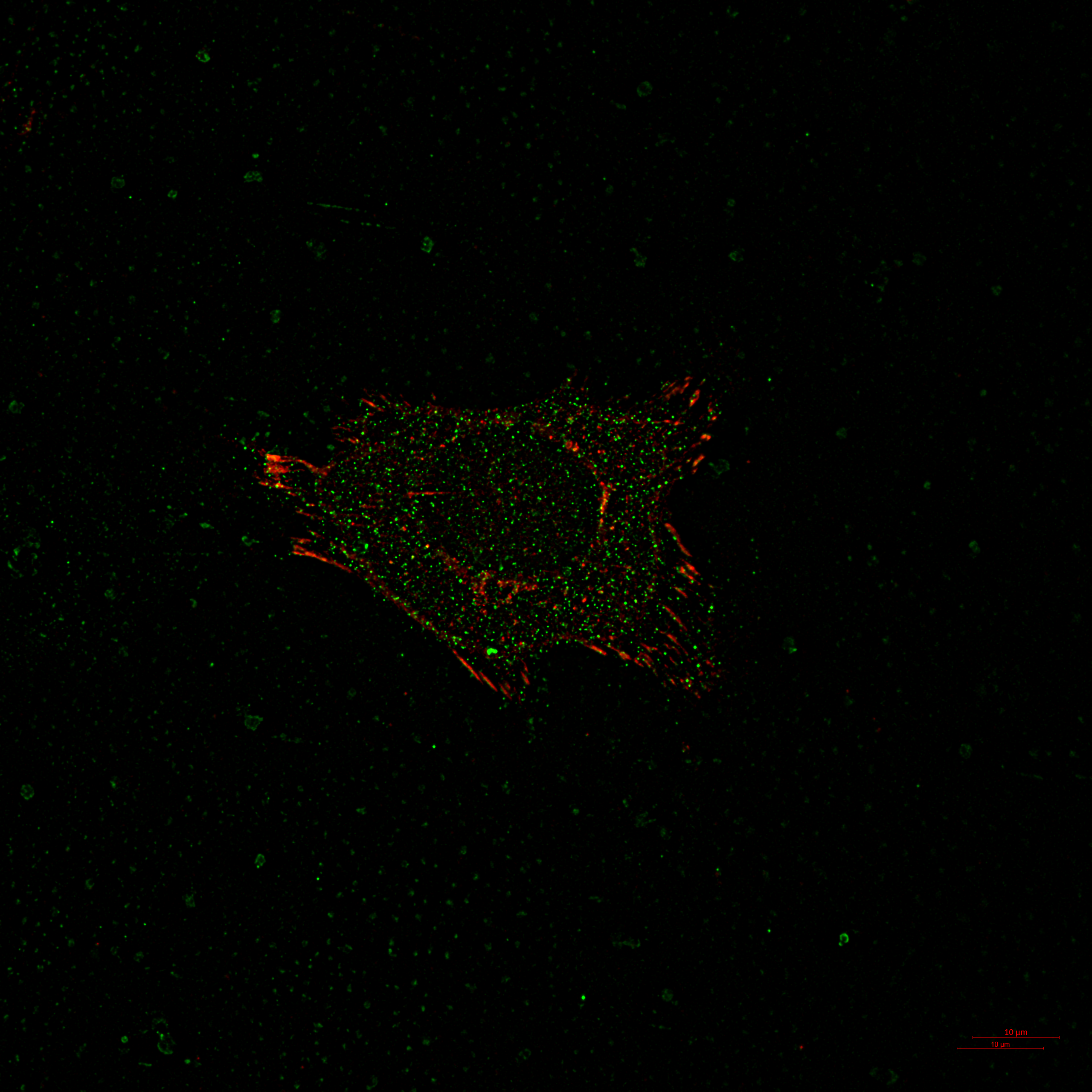

Supplement: Supplementary file 9 — Source data Fig. 5 [file 44319_2025_585_MOESM9_ESM.zip › EMBOR202561827V2_SourceDataForFigure5/5F/Figure5F_SIM┬▓Image_Res_UBE2O-CS_Merge.tif]

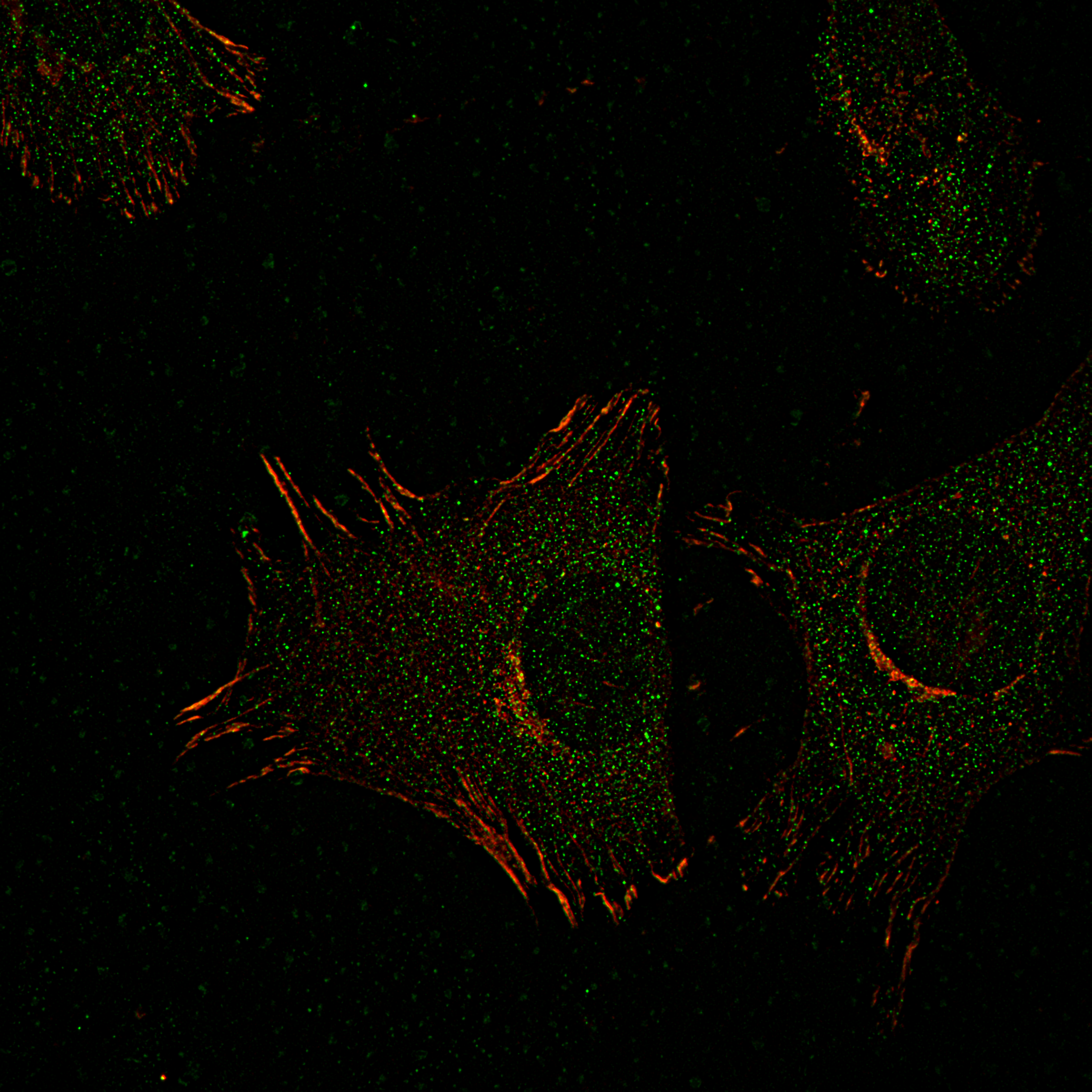

Supplement: Supplementary file 9 — Source data Fig. 5 [file 44319_2025_585_MOESM9_ESM.zip › EMBOR202561827V2_SourceDataForFigure5/5F/Figure5F_SIM┬▓Image_shLuc_Merge.tif]

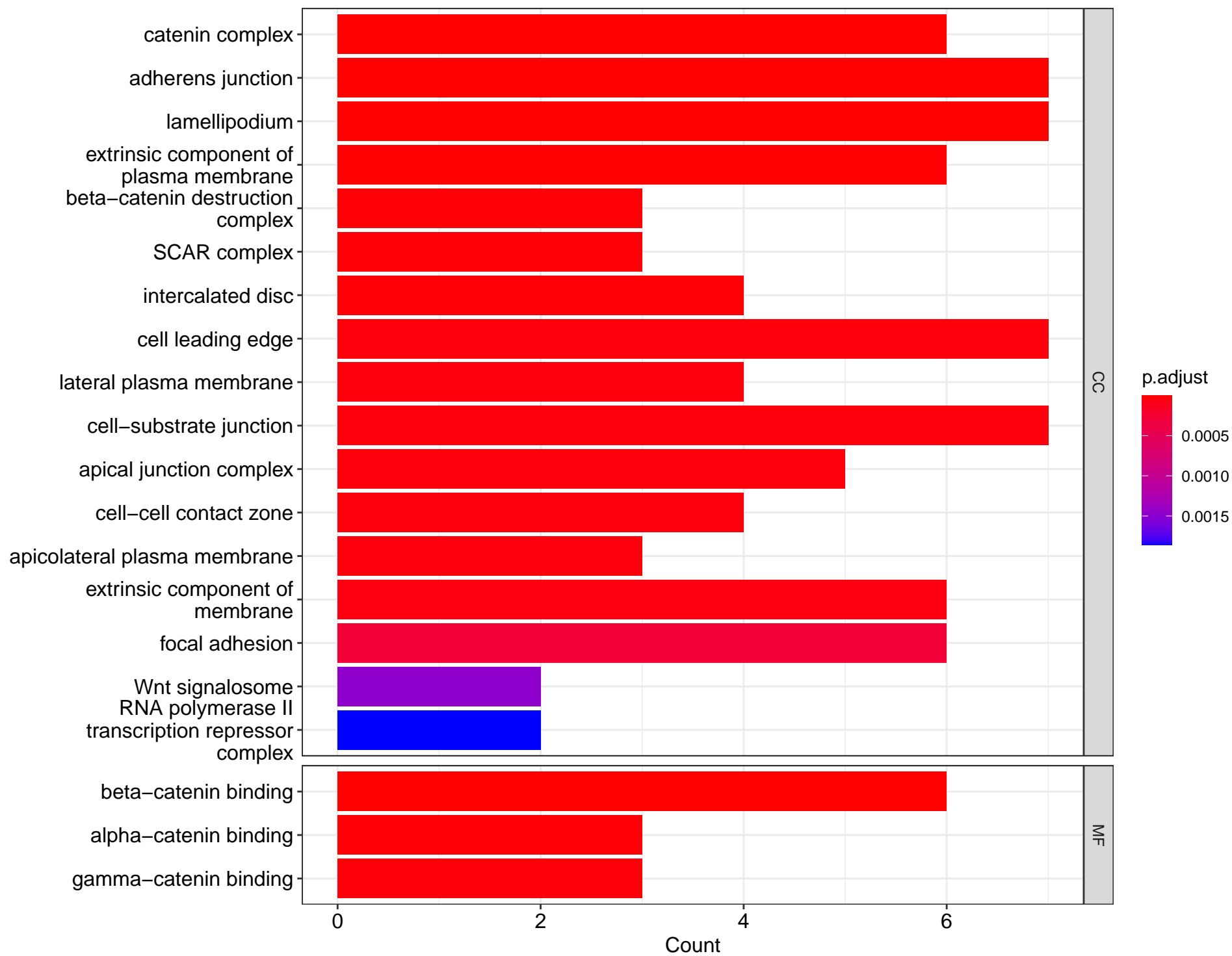

Supplement: Supplementary file 9 — Source data Fig. 5 [file 44319_2025_585_MOESM9_ESM.zip › EMBOR202561827V2_SourceDataForFigure5/5C/Figure5C_DEP2_GO.pdf]

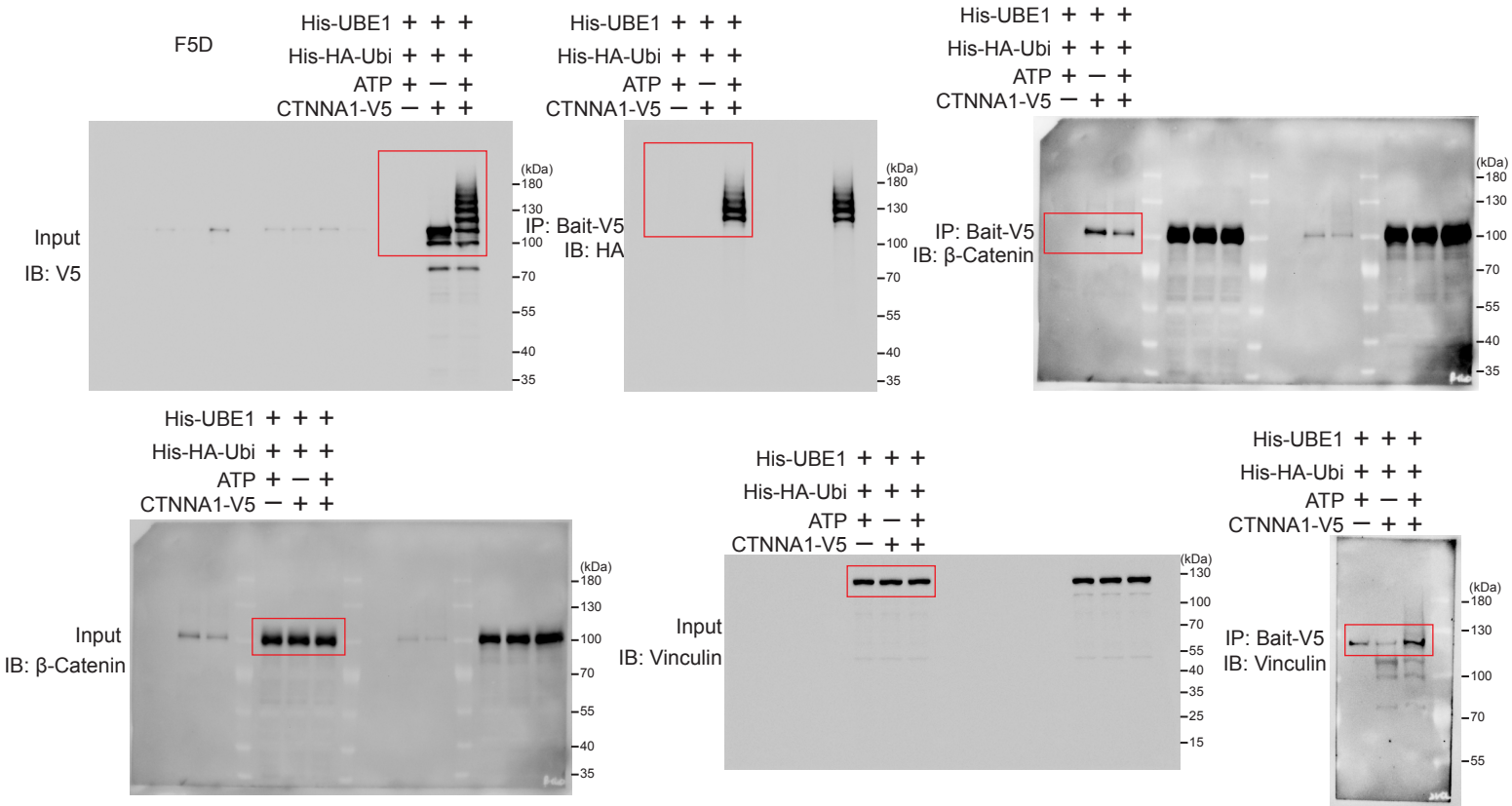

Supplement: Supplementary file 9 — Source data Fig. 5 [file 44319_2025_585_MOESM9_ESM.zip › EMBOR202561827V2_SourceDataForFigure5/5D/Figure5D_Blots.pdf]

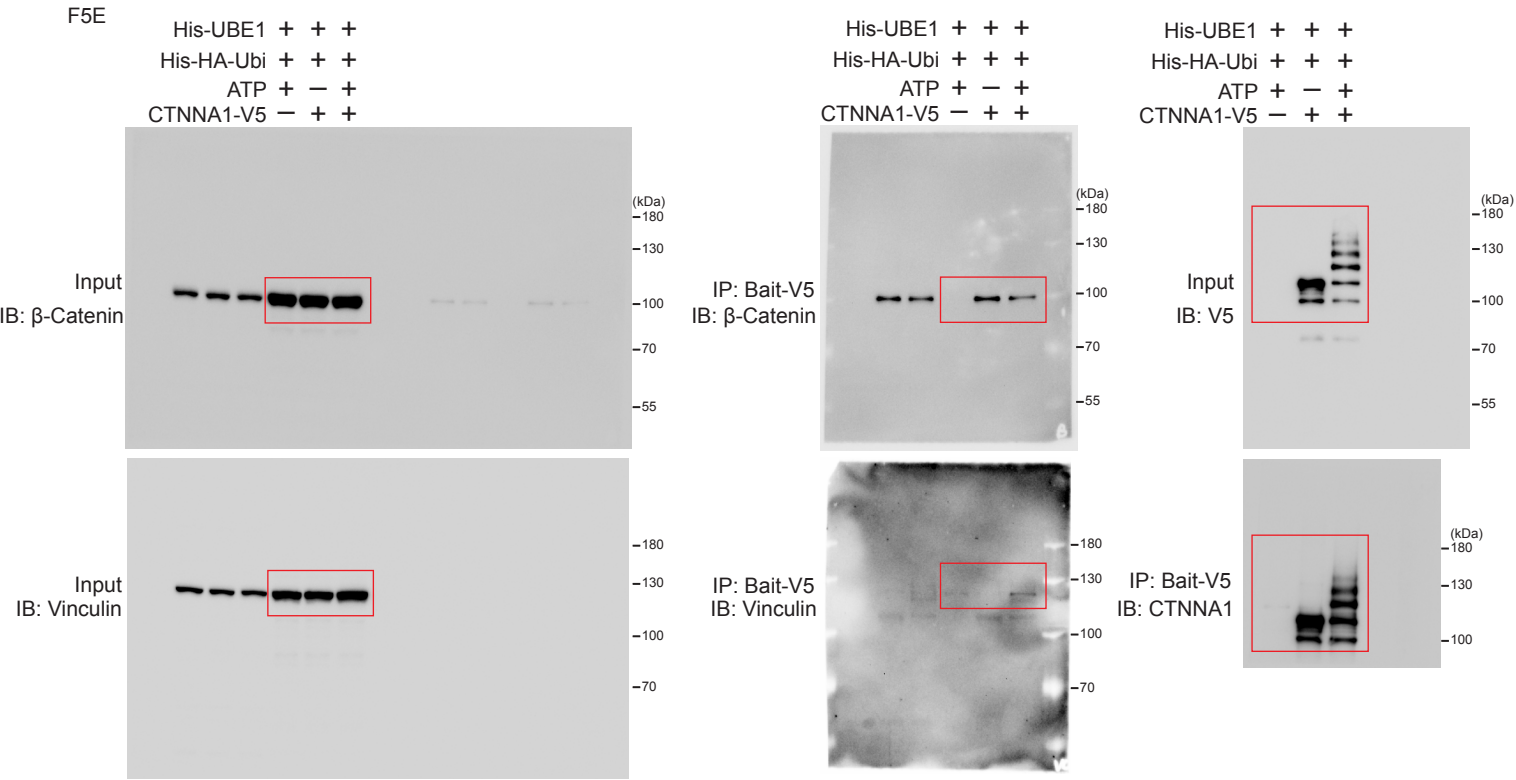

Supplement: Supplementary file 9 — Source data Fig. 5 [file 44319_2025_585_MOESM9_ESM.zip › EMBOR202561827V2_SourceDataForFigure5/5E/Figure5E_Blots.pdf]

log2 Centered intensity

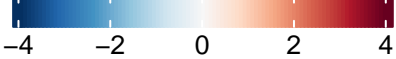

1

2

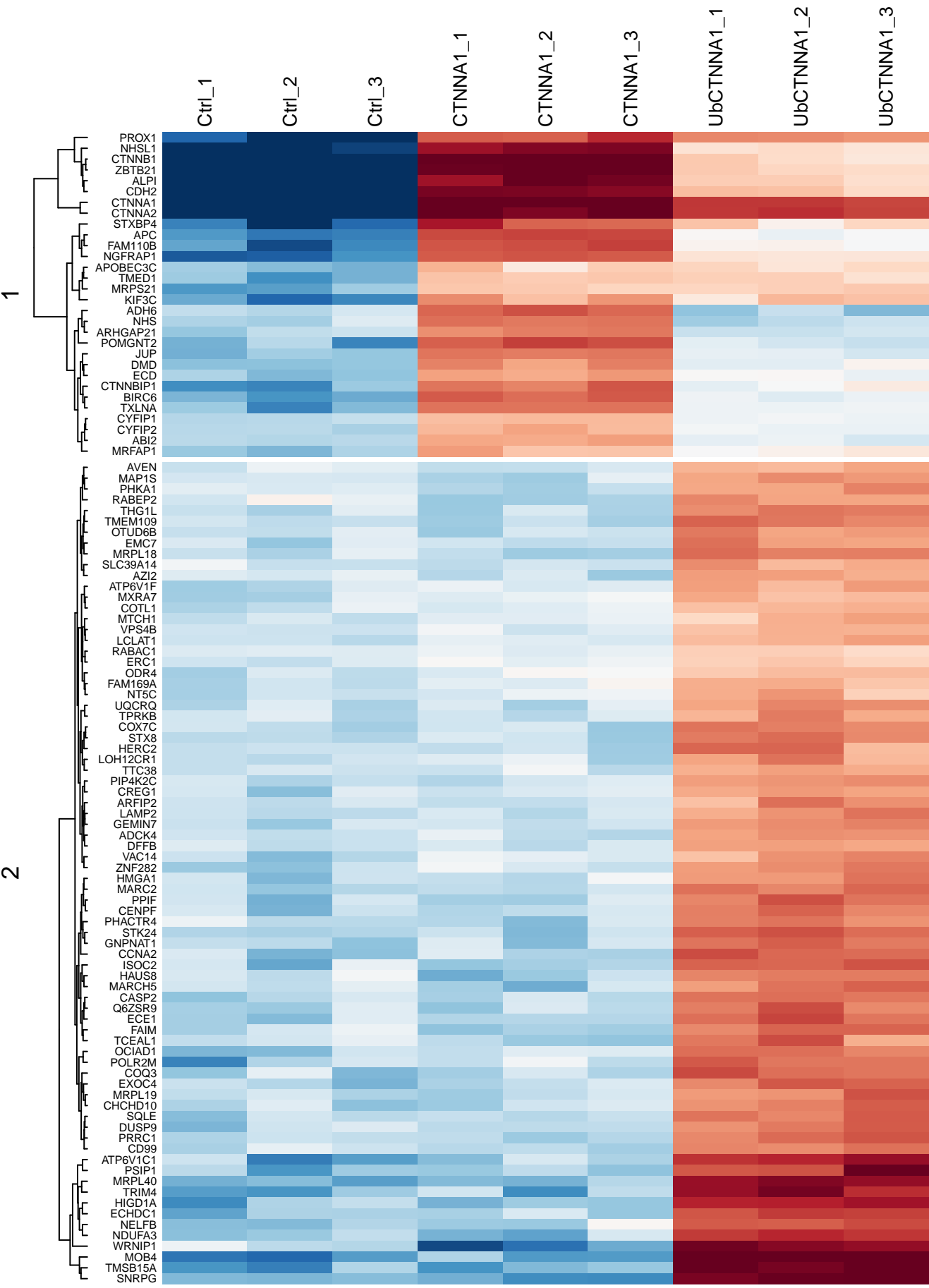

Supplement: Supplementary file 9 — Source data Fig. 5 [file 44319_2025_585_MOESM9_ESM.zip › EMBOR202561827V2_SourceDataForFigure5/5B/Figure5B_DEP2_Heatmap.pdf]

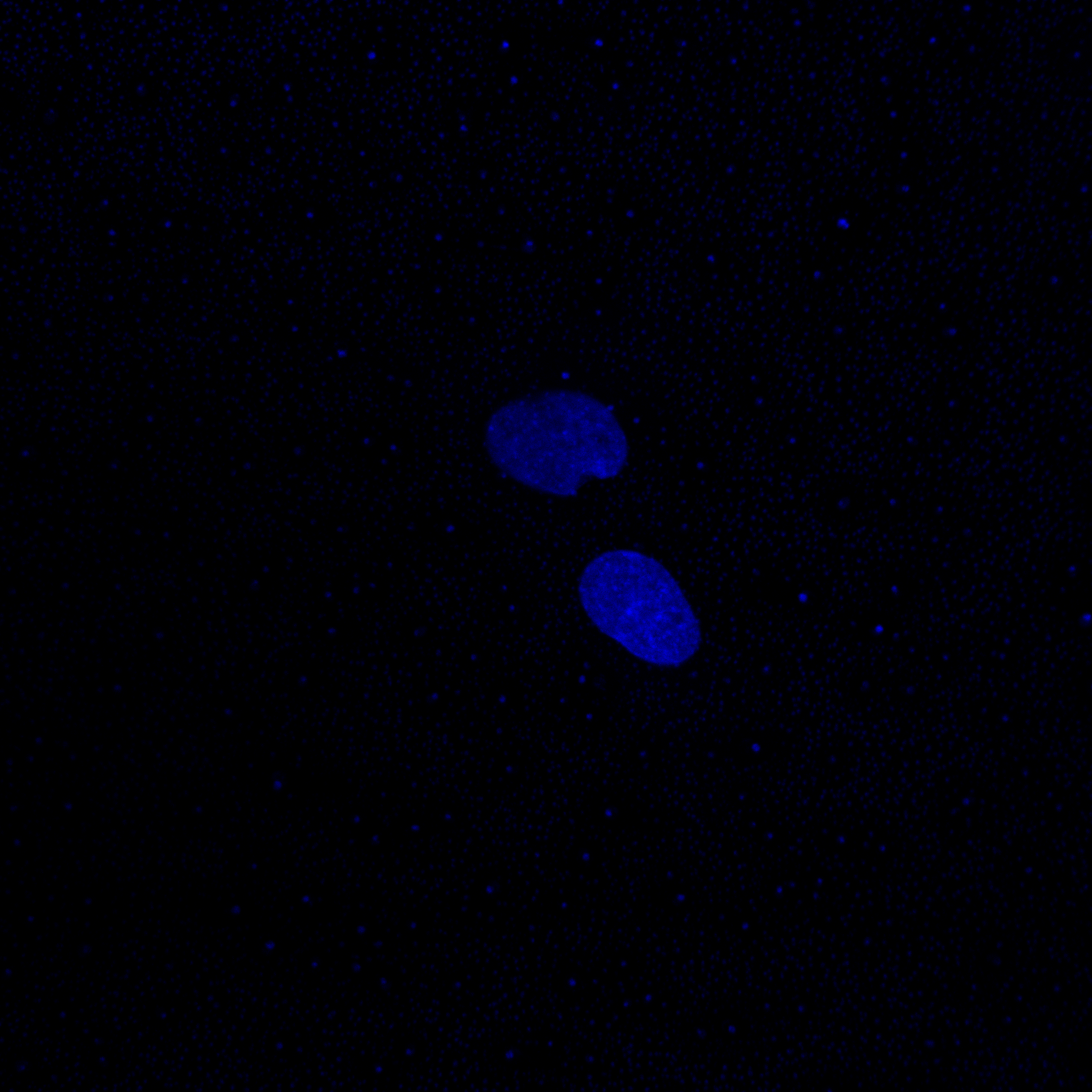

Supplement: Supplementary file 11 — Source data Fig. 7 [file 44319_2025_585_MOESM11_ESM.zip › EMBOR202561827V2_SourceDataForFigure7/7D/Figure7D_ConfocalImage_Res_Vector_DAPI.tif]

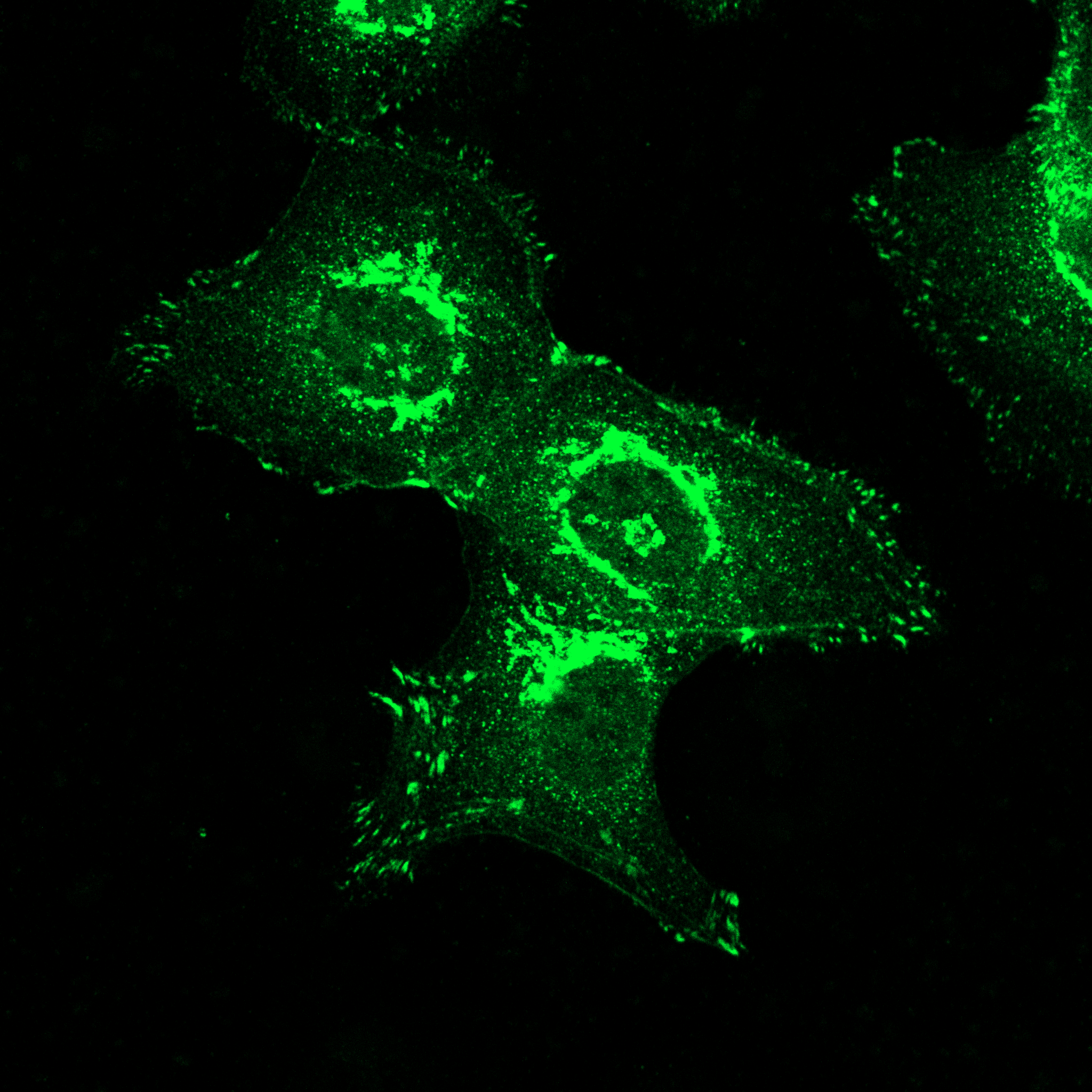

Supplement: Supplementary file 11 — Source data Fig. 7 [file 44319_2025_585_MOESM11_ESM.zip › EMBOR202561827V2_SourceDataForFigure7/7D/Figure7D_ConfocalImage_siNC_Zyxin_AlexaFluor488.tif]

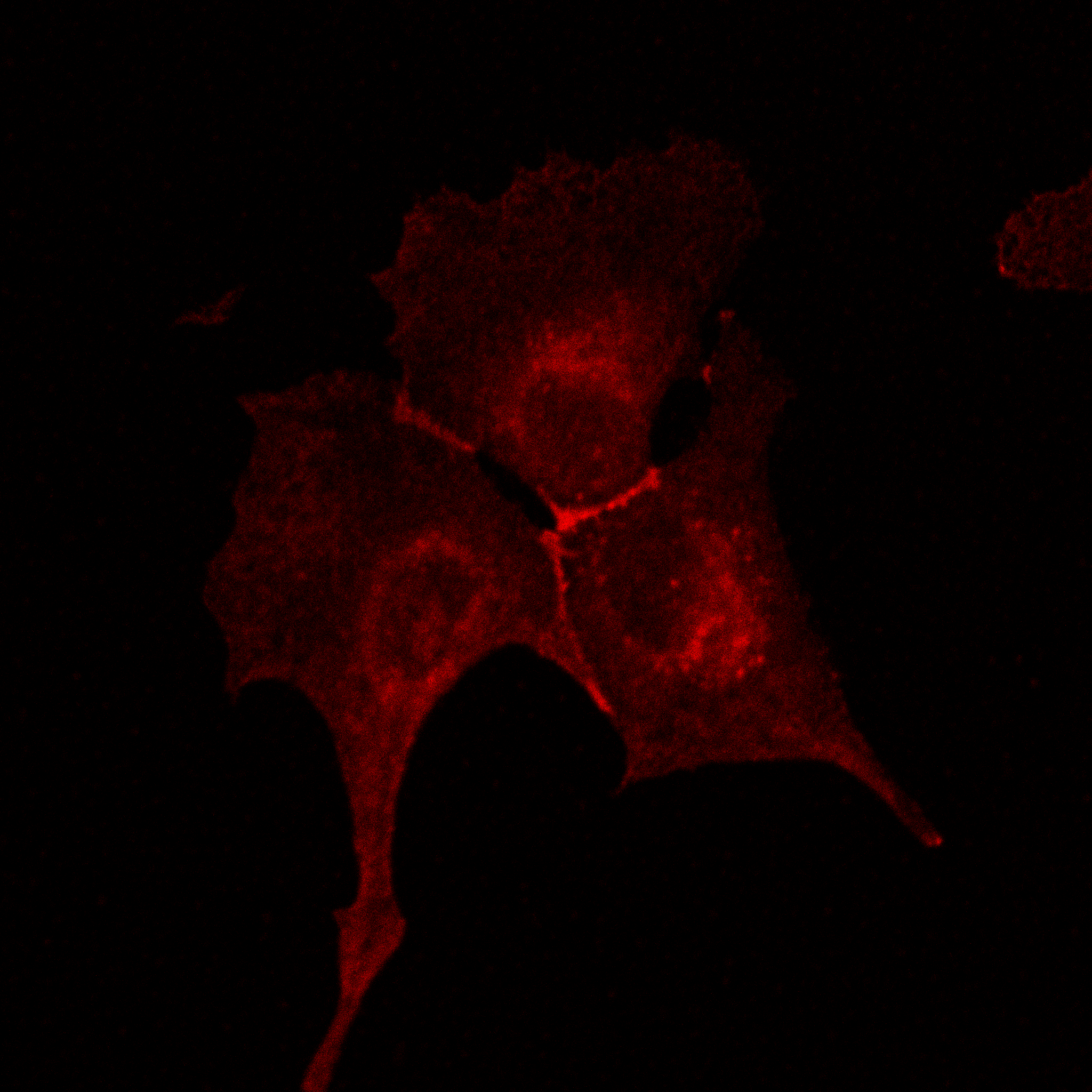

Supplement: Supplementary file 11 — Source data Fig. 7 [file 44319_2025_585_MOESM11_ESM.zip › EMBOR202561827V2_SourceDataForFigure7/7D/Figure7D_ConfocalImage_Res_UBE2O-CS_CTNNB1_AlexaFluor568.tif]

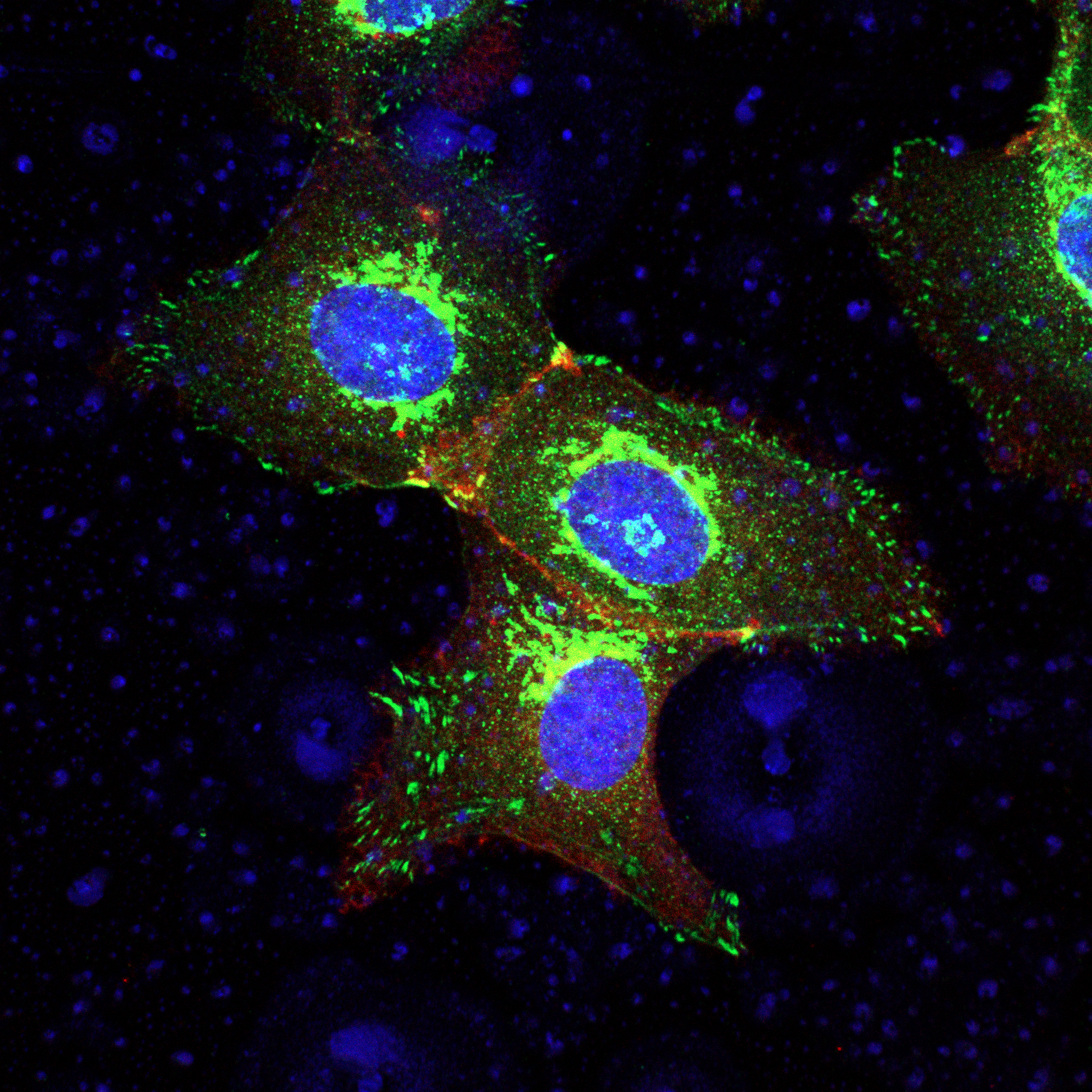

Supplement: Supplementary file 11 — Source data Fig. 7 [file 44319_2025_585_MOESM11_ESM.zip › EMBOR202561827V2_SourceDataForFigure7/7D/Figure7D_ConfocalImage_siNC_Merge.tif]

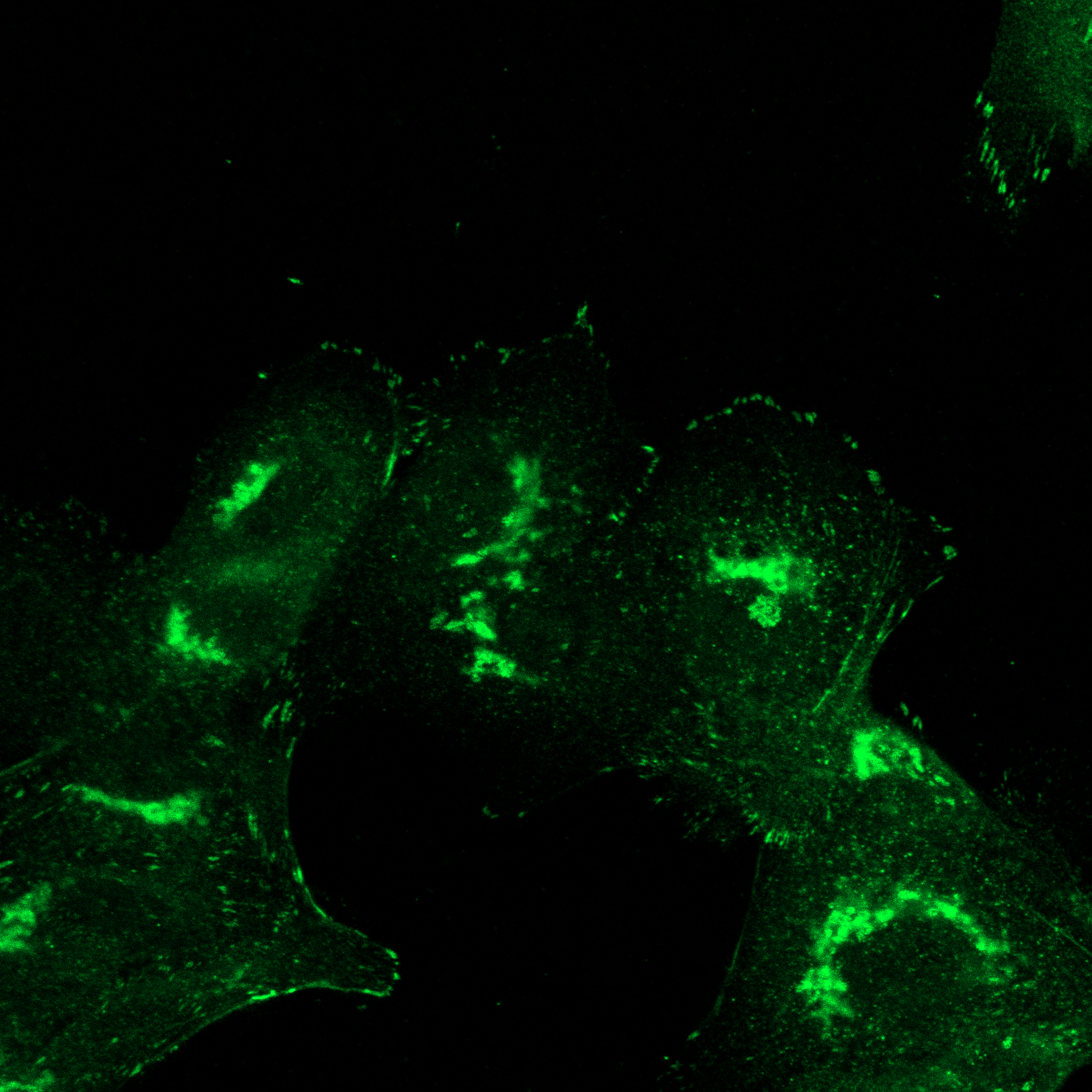

Supplement: Supplementary file 11 — Source data Fig. 7 [file 44319_2025_585_MOESM11_ESM.zip › EMBOR202561827V2_SourceDataForFigure7/7D/Figure7D_ConfocalImage_siUBE2O_Zyxin_AlexaFluor488.tif]

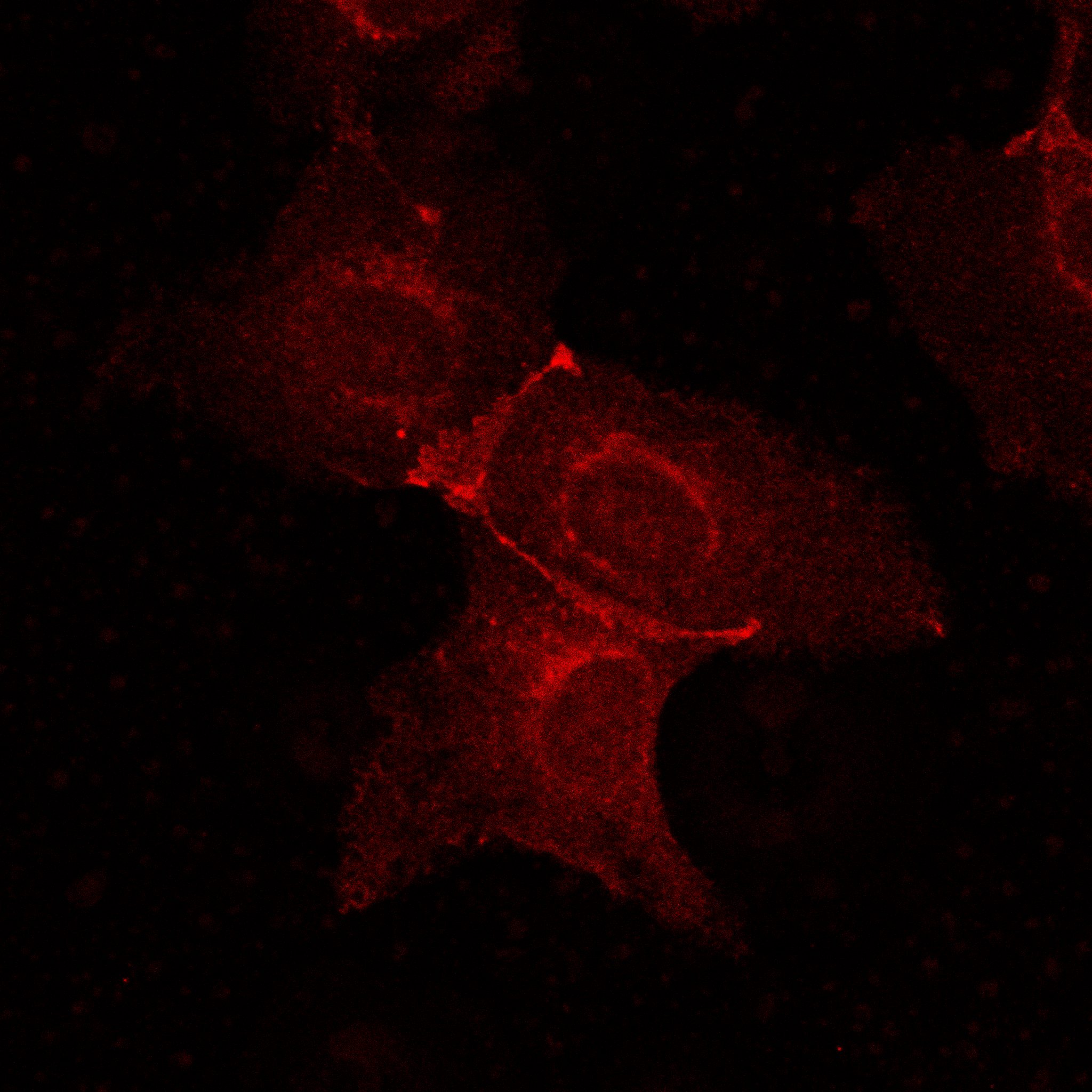

Supplement: Supplementary file 11 — Source data Fig. 7 [file 44319_2025_585_MOESM11_ESM.zip › EMBOR202561827V2_SourceDataForFigure7/7D/Figure7D_ConfocalImage_siNC_CTNNB1_AlexaFluor568.tif]

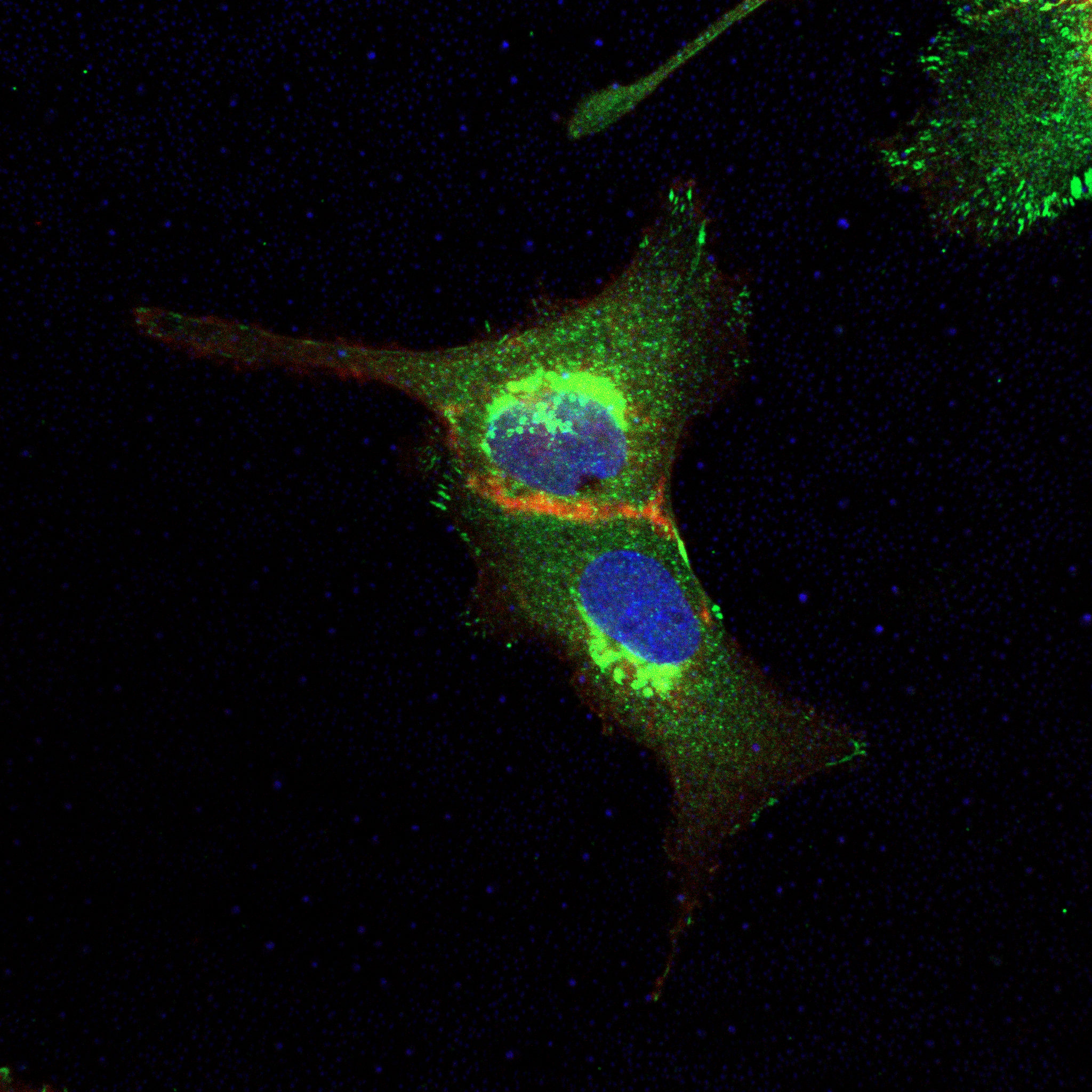

Supplement: Supplementary file 11 — Source data Fig. 7 [file 44319_2025_585_MOESM11_ESM.zip › EMBOR202561827V2_SourceDataForFigure7/7D/Figure7D_ConfocalImage_Res_Vector_Merge.tif]

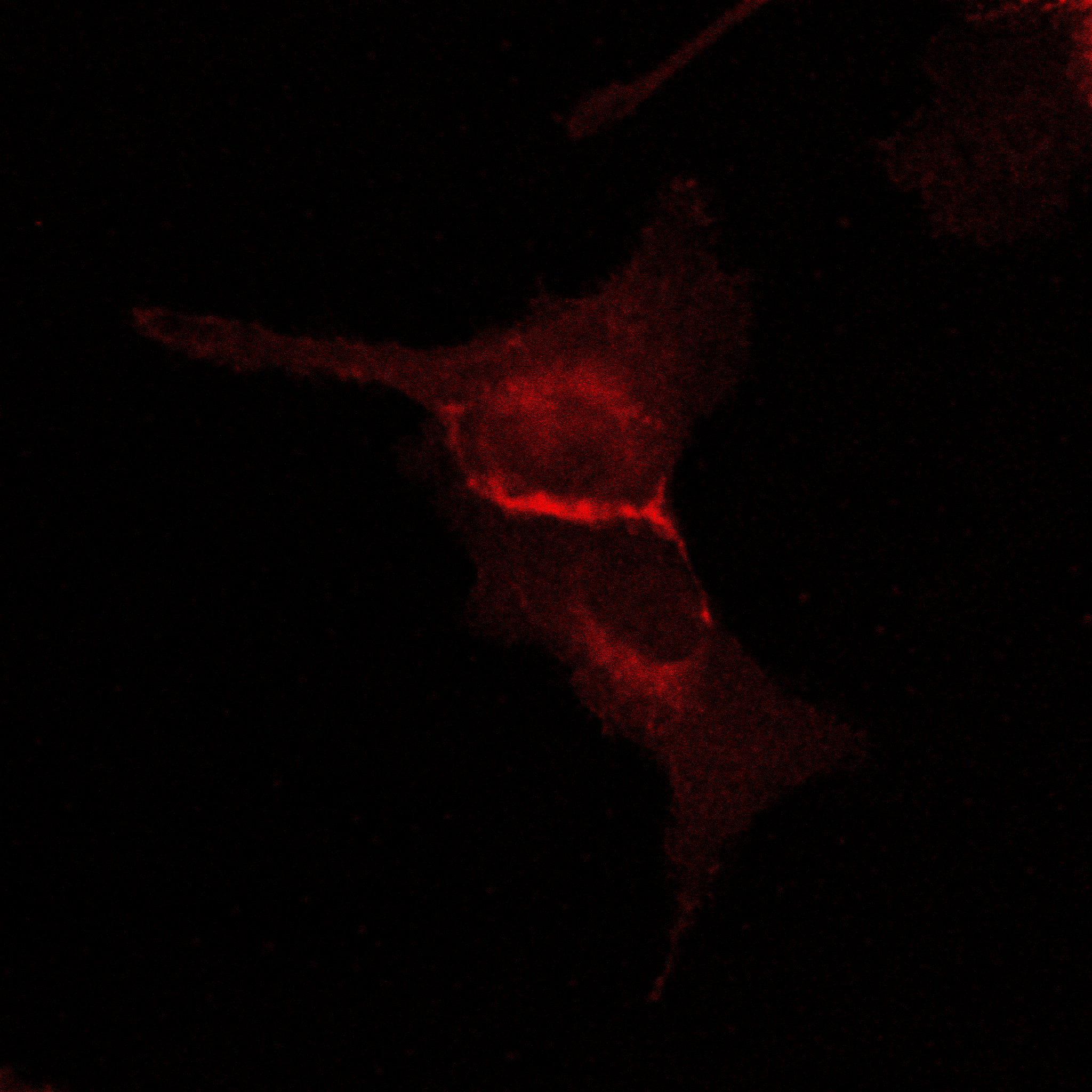

Supplement: Supplementary file 11 — Source data Fig. 7 [file 44319_2025_585_MOESM11_ESM.zip › EMBOR202561827V2_SourceDataForFigure7/7D/Figure7D_ConfocalImage_Res_Vector_CTNNB1_AlexaFluor568.tif]

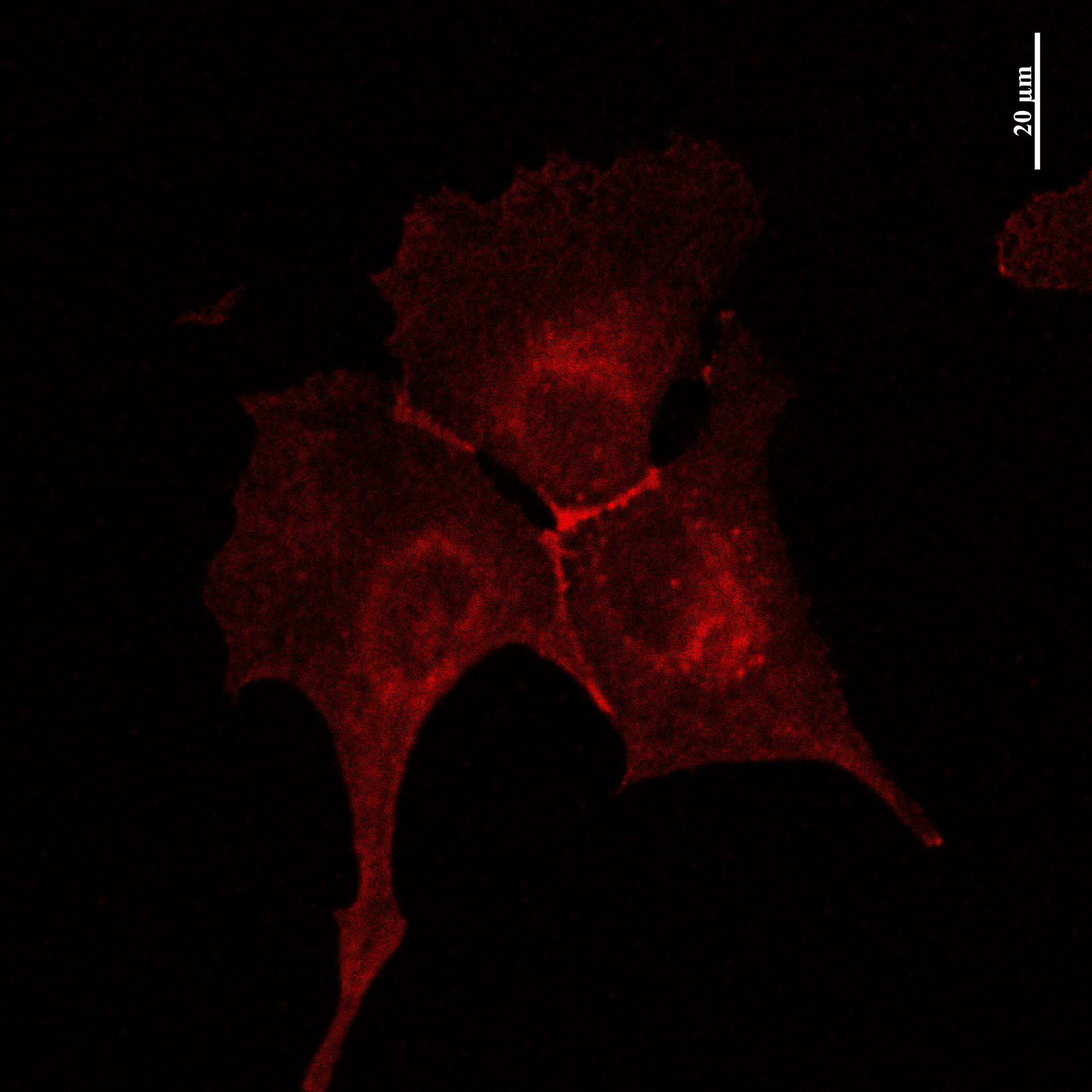

Supplement: Supplementary file 11 — Source data Fig. 7 [file 44319_2025_585_MOESM11_ESM.zip › EMBOR202561827V2_SourceDataForFigure7/7D/ScaleBar_Figure7D_ConfocalImage.tif]

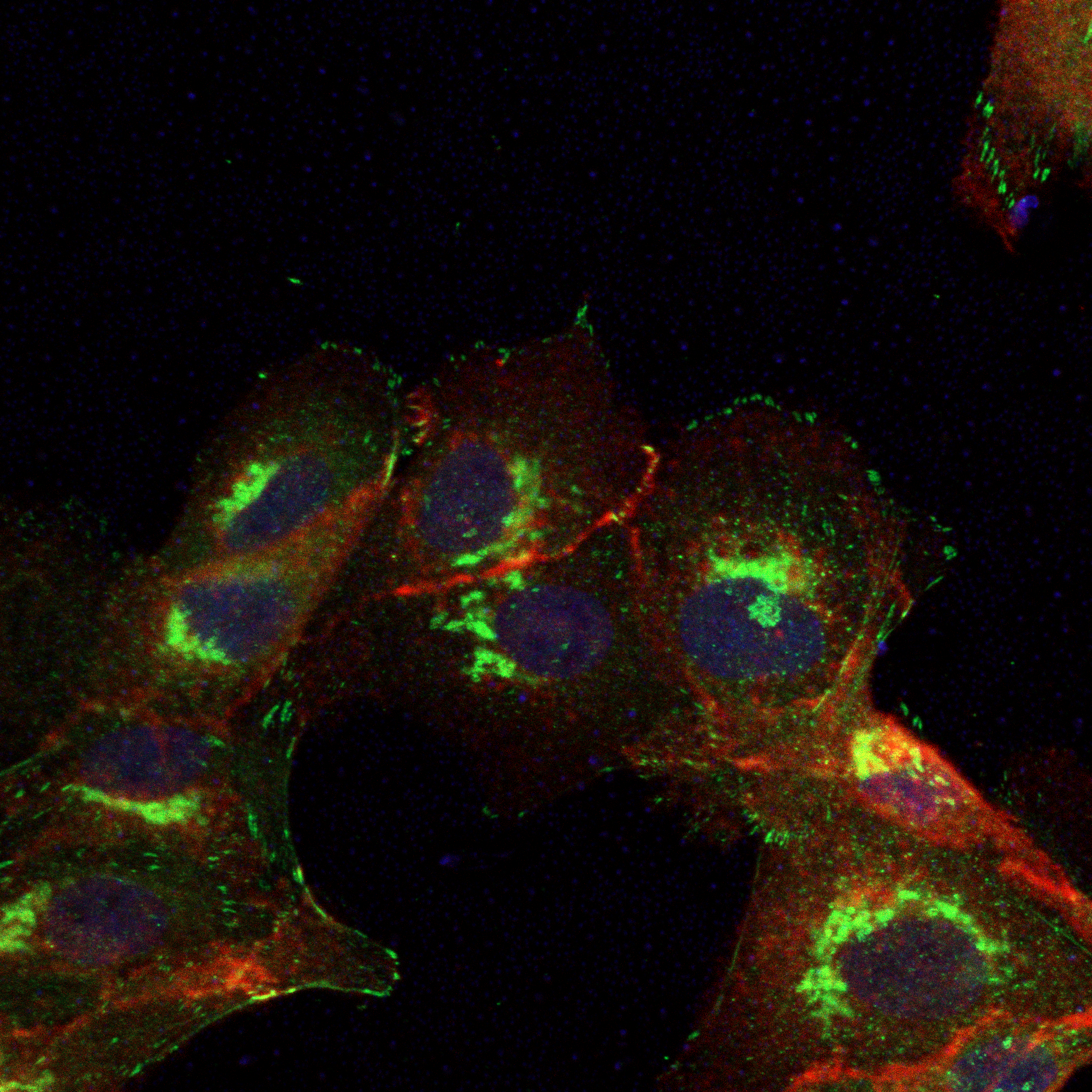

Supplement: Supplementary file 11 — Source data Fig. 7 [file 44319_2025_585_MOESM11_ESM.zip › EMBOR202561827V2_SourceDataForFigure7/7D/Figure7D_ConfocalImage_siUBE2O_Merge.tif]

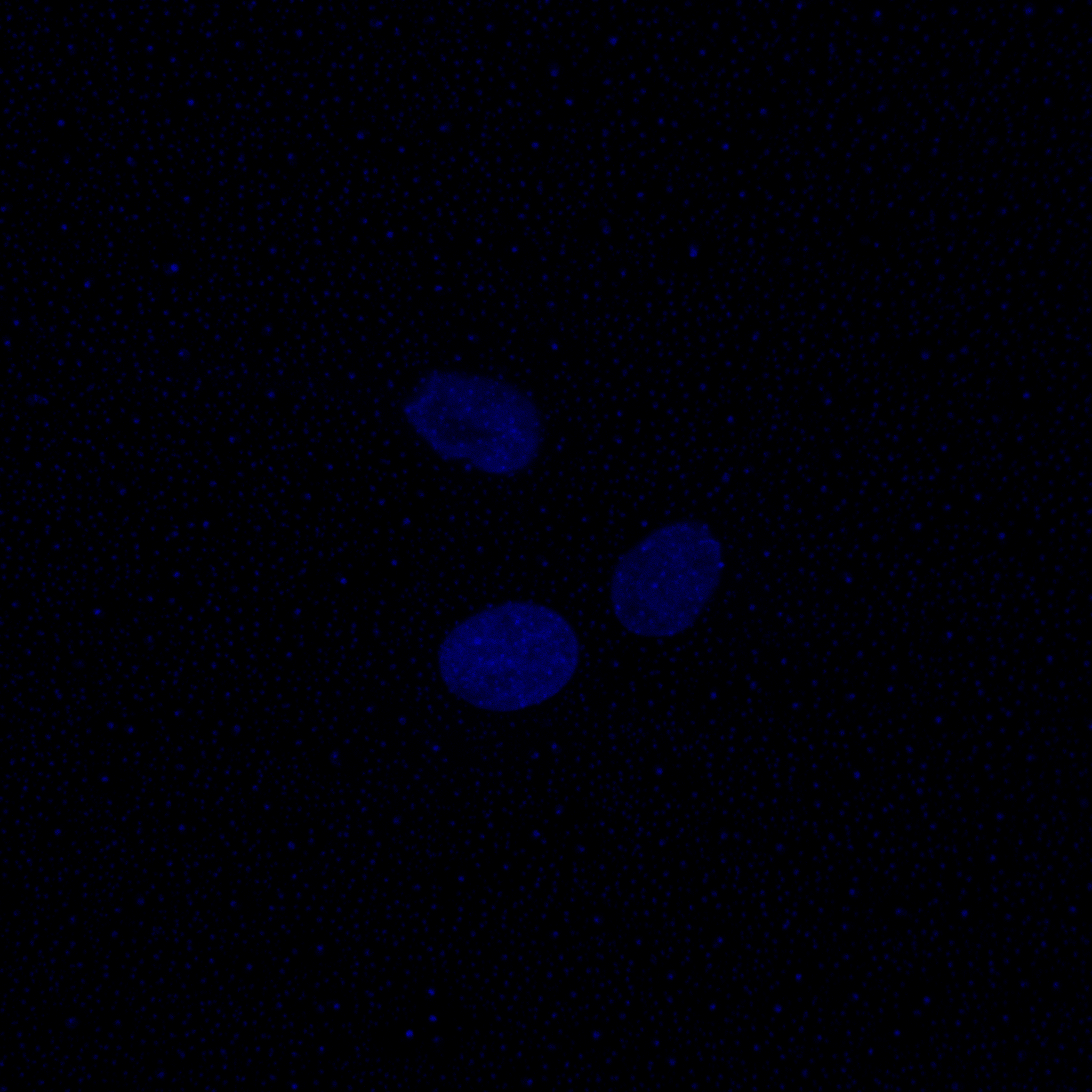

Supplement: Supplementary file 11 — Source data Fig. 7 [file 44319_2025_585_MOESM11_ESM.zip › EMBOR202561827V2_SourceDataForFigure7/7D/Figure7D_ConfocalImage_Res_UBE2O-CS_DAPI.tif]

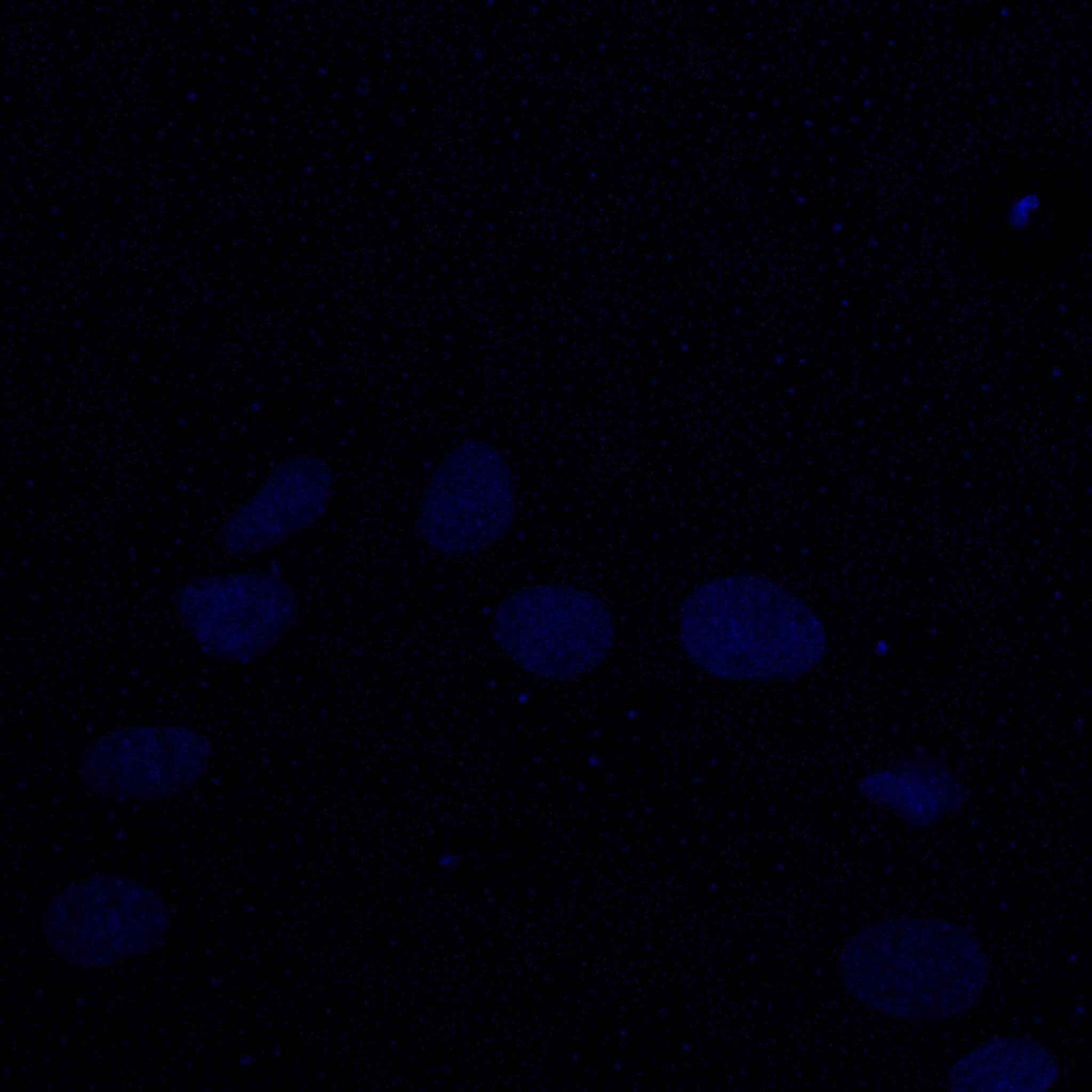

Supplement: Supplementary file 11 — Source data Fig. 7 [file 44319_2025_585_MOESM11_ESM.zip › EMBOR202561827V2_SourceDataForFigure7/7D/Figure7D_ConfocalImage_siUBE2O_DAPI.tif]

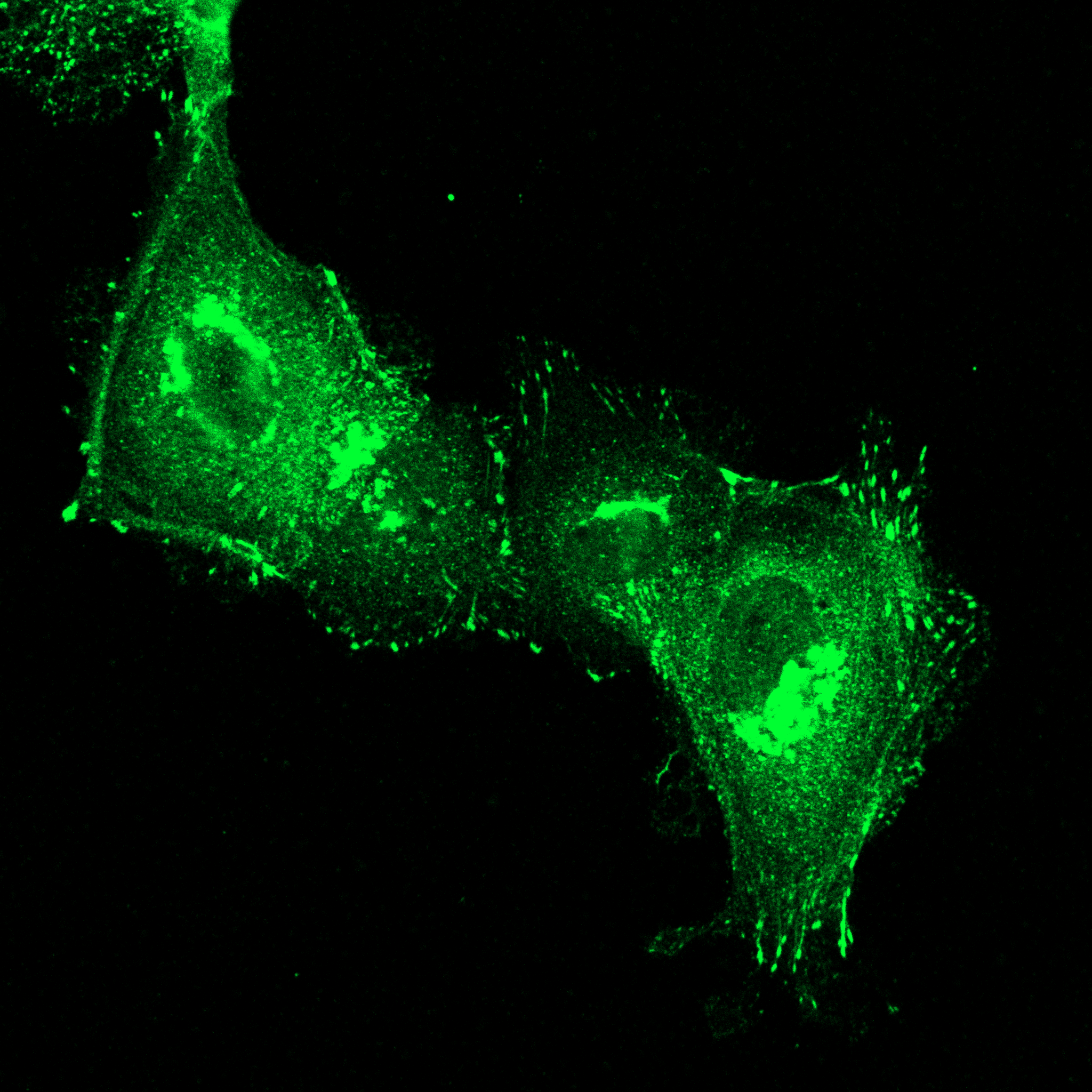

Supplement: Supplementary file 11 — Source data Fig. 7 [file 44319_2025_585_MOESM11_ESM.zip › EMBOR202561827V2_SourceDataForFigure7/7D/Figure7D_ConfocalImage_Res_UBE2O-WT_Zyxin_AlexaFluor488.tif]

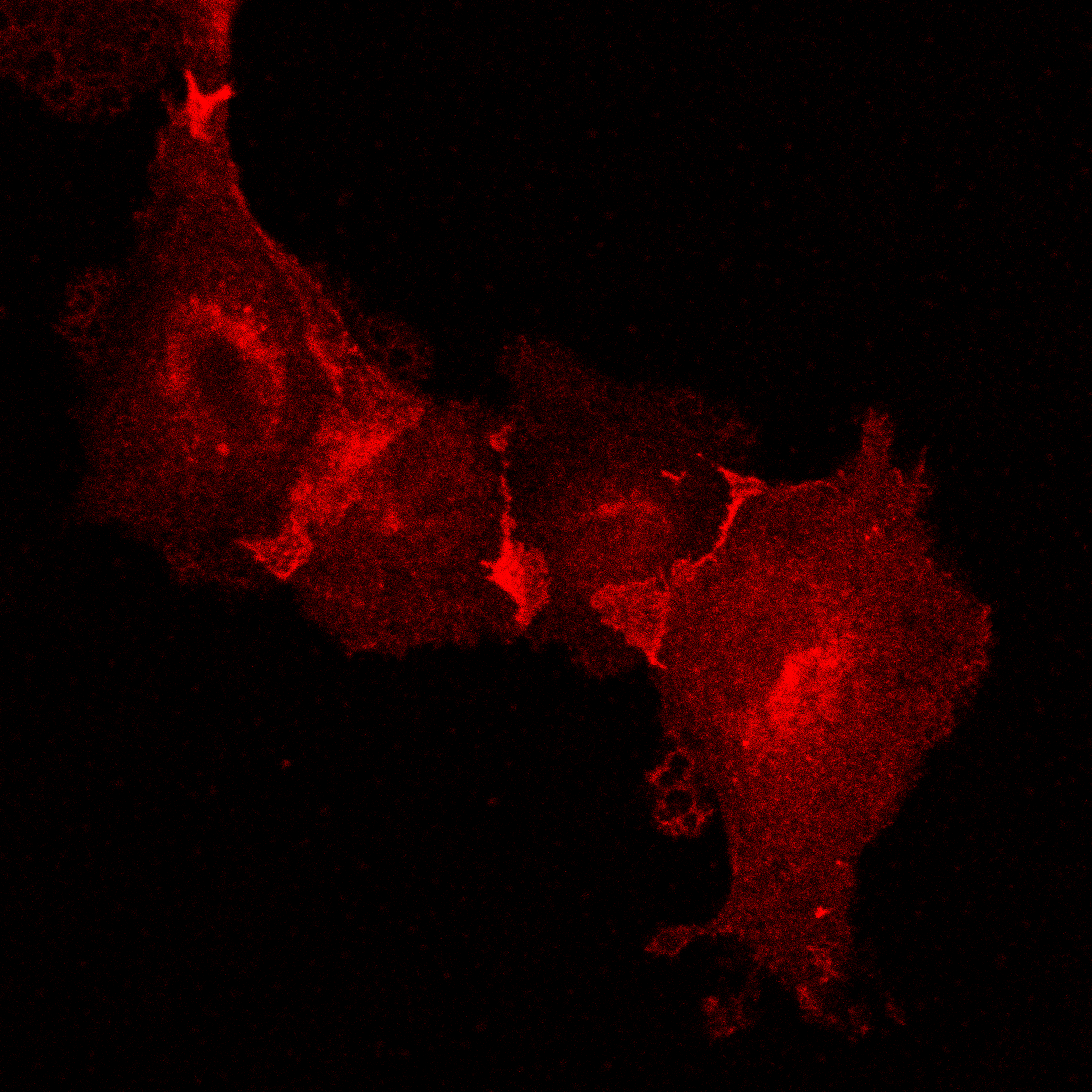

Supplement: Supplementary file 11 — Source data Fig. 7 [file 44319_2025_585_MOESM11_ESM.zip › EMBOR202561827V2_SourceDataForFigure7/7D/Figure7D_ConfocalImage_Res_UBE2O-WT_CTNNB1_AlexaFluor568.tif]

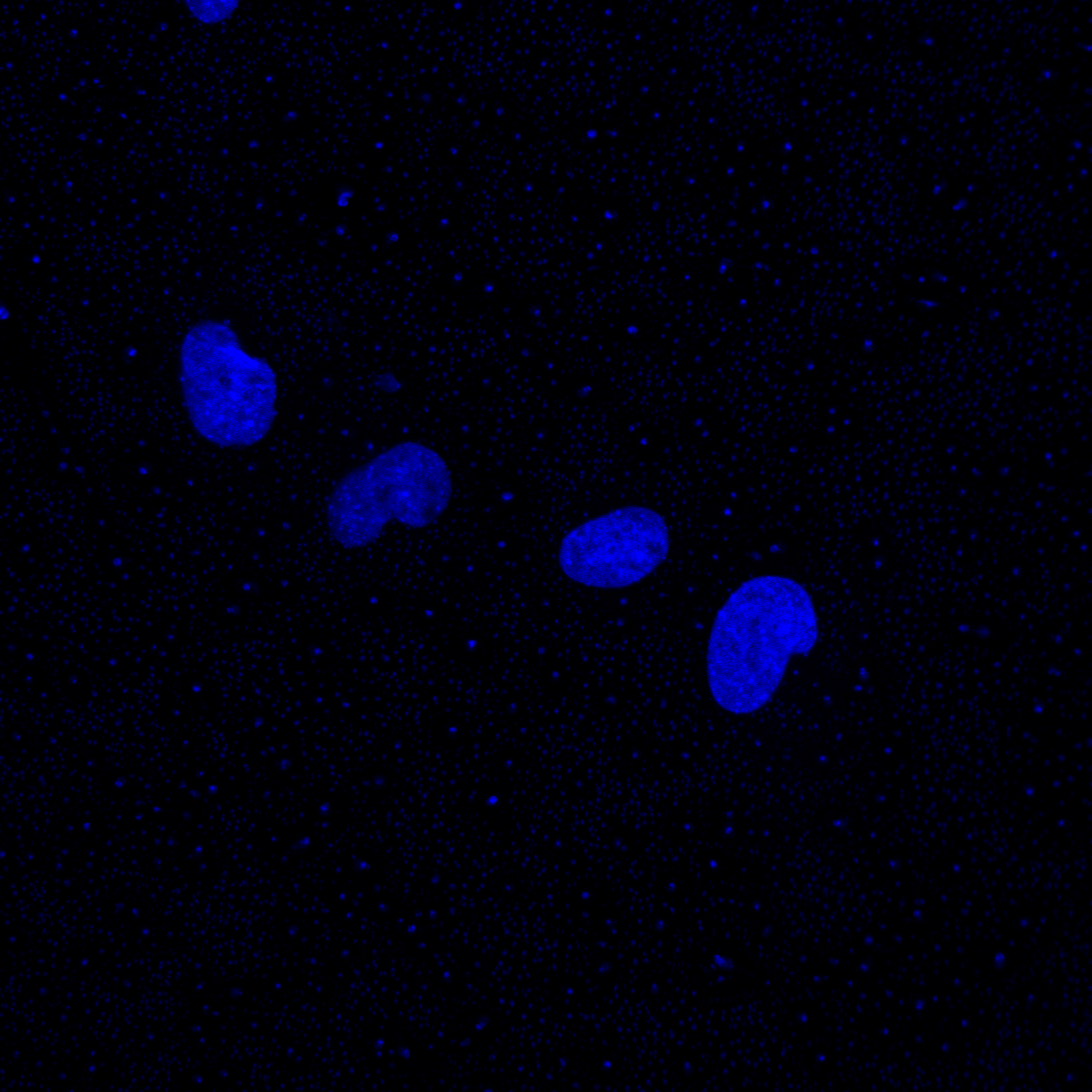

Supplement: Supplementary file 11 — Source data Fig. 7 [file 44319_2025_585_MOESM11_ESM.zip › EMBOR202561827V2_SourceDataForFigure7/7D/Figure7D_ConfocalImage_Res_UBE2O-WT_DAPI.tif]

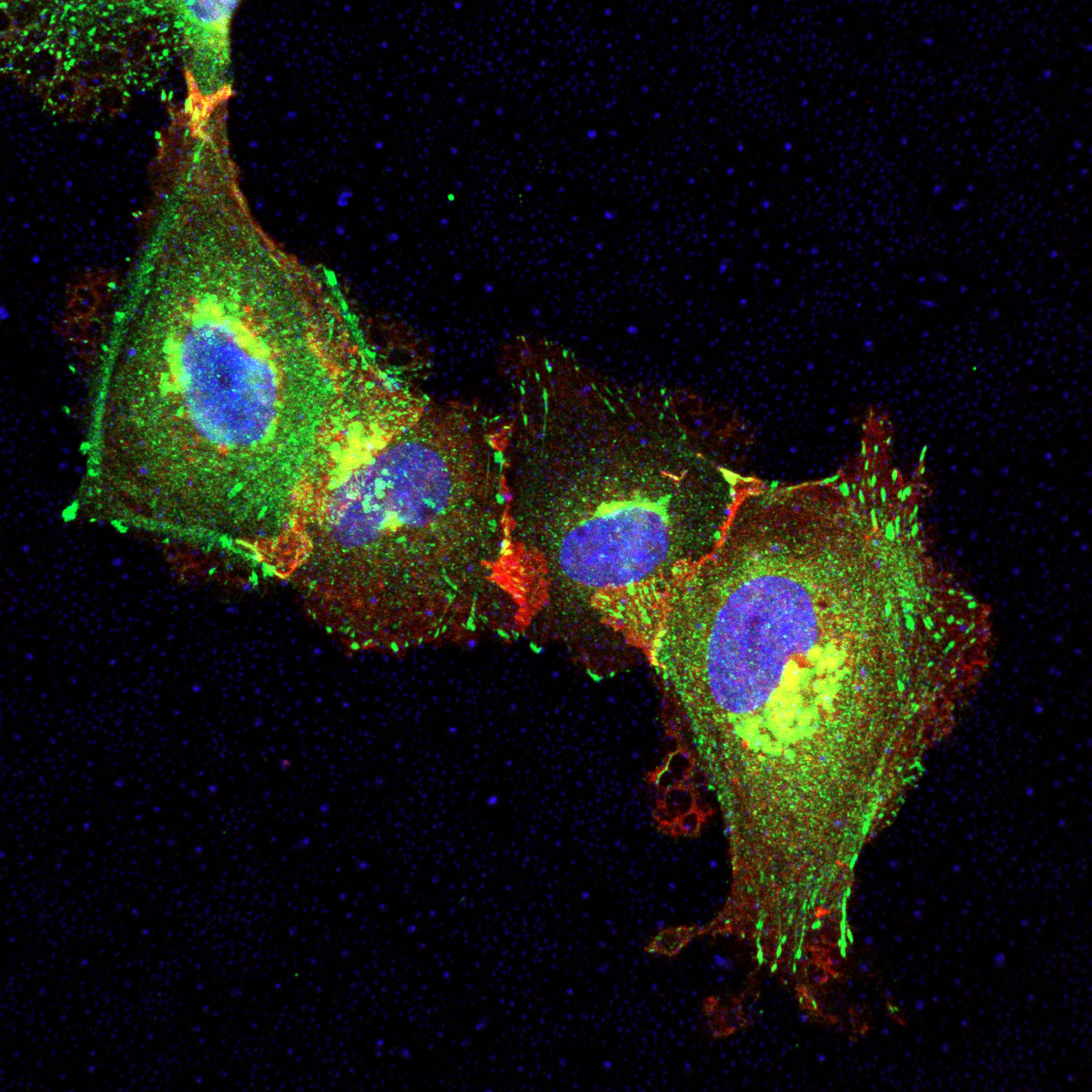

Supplement: Supplementary file 11 — Source data Fig. 7 [file 44319_2025_585_MOESM11_ESM.zip › EMBOR202561827V2_SourceDataForFigure7/7D/Figure7D_ConfocalImage_Res_UBE2O-WT_Merge.tif]

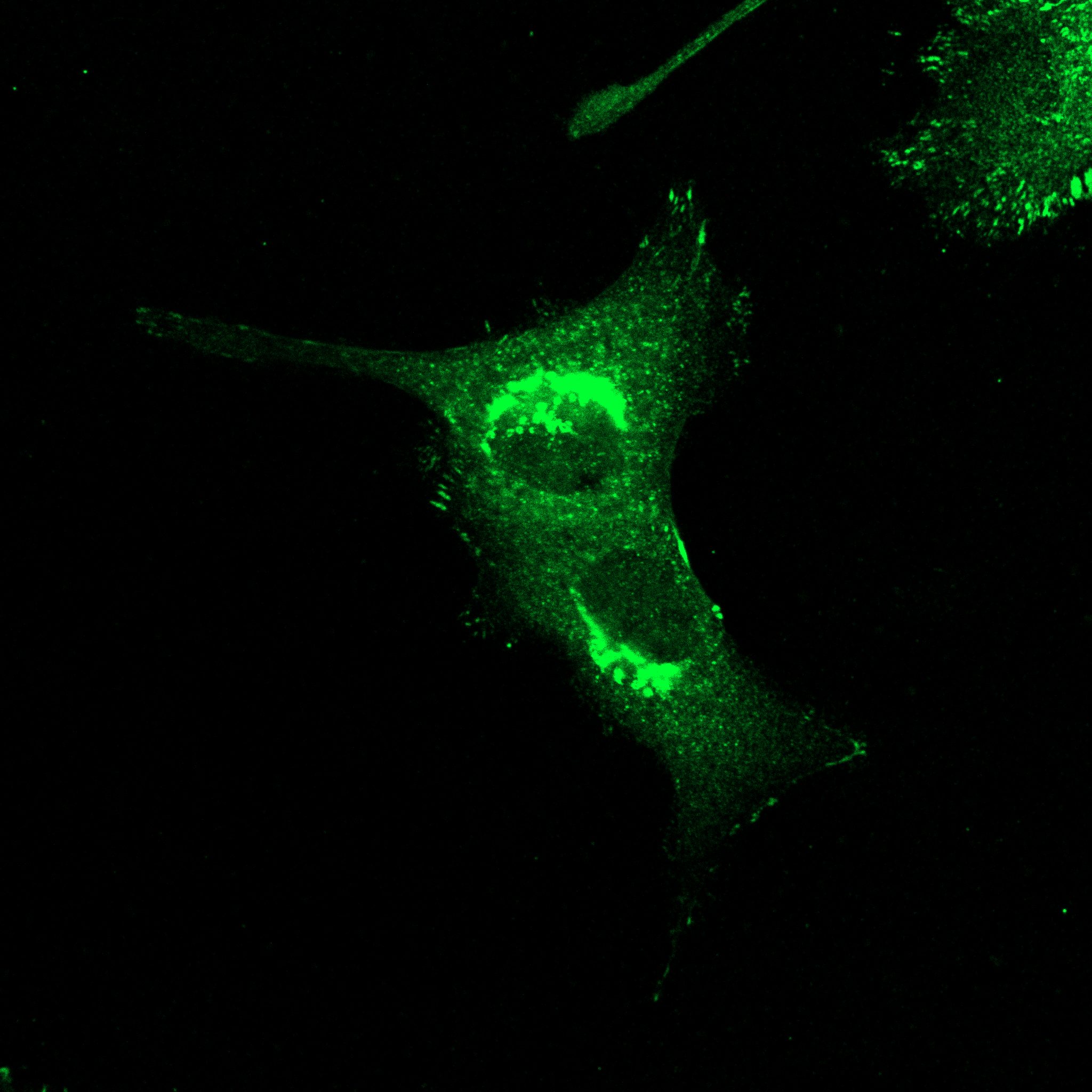

Supplement: Supplementary file 11 — Source data Fig. 7 [file 44319_2025_585_MOESM11_ESM.zip › EMBOR202561827V2_SourceDataForFigure7/7D/Figure7D_ConfocalImage_Res_Vector_Zyxin_AlexaFluor488.tif]

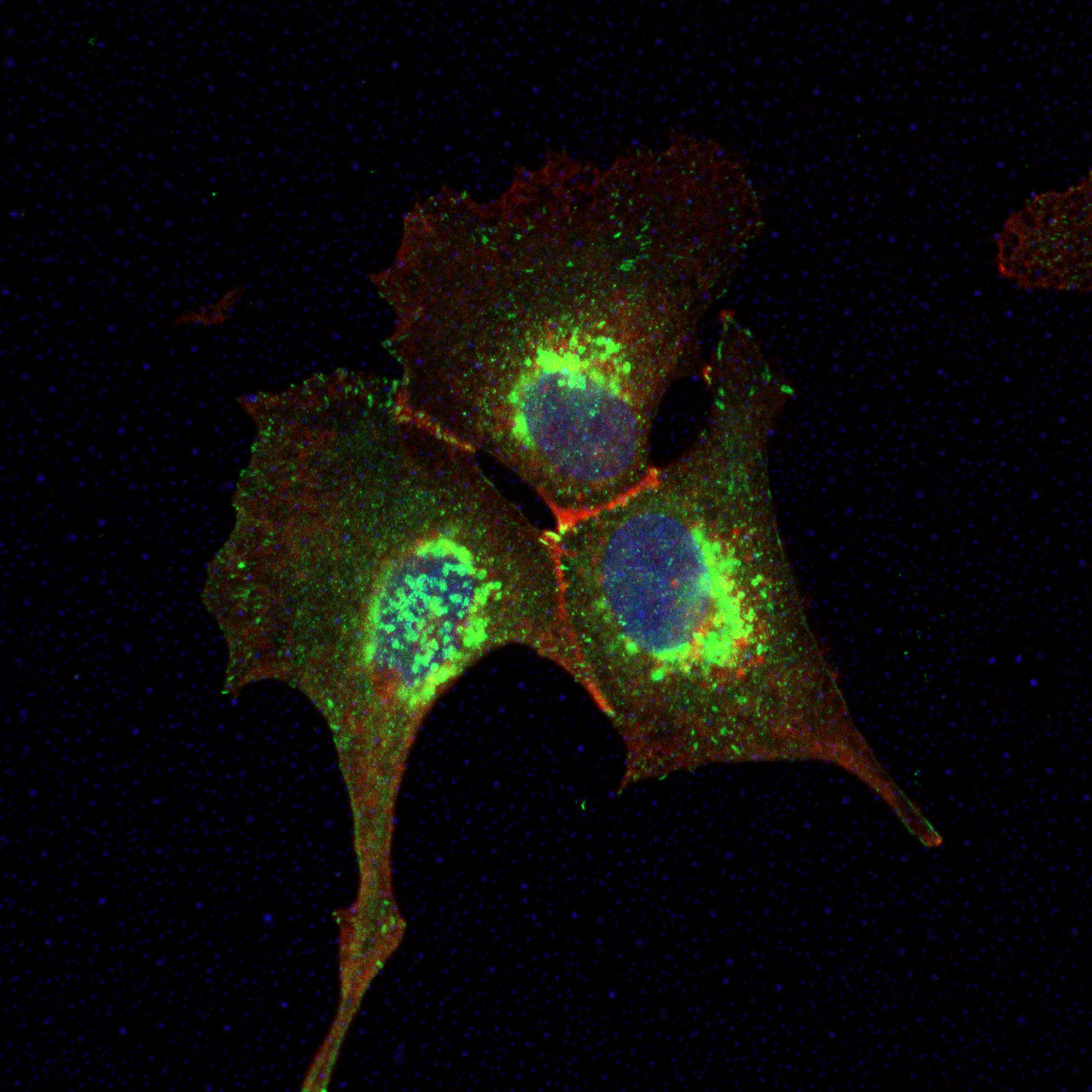

Supplement: Supplementary file 11 — Source data Fig. 7 [file 44319_2025_585_MOESM11_ESM.zip › EMBOR202561827V2_SourceDataForFigure7/7D/Figure7D_ConfocalImage_Res_UBE2O-CS_Merge.tif]

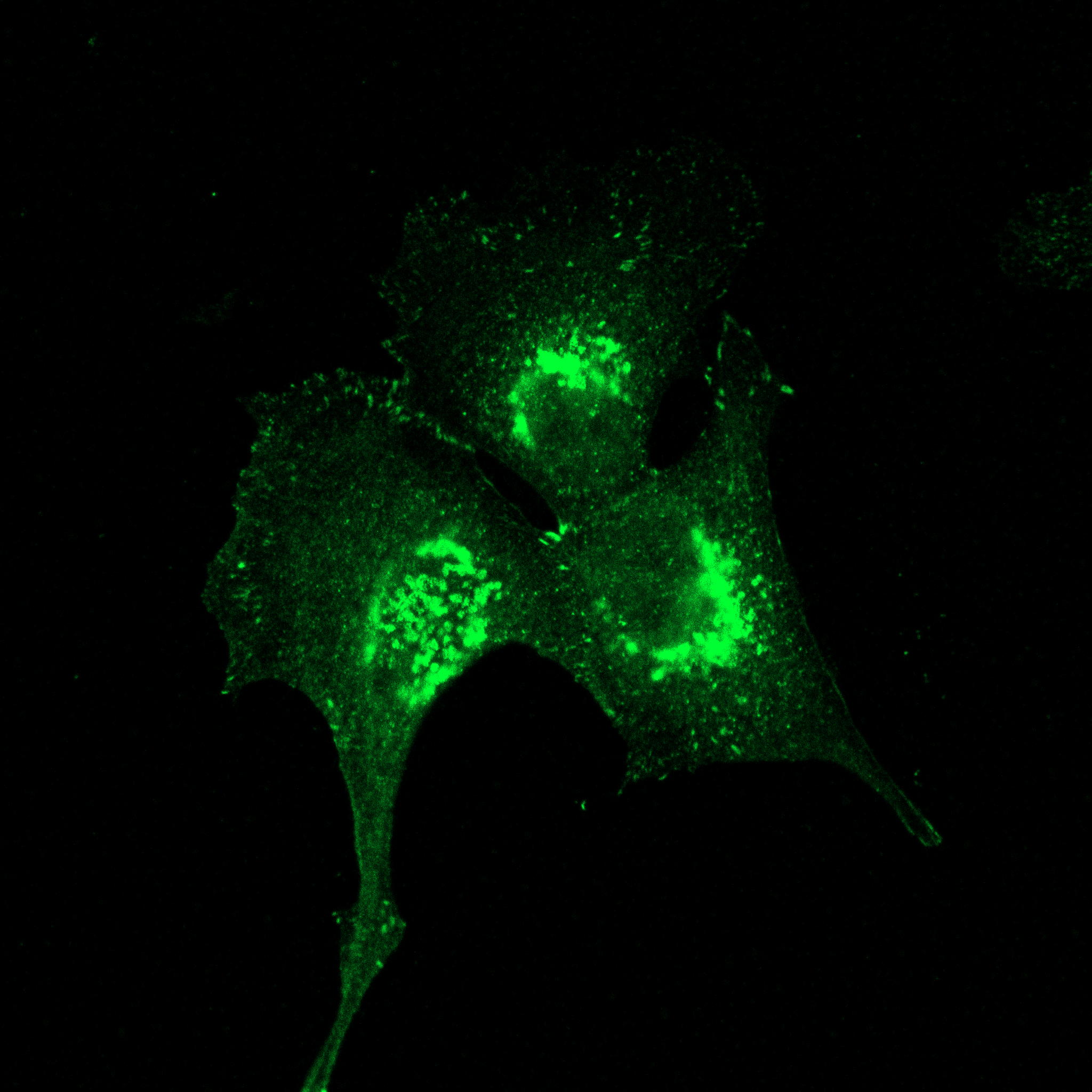

Supplement: Supplementary file 11 — Source data Fig. 7 [file 44319_2025_585_MOESM11_ESM.zip › EMBOR202561827V2_SourceDataForFigure7/7D/Figure7D_ConfocalImage_Res_UBE2O-CS_Zyxin_AlexaFluor488.tif]

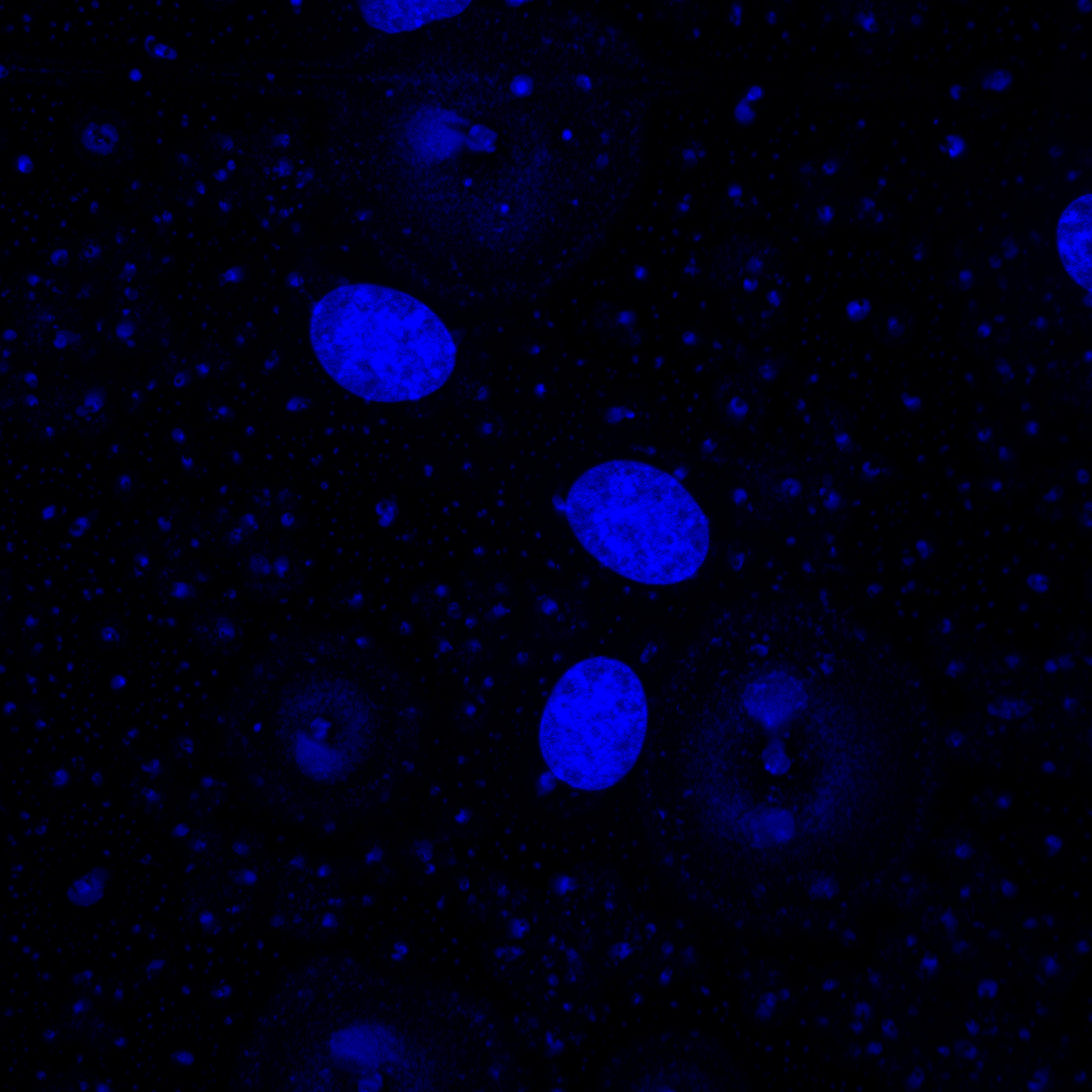

Supplement: Supplementary file 11 — Source data Fig. 7 [file 44319_2025_585_MOESM11_ESM.zip › EMBOR202561827V2_SourceDataForFigure7/7D/Figure7D_ConfocalImage_siNC_DAPI.tif]

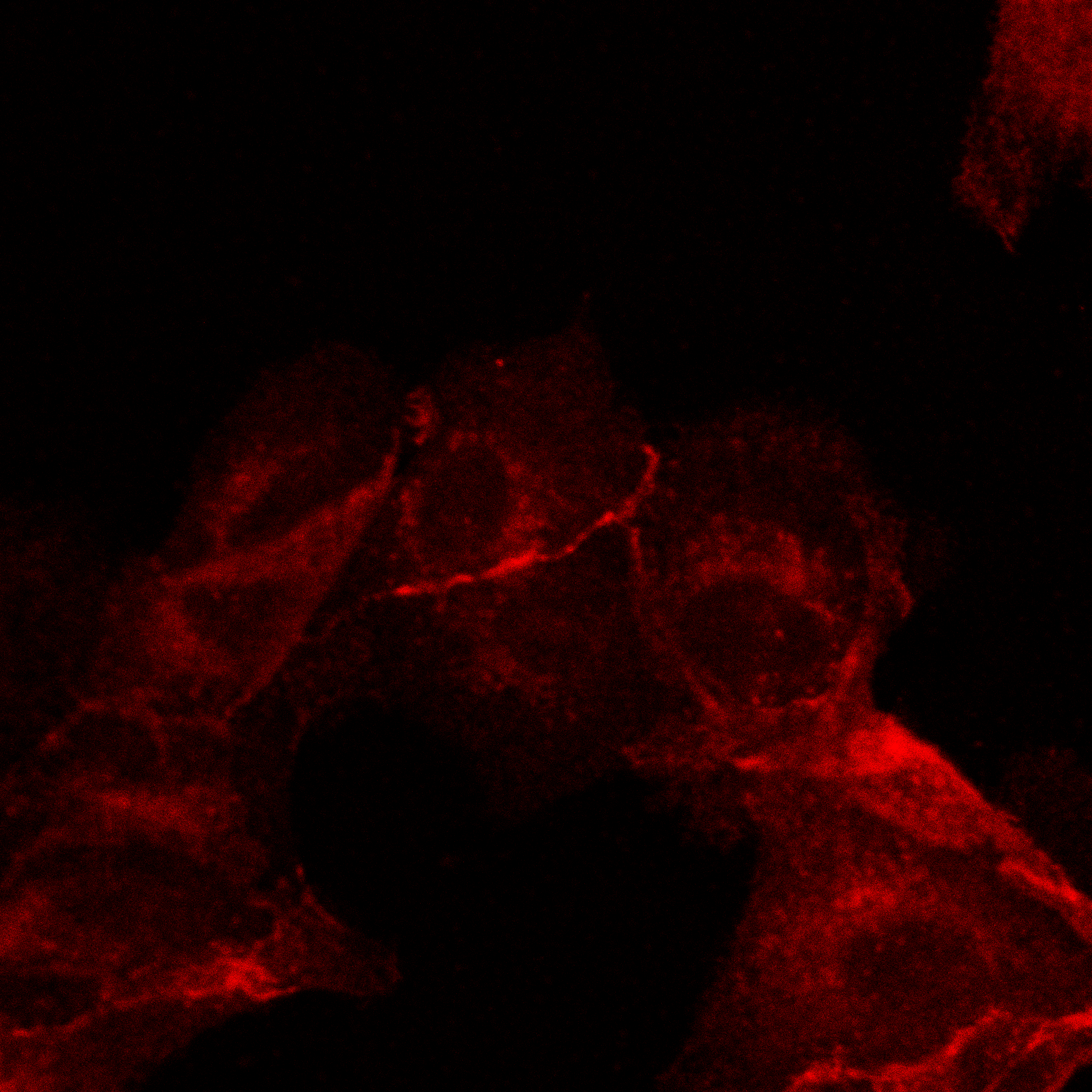

Supplement: Supplementary file 11 — Source data Fig. 7 [file 44319_2025_585_MOESM11_ESM.zip › EMBOR202561827V2_SourceDataForFigure7/7D/Figure7D_ConfocalImage_siUBE2O_CTNNB1_AlexaFluor568.tif]

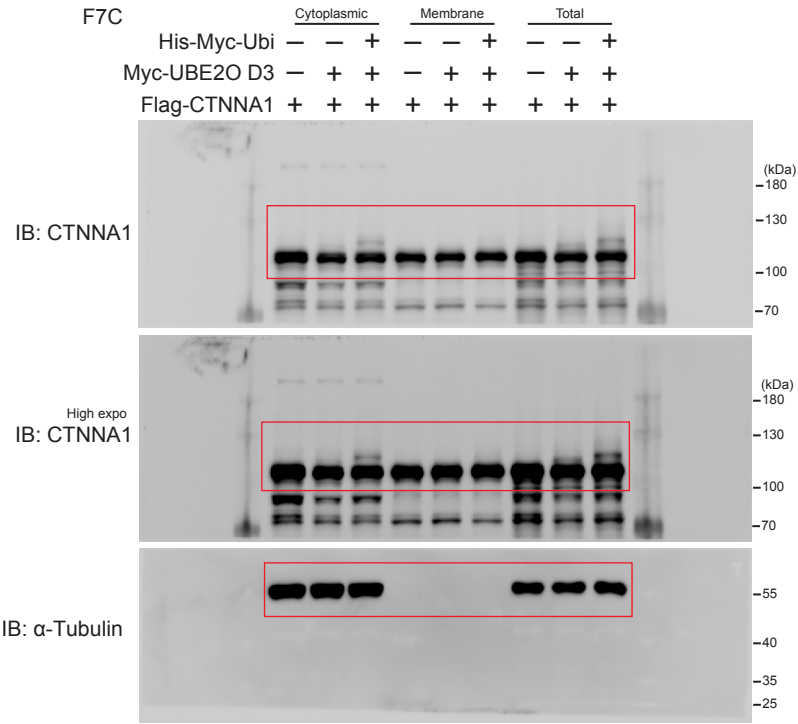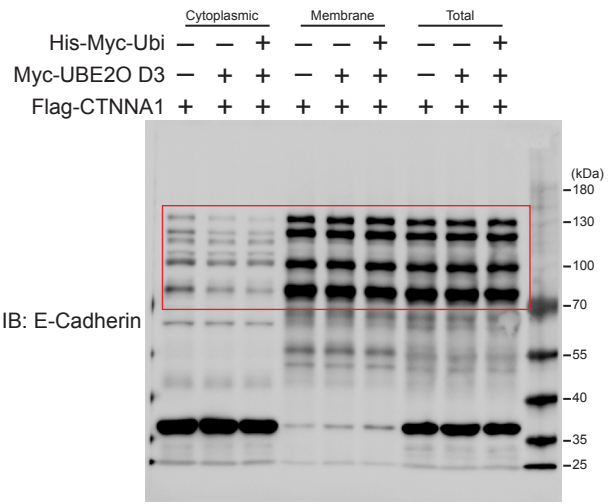

Supplement: Supplementary file 11 — Source data Fig. 7 [file 44319_2025_585_MOESM11_ESM.zip › EMBOR202561827V2_SourceDataForFigure7/7C/Figure7C_Blots.pdf]

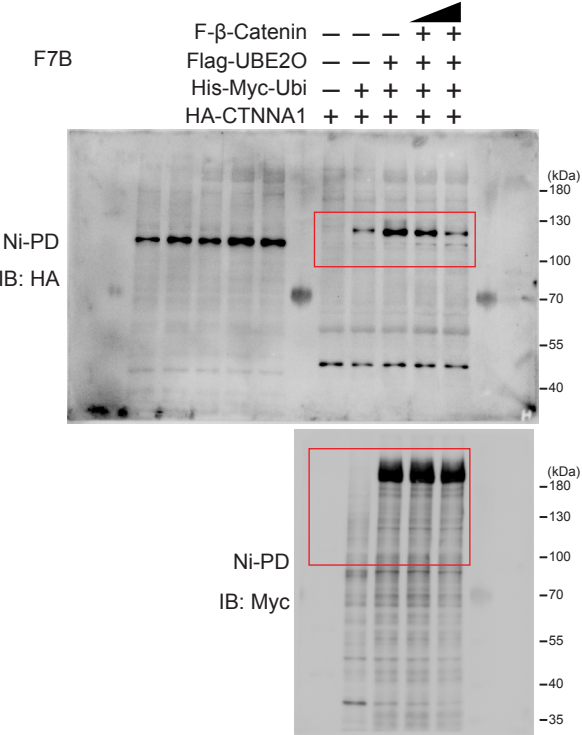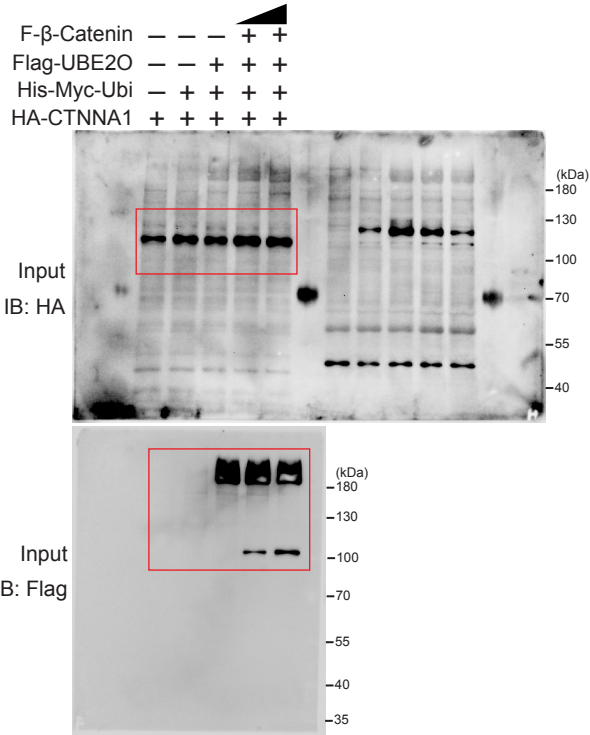

Supplement: Supplementary file 11 — Source data Fig. 7 [file 44319_2025_585_MOESM11_ESM.zip › EMBOR202561827V2_SourceDataForFigure7/7B/Figure7B_Blots.pdf]

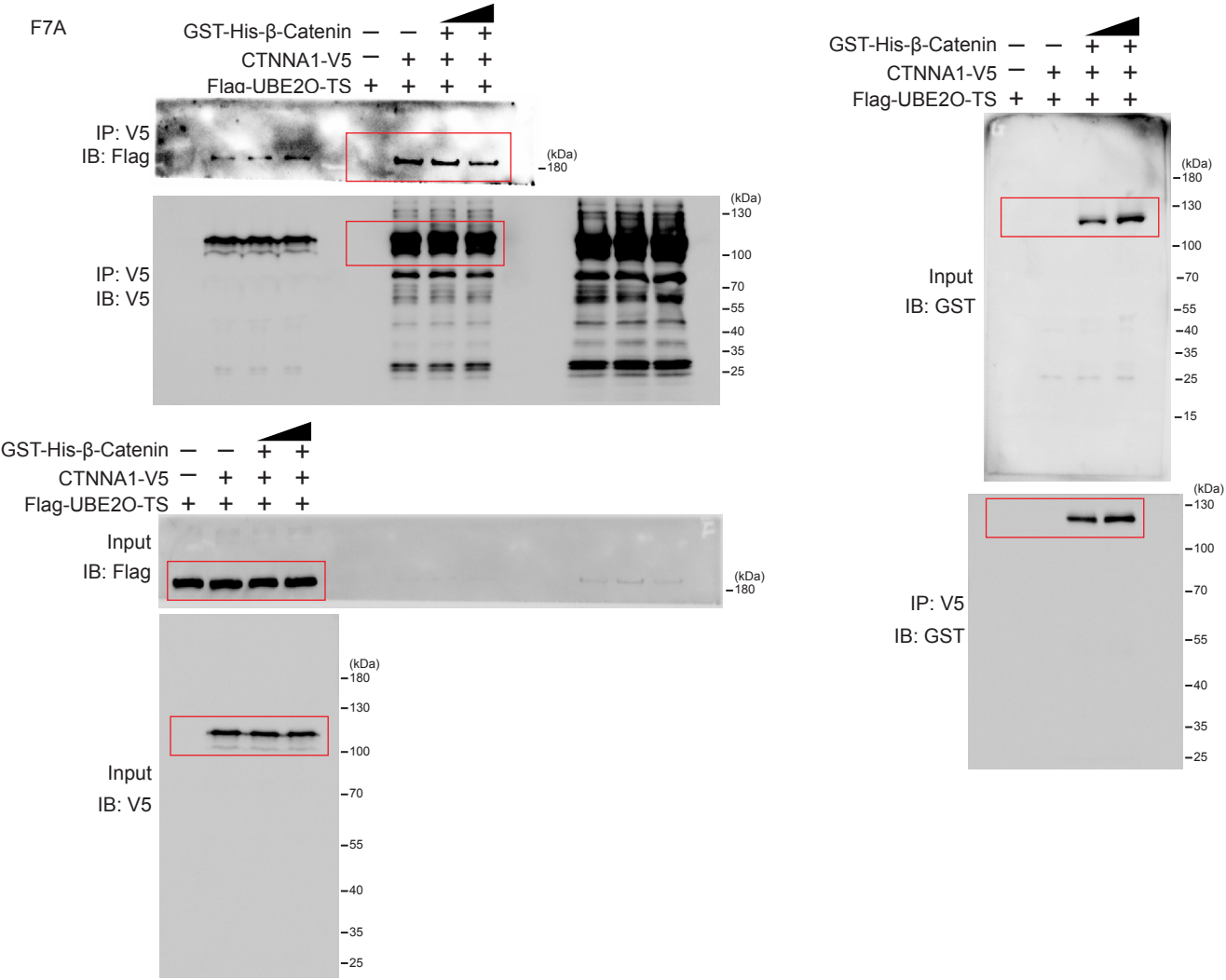

Supplement: Supplementary file 11 — Source data Fig. 7 [file 44319_2025_585_MOESM11_ESM.zip › EMBOR202561827V2_SourceDataForFigure7/7A/Figure7A_Blots.pdf]
