# Supplementary material for: UBE2O-mediated ubiquitylation directs cytoplasmic CTNNA1 to promote cell-to-ECM adhesions
Source: EMBO Rep. 2025 Sep 22;26(22):5431–58. doi: 10.1038/s44319-025-00585-4 (PMC12635394; doi:10.1038/s44319-025-00585-4)

SourceDataF2A_Blots

F2A

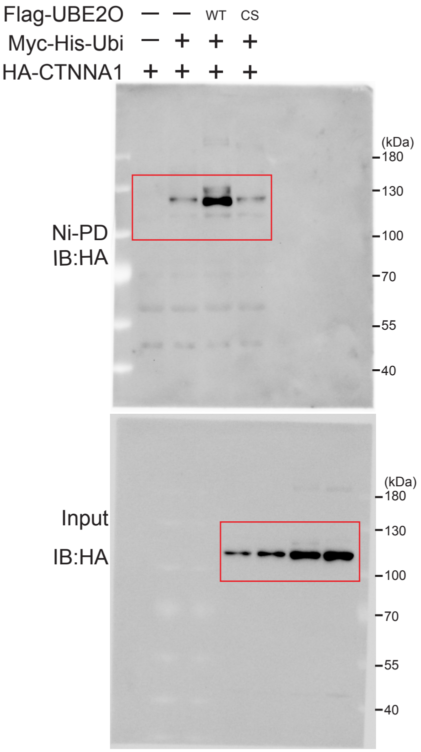

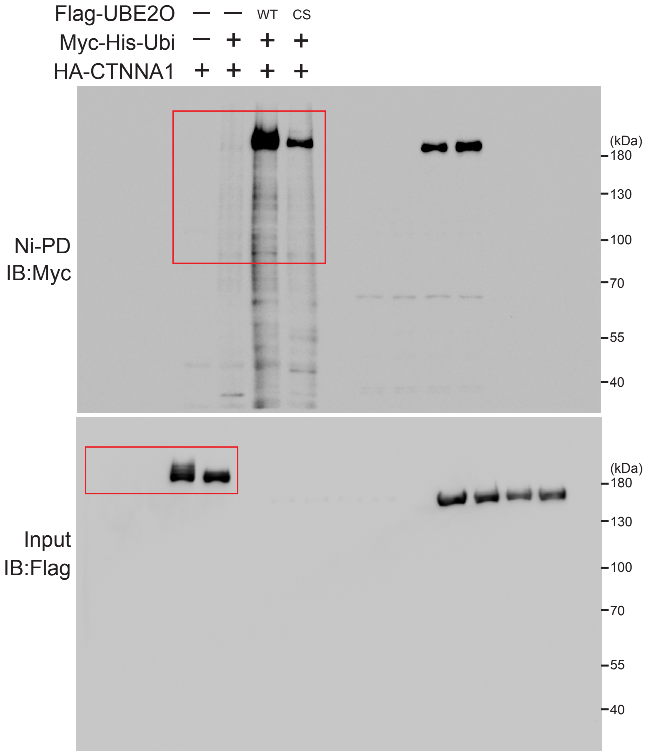

Supplement: Supplementary file 6 — Source data Fig. 2 [file 44319_2025_585_MOESM6_ESM.zip › EMBOR202561827V2_SourceDataForFigure2/2A/Figure2A_Blots.pdf]
