# Supplementary material for: UBE2O-mediated ubiquitylation directs cytoplasmic CTNNA1 to promote cell-to-ECM adhesions
Source: EMBO Rep. 2025 Sep 22;26(22):5431–58. doi: 10.1038/s44319-025-00585-4 (PMC12635394; doi:10.1038/s44319-025-00585-4)
Supplement: Supplementary file 13 — Expanded View Figures [file 44319_2025_585_MOESM13_ESM.pdf]

Expanded View Figures

Figure EV1. Catalytic activity of UBE2O is required for adhesion maturation.

(**A**) CTNNA1 is required for FAs maturation. Representative micrographs of MEFs transfected with siRNAs targeting *Ctnna1* (si*Ctnna1*) or a negative control (siNC) for 48 h. The cells were stained for zyxin after spreading on fibronectin (FN)-coated 8-cell chamber slides for 6 h. (**B**) Western blot analysis of MEFs stably expressing GFP-vector or GFP-zyxin. (**C**, **D**) UBE2O is required for FAs maturation in MEFs. (**C**) Representative micrographs of MEFs treated with doxycycline for 48 h to express shRNAs targeting *Ube2o* UTR region (1#) or CDS region (2#), alongside a negative control targeting luciferase (sh*Luc*). After 6 h of spreading on FN-coated 8-cell chamber slides, cells were stained for zyxin. (**D**) Quantifications of the number of FAs per cell (zyxin channel), average FA size (zyxin channel) and cell area (zyxin channel) of the *Ube2o* knockdown MEFs 6 h after plating on FN-coated 8-cell chamber slides ($n = 7$ cells in all cases). Error bars indicate mean ± SEM. Significance among multiple groups was determined using ANOVA followed by Tukey's post hoc test. (**E**) The catalytic activity of UBE2O is required for FAs maturation and cell extension in MEFs. Representative micrographs of *Ube2o* knockdown MEFs with or without rescuing the expression of UBE2O-WT, UBE2O-CS mutant, or a control vector. All slides were stained for zyxin to visualize FAs and phalloidin to visualize cell area after 6 h of spreading on FN-coated 8-cell chamber slides. (**F**) UBE2O is responsible for FAs maturation in MDCK cells and is catalytic activity-dependent. Representative micrographs of *UBE2O* knockdown MDCK cells with or without rescuing the expression of UBE2O-WT, UBE2O-CS mutant or a control vector. All slides were stained for zyxin and phalloidin after 8 h of spreading. Experiments were repeated at least twice, one representative result is shown.

© The Author(s)

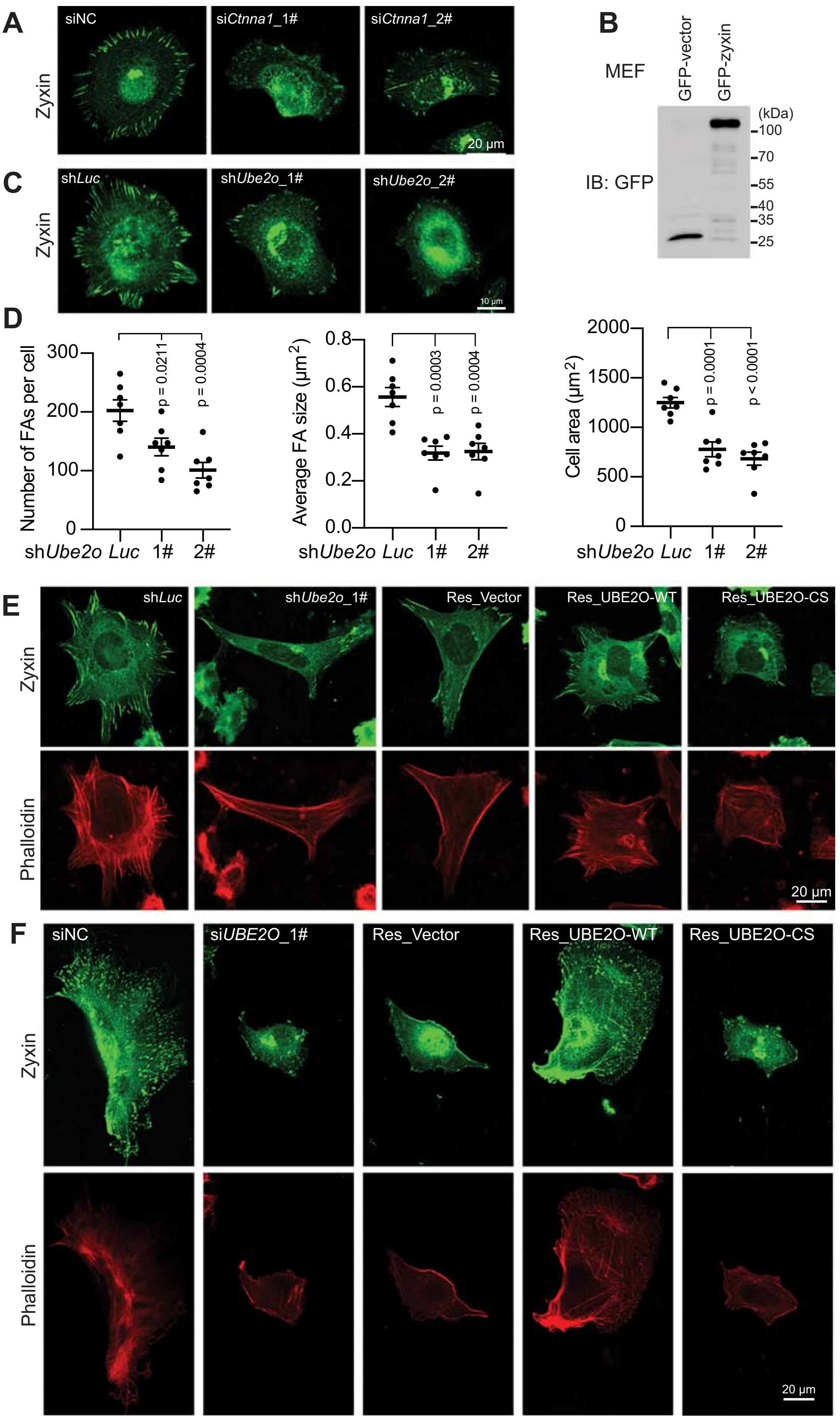
A
siNC
siCtnna1_1#
siCtnna1_2#
Zyxin
20 μm
B
MEF
GFP-vector
GFP-zyxin
(kDa)
100
70
55
40
35
25
IB: GFP
C
shLuc
shUbe2o_1#
shUbe2o_2#
Zyxin
10 μm
D
Number of FAs per cell
p = 0.0211
p = 0.0004
shUbe2o Luc 1# 2#
Average FA size (μm2)
p = 0.0003
p = 0.0004
Cell area (μm2)
p = 0.0001
p < 0.0001
E
shLuc
shUbe2o_1#
Res_Vector
Res_UBE2O-WT
Res_UBE2O-CS
Zyxin
Phalloidin
20 μm
F
siNC
siUBE2O_1#
Res_Vector
Res_UBE2O-WT
Res_UBE2O-CS
Zyxin
Phalloidin
20 μm

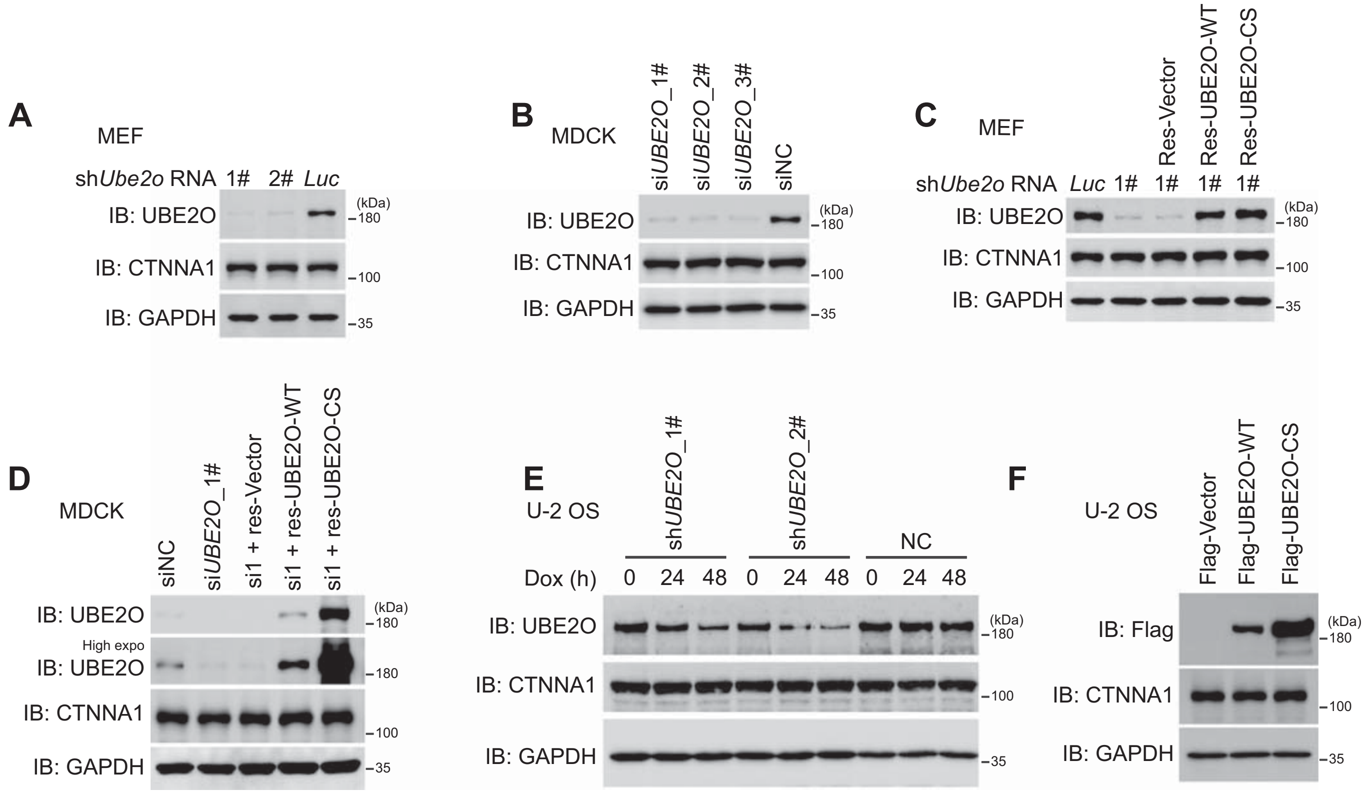

Figure EV2. UBE2O-mediated ubiquitylaton has no effects on the stability of CTNNA1.

(**A**) Knockdown of *Ube2o* in MEFs results in no significant change in CTNNA1 protein level. MEFs were treated with 500 ng/mL doxycycline for 48 h to express shRNAs targeting *Ube2o* UTR region (1#) and CDS region (2#) and a negative control targeting luciferase (sh*Luc*). Cells were harvested for western blot analyses. (**B**) Knockdown *UBE2O* expression in MDCK cells results in no significant change in CTNNA1 protein level. MDCK cells transfected with siNC and si*UBE2O* RNAs for 48 h were harvested for western blot analyses. (**C**, **D**) Re-introducing UBE2O into UBE2O-deficient cells had no considerable effect on CTNNA1 protein level. (**C**) *Ube2o* knockdown MEFs with or without rescuing the expression of UBE2O-WT, UBE2O-CS or a control vector were harvested for western blot analyses. (**D**) MDCK cells with or without stably expressing UBE2O-WT, UBE2O-CS mutant and a control vector were transfected with siNC and si*UBE2O* RNAs as indicated for 48 h. Cells were harvested for western blot analyses. (**E**) Gradual knockdown of *UBE2O* in U-2 OS cells led to no significant regulation of CTNNA1 protein level. U-2 OS cells expressing inducible shRNAs targeting *UBE2O* and a negative control (NC) were treated with 500 ng/mL doxycycline (Dox) for 0 h, 24 h and 48 h to achieve gradual knockdown of *UBE2O*. Cells were harvested for western blot analyses. (**F**) Ectopic expression of UBE2O in U-2 OS cells results in no notable change in CTNNA1 protein level. U-2 OS cells ectopically expressing Flag-UBE2O-WT, Flag-UBE2O-CS mutant, or Flag-vector were harvested for western blot analyses. An anti-CTNNA1 antibody was used to detect endogenous CTNNA1 for all experiments. All experiments were repeated at least twice, one representative result is shown.

 © The Author(s)

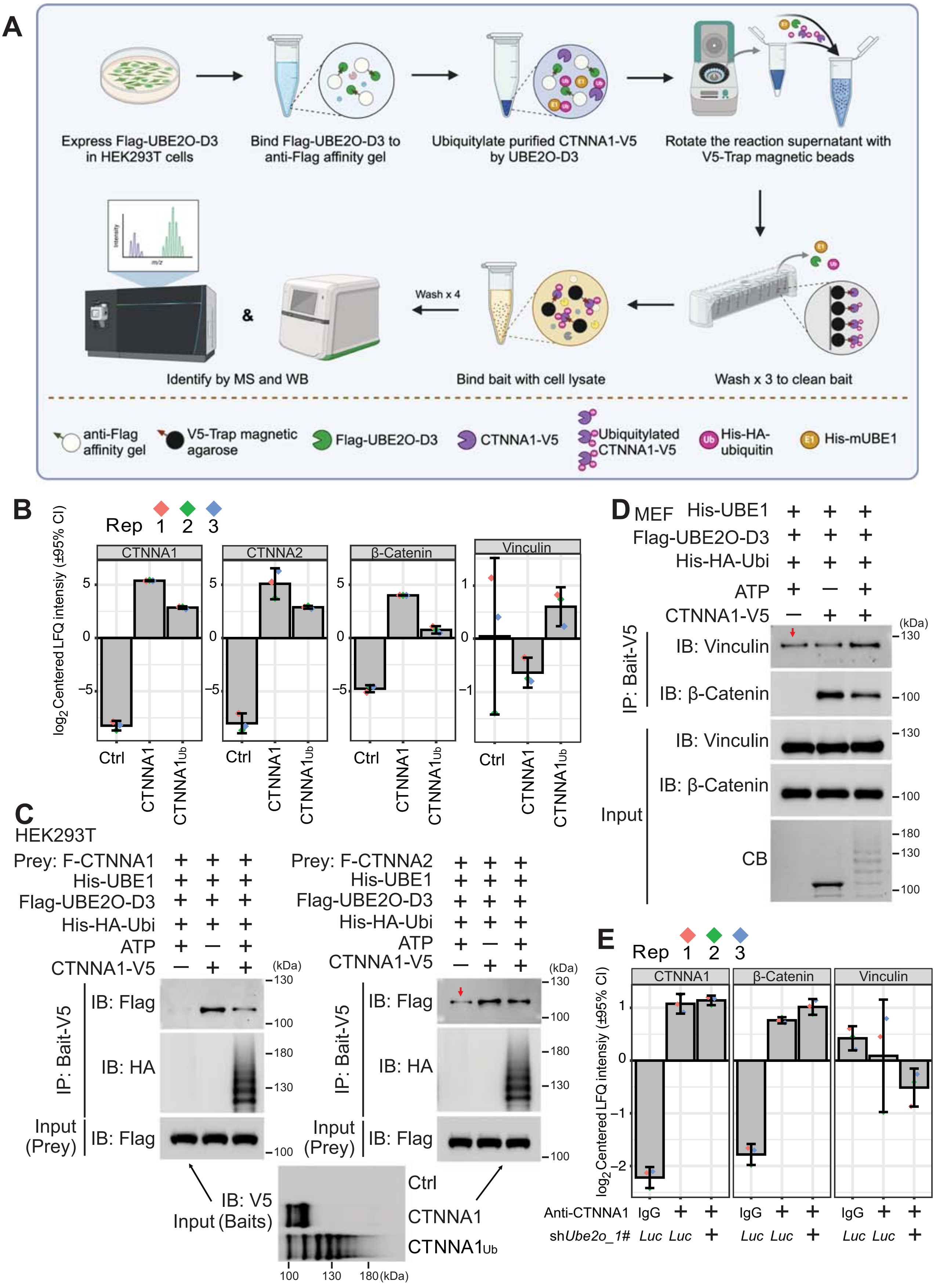
A
Express Flag-UBE2O-D3 in HEK293T cells
Bind Flag-UBE2O-D3 to anti-Flag affinity gel
Ubiquitylate purified CTNNA1-V5 by UBE2O-D3
Rotate the reaction supernatant with V5-Trap magnetic beads
Wash x 3 to clean bait
Bind bait with cell lysate
Wash x 4
&
Identify by MS and WB
anti-Flag affinity gel
V5-Trap magnetic agarose
Flag-UBE2O-D3
CTNNA1-V5
Ubiquitylated CTNNA1-V5
His-HA-ubiquitin
His-mUBE1
B
Rep 1 2 3
log2 Centered LFQ intensiy (±95% CI)
CTNNA1
CTNNA2
β-Catenin
Vinculin
Ctrl
CTNNA1
CTNNA1Ub
C
HEK293T
Prey: F-CTNNA1
Prey: F-CTNNA2
His-UBE1
Flag-UBE2O-D3
His-HA-Ubi
ATP
CTNNA1-V5
IP: Bait-V5
IB: Flag
IB: HA
Input (Prey)
IB: V5
Input (Baits)
Ctrl
CTNNA1
CTNNA1Ub
(kDa)
D
MEF
His-UBE1
Flag-UBE2O-D3
His-HA-Ubi
ATP
CTNNA1-V5
IP: Bait-V5
IB: Vinculin
IB: β-Catenin
Input
CB
E
Rep 1 2 3
log2 Centered LFQ intensiy (±95% CI)
CTNNA1
β-Catenin
Vinculin
Anti-CTNNA1 IgG + +
shUbe2o_1# Luc Luc +

Figure EV3. UBE2O-mediated ubiquitylation of CTNNA1 alters its interactome.

(**A**) Schematic diagram showing the procedure to identify the interactome of ubiquitylated CTNNA1. Detailed description is shown in the method. This diagram was created in BioRender. (**B**) Barplot showing the log_2 Centered LFQ (label free quantification) intensity (±95% CI) of CTNNA1, CTNNA2, β-catenin and vinculin identified in ubiquitylated CTNNA1 interactome. Data were visualized using the DEP2 package, $n = 3$ technical replicates in independent experiments, error bars indicate mean ± SD. (**C**) Validation of the interaction between ubiquitylated CTNNA1 with itself or CTNNA2 by immunoprecipitation coupled with immunoblotting experiments in HEK293T cells. The ctrl, CTNNA1 and ubiquitylated CTNNA1 ($CTNNA1_{Ub}$) baits were aliquoted into 2 tubes equally and incubated with cell lysate of HEK293T cells transiently expressing Flag-CTNNA1 or Flag-CTNNA2 overnight. The proteins bound to beads were eluted and identified by immunoblotting using an anti-Flag antibody. An anti-V5 antibody was used to confirm the successful reconstitution of the baits. The red arrow indicates unspecific binding to the beads. (**D**) Validation of the interaction between ubiquitylated CTNNA1 and vinculin or β-catenin by immunoprecipitation coupled with immunoblotting experiments in MEFs. The ctrl, CTNNA1 and ubiquitylated CTNNA1 baits were incubated with MEF cell lysate overnight, the bound proteins were eluted and identified by immunoblotting using anti-vinculin and anti-β-catenin antibodies. Coomassie blue staining (CB) was used to confirm the successful reconstitution of the baits. The band indicated by the red arrow indicates unspecific binding to magnetic beads. (**E**) Barplot showing the log_2 Centered LFQ (label free quantification) intensity (± 95% CI) of CTNNA1, β-catenin and vinculin identified in endogenous CTNNA1 interactome upon *Ube2o* knockdown. Data were visualized using the DEP2 package, $n = 3$ technical replicates in independent experiments, error bars indicate mean ± SD. Experiments were repeated at least twice, one representative result is shown.

© The Author(s)

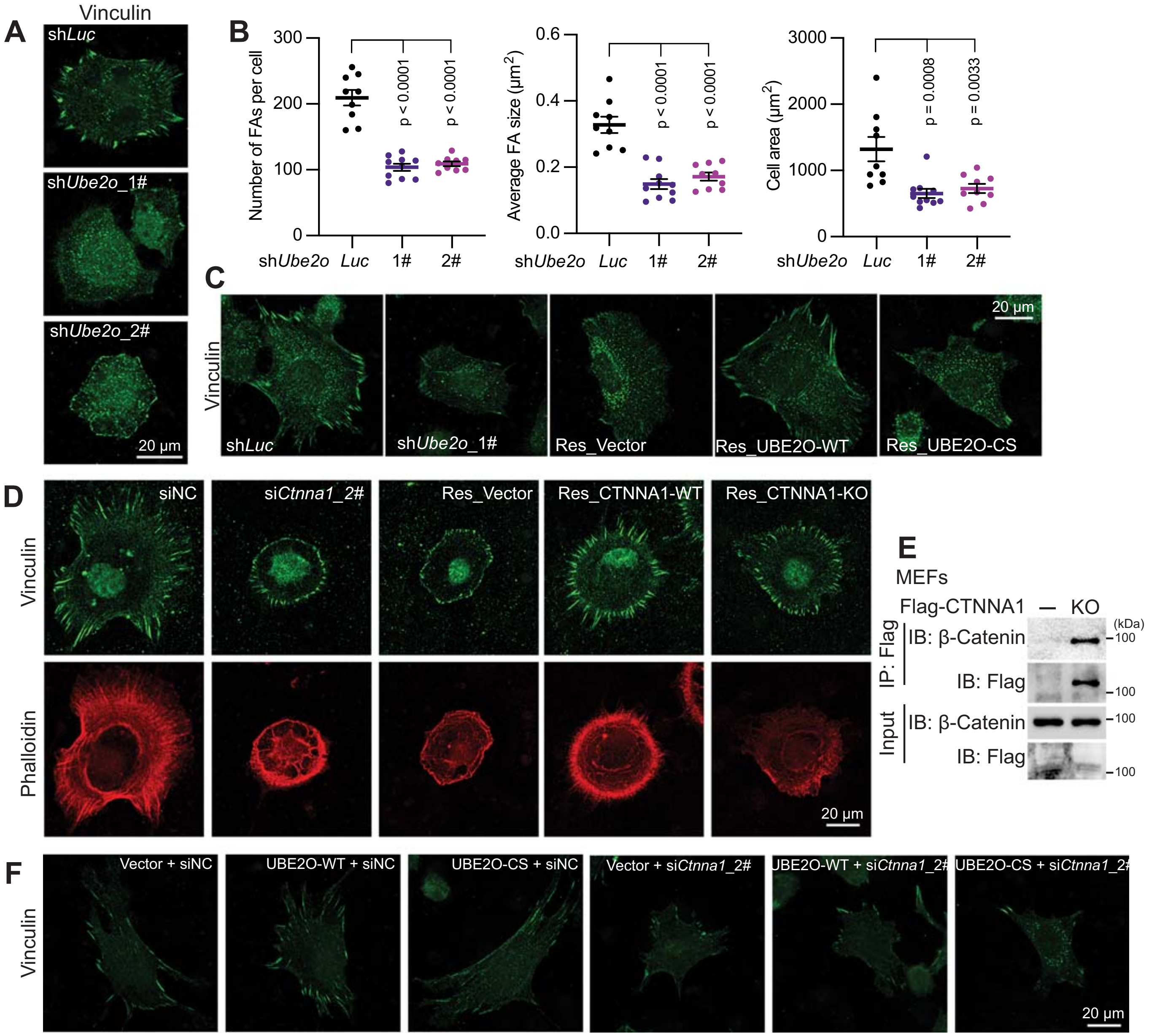

Figure EV4. UBE2O-mediated monoubiquitylation of CTNNA1 is required for vinculin-containing FAs maturation and cell extension.

(**A**, **B**) Knockdown of *Ube2o* impedes vinculin-containing FAs maturation. (**A**) Representative micrographs of MEFs treated with doxycycline for 48 h to express shRNAs targeting *Ube2o* UTR region (1#) or CDS region (2#) and a negative control targeting luciferase (sh*Luc*). After 6 h of spreading on FN-coated 8-cell chamber slides, cells were stained for vinculin. (**B**) Quantifications of the number of FAs per cell (vinculin channel), average FA size (vinculin channel) and cell area (vinculin channel) of the *Ube2o* knockdown MEFs 6 h after plating on FN-coated 8-cell chamber slides. Total number of cells analyzed: sh*Luc* (9), sh1 (10), sh2 (9). Error bars indicate mean ± SEM. Significance among multiple groups was determined using ANOVA followed by Tukey's post hoc test. (**C**) The catalytic activity of UBE2O is responsible for vinculin-containing FAs maturation. Representative micrographs of *Ube2o* knockdown MEFs with or without rescuing the expression of UBE2O-WT, UBE2O-CS mutant, or a control vector. All slides were stained for vinculin after 6 h of spreading on FN-coated 8-cell chamber slides. (**D**) Ubiquitylation of CTNNA1 is engaged in vinculin-containing FAs maturation and cell extension. Representative micrographs of *Ctnna1* knockdown MEFs with or without rescuing the expression of CTNNA1-WT, CTNNA1-KO mutant or a control vector. All slides were stained for vinculin and phalloidin after 6 h of spreading on FN-coated 8-cell chamber slides. (**E**) Immunoblot shows stably expressed Flag-CTNNA1-KO interacts with endogenous β-catenin in MEFs. (**F**) UBE2O regulates vinculin-containing FAs maturation via CTNNA1. Representative micrographs of MEFs expressing UBE2O-WT, UBE2O-CS mutant or a control vector transfected with a siRNA targeting *Ctnna1* or a negative control (siNC) for 48 h. All slides were stained for vinculin after 6 h of spreading on FN-coated 8-cell chamber slides. All experiments were repeated at least twice, one representative result is shown.

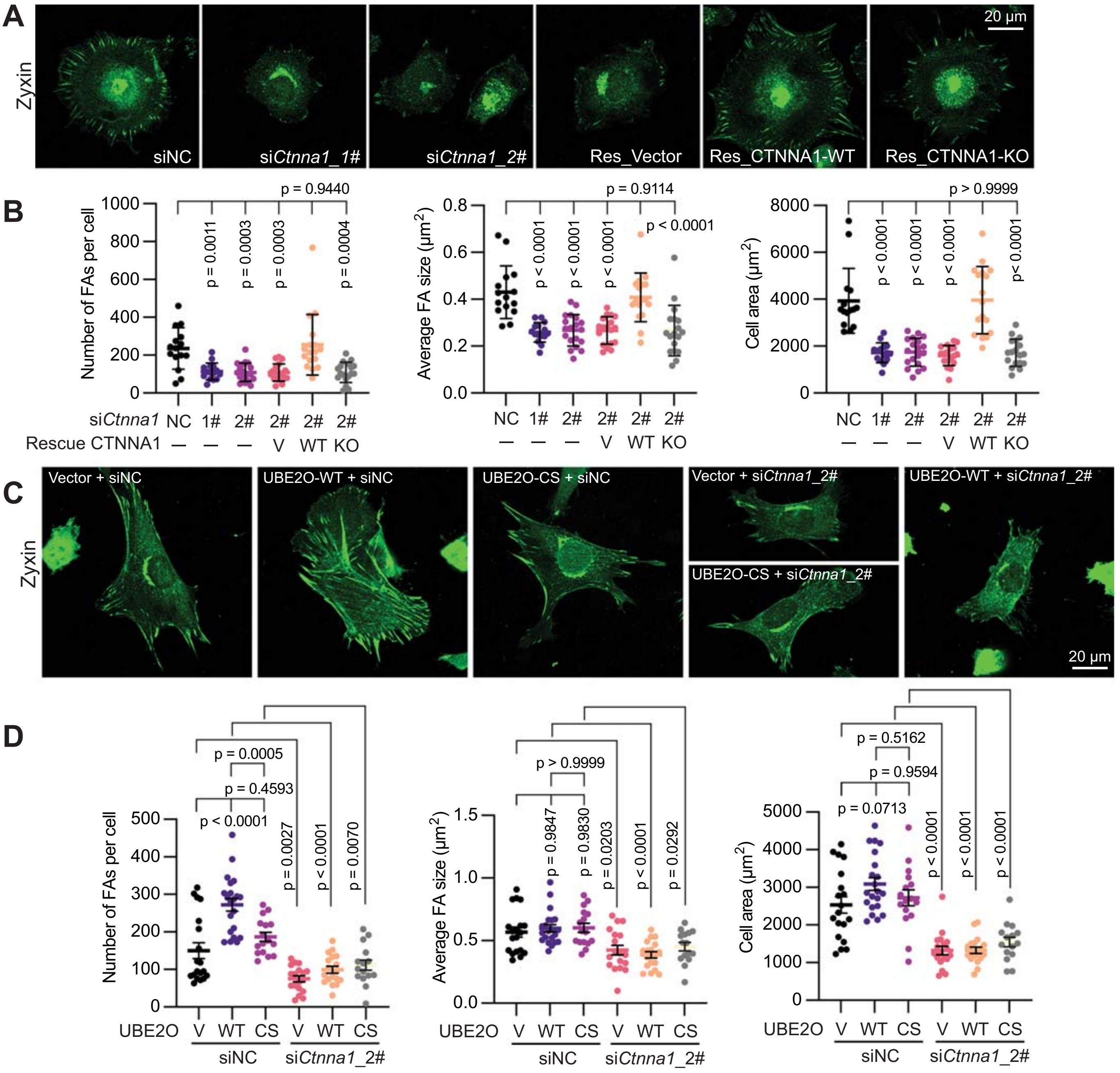

Figure EV5. UBE2O-mediated monoubiquitylation of CTNNA1 is required for zyxin-containing FAs maturation and cell extension.

(**A**, **B**) Ubiquitylation of CTNNA1 participates in zyxin-containing FAs maturation. (**A**) Representative micrographs of *Ctnna1* knockdown MEFs with or without rescuing the expression of CTNNA1-WT, CTNNA1-KO mutant or a control vector, all slides were stained for zyxin after 6 h of spreading on FN-coated 8-cell chamber slides. (**B**) Quantifications of the number of FAs per cell (zyxin channel), average FA size (zyxin channel) and cell area (zyxin channel) of the indicated MEFs 6 h after plating on FN-coated 8-cell chamber slides. Total number of cells analyzed: siNC (15), si*Ctnna1*_1# (15), si*Ctnna1*_2# (19), Res_Vector (18), Res_CTNNA1-WT (17), Res_CTNNA1-KO (18). (**C**, **D**) UBE2O regulates zyxin-containing FAs maturation via CTNNA1. (**C**) Representative micrographs of MEFs expressing UBE2O-WT, UBE2O-CS mutant or a control vector transfected with a siRNA targeting *Ctnna1* or a negative control (siNC) for 48 h, all slides were stained for zyxin after 6 h of spreading on FN-coated 8-cell chamber slides. (**D**) Quantifications of the number of FAs per cell (zyxin channel), average FA size (zyxin channel) and cell area (zyxin channel) of the indicated MEFs 6 h after plating on FN-coated 8-cell chamber slides. Total number of cells analyzed: Vector + siNC (19), UBE2O-WT + siNC (21), UBE2O-CS + siNC (16), Vector + si*Ctnna1*_2# (18), UBE2O-WT + si*Ctnna1*_2# (18), UBE2O-CS + si*Ctnna1*_2# (15). Error bars indicate mean ± SEM. Significance among multiple groups was determined using ANOVA followed by Tukey's post hoc test. All experiments were repeated at least twice, one representative result is shown.

 © The Author(s)
